# A Gut-Restricted Liver X Receptor Agonist Ameliorates Liver Injury in Experimental Short Bowel Syndrome

**DOI:** 10.1053/j.gastro.2025.12.015

**Published:** 2026-03-06

**Authors:** Ayoung Kim, Daniel M. Alligood, Lingaiah Maram, Hannah M. Phelps, Michael Cameron, Jacob T. DeRousse, Jichang Han, Taylor J. Dunning, Rachel L. Mintz, Alex Park, Daniel D. Lee, Deanna L. Davis, Christopher G. Huckstep, Rachael L. Field, Lamees Hegazy, Bernd H. Zinselmeyer, Jonathan R. Brestoff, Colin A. Martin, Brad W. Warner, Bahaa Elgendy, Gwendalyn J. Randolph

**Affiliations:** 1 Department of Pathology and Immunology, Washington University School of Medicine, St Louis, Missouri; 2 Department of Surgery, Washington University School of Medicine, St Louis, Missouri; 3 Department of Anesthesiology, Washington University School of Medicine in St Louis, St Louis, Missouri; 4 Center for Clinical Pharmacology, Washington University School of Medicine and University of Health Sciences and Pharmacy, St Louis, Missouri; 5 Department of Molecular Medicine, University of Florida, Jupiter, Florida; 6 Department of Biomedical Engineering, Washington University in St Louis, St Louis, Missouri; 7 Department of Pharmaceutical and Administrative Sciences, University of Health Sciences and Pharmacy, St Louis, Missouri

**Keywords:** Liver X Receptors, Enterohepatic Circulation, Lipid Transport, Fibrosis, Inflammation

## Abstract

**BACKGROUND & AIMS:**

Short bowel syndrome (SBS) arises from the surgical removal of extensive portions of the small intestine and is associated with high morbidity, including intestinal failure–associated liver disease (IFALD). Earlier studies revealed that orally administered systemic liver X receptor (LXR) agonist suppresses IFALD in mice and implicated intestinally derived high-density lipoprotein (HDL) in liver protection. Here we aimed to move away from the use of systemic LXR agonists because they have failed in clinical trials due to hepatic steatosis and hyperlipidemia, to determine if a gut-restricted LXR agonist could provide hepatoprotection in SBS.

**METHODS:**

We synthesized and characterized WUSTL0717, an amide analog of GW3965, as a putative gut-restricted LXR agonist, and evaluated its potential to improve the outcomes in a preclinical mouse model of SBS.

**RESULTS:**

WUSTL0717 exhibited exceptional intestinal retention in pharmacokinetic analyses and activated LXR target genes in the small intestine but not the liver. Whereas small bowel resection lowered many lipid metabolites in portal venous plasma, WUSTL0717 treatment increased portal venous Apolipoprotein A1 (ApoA1), the core protein of HDL, and spared portal venous phospholipids known to be enriched on HDL. Accordingly, intestinal ApoA1 deficiency exacerbated IFALD, and in wild-type mice, portal venous ApoA1 and phospholipids inversely correlated with hepatic collagen accumulation. In addition, WUSTL0717 improved nutrient absorption and promoted body weight restoration in SBS.

**CONCLUSIONS:**

These data underscore the potential of gut-restricted LXR agonists to preserve metabolic health in the context of SBS. By acting locally in the intestine, WUSTL0717 positively mitigates profibrotic liver injury while avoiding systemic availability.

## Graphical Abstract

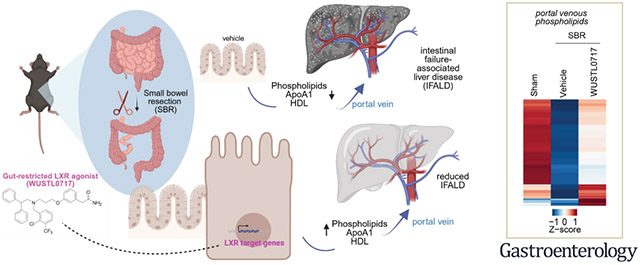

The small intestine absorbs nutrients to sustain life. Various conditions, including neonatal necrotizing enterocolitis, may require small bowel resection (SBR) that can lead to malabsorption^1^ and partial compensatory post-intestinal adaptations,^2,3^ sequelae collectively termed short bowel syndrome (SBS).^1^ Prolonged SBS leads to morbidity in the liver, referred to as intestinal failure–associated liver disease (IFALD), which involves hepatic steatosis, cholestasis, and fibrosis.^4^ Glucagon-like peptide-2 agonists are used clinically to improve intestinal adaptation (NCT05018286). However, no established therapeutics exist for SBS-associated IFALD once it develops, making prevention the primary management goal. Approaches under investigation for IFALD management include omega-3 enriched parenteral lipids^5^ and the medium-chain fatty acid cocktail SEFA-6179 (NCT05919680).^6^

Liver X receptors (LXRs), including LXR*α* (*NR1H3*) and LXR*ß* (*NR1H2*), are nuclear receptors that govern genes involved in lipid and sterol metabolism.^7^ We previously reported that intestinal ATP-binding cassette transporter A1 (*Abca1*), a key LXR target for high-density lipoprotein (HDL) biogenesis, promoted portal venous HDL that protected the liver from lipopolysaccharide-induced injury.^8^ Oral administration of the LXR agonist GW3965^9^ appeared to have potential therapeutic value by upregulating *Abca1* and increasing portal HDL.^8^ However, systemic LXR agonists cause adverse effects, primarily hepatic lipogenesis, hindering their development as treatments for atherosclerosis and other diseases.^10,11^ Hence, we reasoned that a gut-restricted LXR agonist might offer safer and more effective therapy. In our search for gut-restricted LXR agonists to treat SBS, we identified WUSTL0717. Reported by GlaxoSmithKline in 2002, this LXR agonist was not further developed, as the slightly more potent analog GW3965 was favored.^12^ WUSTL0717 (previously referred to as GW6340) was later described as an intestinally selective compound, with limited published evidence, and as an ester analog of GW3965.^13^ However, the GlaxoSmithKline patent (PCT/US01/27622) refers to GW6340 as an amide analog of GW3965, which we designate as WUSTL0717.

Here, we synthesized and carried out pharmacokinetic studies of WUSTL0717 in mice to evaluate its intestinal retention and its effects on SBS and IFALD. We also examined its impact on metabolites in the portal vein, where we found that it primarily spared a reduction in phospholipids within the portal vein caused by SBR. Phospholipids, particularly phosphatidylcholine (PC) species, are major HDL components along with Apolipoprotein A1 (ApoA1) and cholesteryl esters.^14,15^ We thus evaluated the impact of intestinal epithelial cell deletion of *Apoa1* on SBR. Our data further solidify the key role of intestinal HDL in protecting the liver through its passage in the portal vein and suggest therapeutic benefits of WUSTL0717 for managing IFALD and improving intestinal functionality, with action restricted to inducing LXR target genes specifically within the intestine.

## Materials and Methods

### Mice

C57BL/6 wild-type (WT) (JAX 000664) mice were purchased from The Jackson Laboratory. Mice generated by crossing *Villin-Cre* (JAX 004586) mice with *Apoa1^f/f^* mice (*Apoa1^ΔIEC^*)^16^ were on a mixed genetic background, predominantly C57BL/6 with additional contributions from FVB/NJ or DBA/2J. Mice were housed at Washington University in St Louis in a 12-hour light-dark cycle in a specific pathogen-free facility with a controlled temperature (21 ± 1° C) and humidity (50% ± 20%). Food and water were provided ad libitum unless otherwise specified. Genotypes were confirmed by polymerase chain reaction using primer pairs listed in Supplementary Table 1, and the absence of Cre-mediated germline recombination was verified by Transnetyx qPCR. All procedures were conducted during the light phase. Age- and weight-matched mice were randomly divided into experimental subgroups based on the distribution. Experimenters were not blinded to genotypes or treatment due to the study design and group size. The controls in all experiments were sibling littermates of the experimental cohort, and siblings, even when of different genotypes, were co-housed throughout all experiments. The studies were approved by the Washington University Animal Studies Committee (protocols 22-0433, 23-0421, and 22-0286).

### Synthesis of WUSTL0717, Molecular Modeling and Screening, and Use In Vivo

A detailed synthesis protocol for WUSTL0717 is described at the accompanying submission in protocols.io (“Synthesis of WUSTL0717” available at: https://www.protocols.io/view/synthesis-of-wustl0717-bp2l6dy2kvqe/v1). Molecular modeling, evaluation, and ligand-binding assays are described in the Supplementary Materials and Methods. For in vivo administration, WUSTL0717.HCl was suspended in 0.5% hydroxypropyl methylcellulose and 4% Tween 80 in 60 mM phosphate buffer (pH 7). WUSTL0717 or vehicle was administered orally (per os [PO]) by gavage once daily at 30 mg/kg body weight, starting either 1 week after arrival at the facility or 3 weeks after SBR or sham surgery to allow recovery. For short-term WUSTL0717 administration, mice were treated with WUSTL0717 starting 5 days post-SBR and continued for 10 days. In nonsurgical studies, WUSTL0717 treatment was initiated in WT mice at 8 weeks of age.

### Pharmacokinetics

At the specified time point after administration of a single oral gavage dose of WUSTL0717 at 30 mg/kg, 8-week-old WT male mice were euthanized and dissected tissues or plasma were snap-frozen in liquid nitrogen. Untreated mice served as baseline control. For quantification, 5-*μ*L plasma samples were directly loaded onto a 96-well Millipore Multiscreen Solvinert 0.45-*μ*m low-binding polytetrafluoroethylene hydrophilic filter plate. Tissue samples were homogenized with water (1:3 dilution), and 5 *μ*L of the homogenate was loaded onto the filter plate. All plasma and tissue samples were treated with 75 *μ*L of a 90/10 acetonitrile/water solution containing carbamazepine as the internal standard to extract the analyte and precipitate proteins. The plates were agitated on ice for 10 minutes before centrifugation into a collection plate. Separate standard curves were prepared in blank mouse plasma and tissue homogenate and processed in parallel with the samples. The filtrate was directly analyzed by liquid chromatography–tandem mass spectrometry (LC-MS/MS). The high-performance liquid chromatography and MS/MS parameters are provided in Supplementary Table 2. C_max_ (maximum concentration) and T_max_ (time to maximum concentration) were determined from the observed concentration–time data.

### Small Bowel Resection

Unless otherwise specified, 8- to 9-week-old male or female mice were subjected to SBR. Mice underwent 75% proximal bowel resection or a sham control operation, as previously described.^8^ Briefly, a midline laparotomy was performed to exteriorize the small bowel. In SBR, the small bowel was cut 1 to 2 cm distal to the ligament of Treitz and 6 cm proximal to the ileocecal junction, then reconnected by an anastomosis. In sham operations, the small bowel was transected 6 cm proximal to the ileocecal junction and immediately re-anastomosed. All anastomoses were hand-sewn end-to-end with interrupted 9–0 nylon sutures. After surgery, the mice received a 1-mL bolus of normal saline and were placed in a warm cage inside an incubator. During the night following surgery, they were restricted to water only. On postoperative day 1, a second 1-mL bolus of saline was administered, and a liquid diet (PMI Micro-Stabilized Rodent Liquid Diet LD 101; TestDiet) was introduced. Mice were without food for less than 24 hours after surgery. They remained in the incubator until postoperative day 7, then were moved to standard cages with continued ad libitum access to the liquid diet until euthanasia.

### Blood Chemistry and Tissue Lipid Measurements

Portal venous blood (60 *μ*L) was collected with needle bevel facing the intestine, and systemic blood was collected from the inferior vena cava or via cheek bleed. For plasma isolation, blood was collected in EDTA tubes and centrifuged at 1000*g* for 10 minutes at 4° C. For serum isolation, coagulated blood was centrifuged at 2000*g* for 15 minutes at room temperature. Plasma or serum cholesterol, triglycerides, alanine aminotransferase (ALT), and aspartate aminotransferase (AST) were assessed by the DCM Research Animal Diagnostic Laboratory at Washington University. HDL-cholesterol (HDL-C) levels were measured using the HDL-C assay kit (STA-394; Cell Biolabs), and ApoA1 levels by enzyme-linked immunosorbent assay (3750–1HP; Mabtech). Liver cholesterol and triglyceride levels were quantified by the Diabetes Models Phenotyping Core Services at Washington University.

### RNA Sequencing

Liver (median right lobe), duodenum, or post-anastomotic ileum from sham- or SBR-operated mice, with or without WUSTL0717 treatment, were subjected to RNA sequencing (RNA-seq) by the Genome Technology Access Center. Data were analyzed as detailed in the Supplementary Materials and Methods and deposited in the Gene Expression Omnibus (accession GSE287046).

### Lipid and Metabolomics LC-MS/MS Analysis

Mouse portal venous serum samples were extracted and analyzed by LC-MS/MS on a Vanquish Horizon UHPLC system coupled to an Orbitrap Tribrid ID-X mass spectrometer (Thermo Fisher Scientific) by the Mass Spectrometry Technology Access Center, as detailed in the Supplementary Materials and Methods.

### Statistics

Data are presented as mean ± standard error of the mean (SEM). Statistical significance was assessed using unpaired Student *t* test or 1-way/2-way analysis of variance followed by Tukey’s honestly significant difference for multiple group comparisons, unless otherwise specified. All statistical analyses were performed using Prism software (GraphPad), except where noted. Statistical approaches for high-dimensional datasets, including RNA-seq, 16S ribosomal RNA sequencing, and lipidomics, are described in their respective subsections in the Supplementary Materials and Methods. Details on significance levels, sample sizes, and specific statistical tests are provided in the figure legends.

## Results

### Synthesis and In Vivo Pharmacokinetics of WUSTL0717 Reveal Intestinal Retention

**Figure 1.**
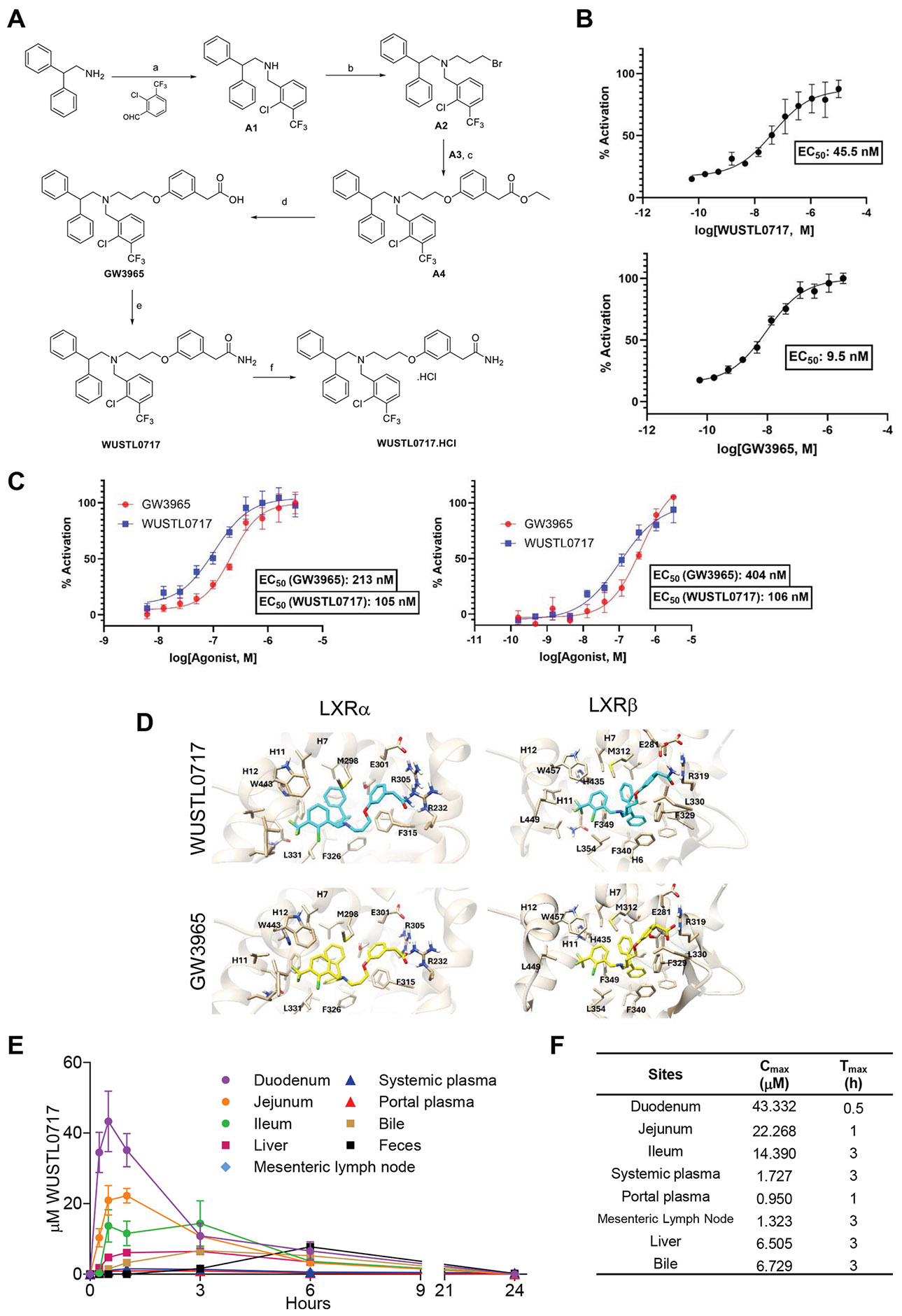
Synthesis overview and pharmacokinetic profile of WUSTL0717. (A) Chemical reaction steps for WUSTL0717 synthesis. Reaction conditions are described in detail in protocols.io (available at: https://www.protocols.io/view/synthesis-of-wustl0717-bp2l6dy2kvqe/v1). (*B* and *C*) Dose-response curve of WUSTL0717 and GW3965 in LanthaScreen LXR-*ß* coactivator assay (*B*) and cell-based LXR*α* and LXR*ß* activation luciferase assay (*C*). Curves were fitted to measurements from 4 wells per concentration, using 4-variable nonlinear regression. (*D*) Binding poses of WUSTL0717 (cyan) in LXR*α* and LXR*ß*; and GW3965 (yellow) in LXR*α* and LXR*ß*. Hydrogen bonding interactions are illustrated as *blue lines*. (E and F) Pharmacokinetic analysis of WUSTL0717. (*E*) Tissue distribution of WUSTL0717 after a single 30-mg/kg oral dose in male mice (n = 5 per time point). Fecal samples from each time point were pooled into 1 sample. Graph depicts mean ± standard error of the mean (SEM). (*F*) Table of the mean values of C_max_ and T_max_.

WUSTL0717 was synthesized in a multi-step process, ensuring high yield and purity^17^ (Figure 1A; details in protocols.io, available at: https://www.protocols.io/view/synthesis-of-wustl0717-bp2l6dy2kvqe/v1). LanthaScreen Time-Resolved Förster Resonance Energy Transfer (TR-FRET) assays revealed that GW3965 exhibited ~5-fold higher potency compared with WUSTL0717 in binding to LXR*ß* (Figure 1B). In contrast, WUSTL0717 demonstrated enhanced activity in a luciferase assay, showing ~2-fold greater potency than GW3965 (Figure 1C). Molecular modeling of WUSTL0717 in the ligand-binding pocket of LXR*α* and LXR*ß* revealed that, similar to GW3965, its chlorotrifluoromethyl benzyl groups primarily formed hydrophobic interactions. However, the amide group of WUSTL0717 exhibited distinct interactions, engaging Arg232 in LXR*α* and with Leu330 and Glu281 in LXR*ß*. In contrast, the carboxyl group of GW3965 interacted with Arg305 and Arg232 in LXR*α*, and with Leu330 and R319 in LXR*ß* (Figure 1D). In vitro absorption, distribution, metabolism, and excretion assessments revealed that WUSTL0717 has very low kinetic solubility (0.29 *μ*M) and high mouse plasma protein binding (99.81%). It showed metabolic stability in mouse liver microsomes (t_1/2_ = 49 minutes), but less stability in human microsomes (t_1/2_ = 12.4 minutes), indicating species-dependent metabolism (Supplementary Table 3). In vivo pharmacokinetic studies, in which a single dose of WUSTL0717 (30 mg/kg) was administered to individual mice and the distribution of the drug followed over a 24-h period, revealed a strong but transient drug signal in the duodenum, followed by the jejunum and ileum. Drug distribution to the liver was detected at levels substantially lower than those in the intestine (Figure 1E and F). Complete clearance of the drug from all sites occurred within 24 hours (Figure 1E and F). At most sites, peak accumulation was observed at 0.5 to 3 h after dosing, and at all sites, except the feces, waned after 3 hours. Fecal detection of WUSTL0717 peaked at 6 hours and waned thereafter (Figure 1E and F). We conclude that orally administered WUSTL0717 is a potent LXR agonist with activity primarily confined to the small intestine before undergoing rapid catabolism.

### WUSTL0717 Activates Liver X Receptor Target Genes in the Intestine But Not the Liver in a Mouse Model of SBS

**Figure 2.**
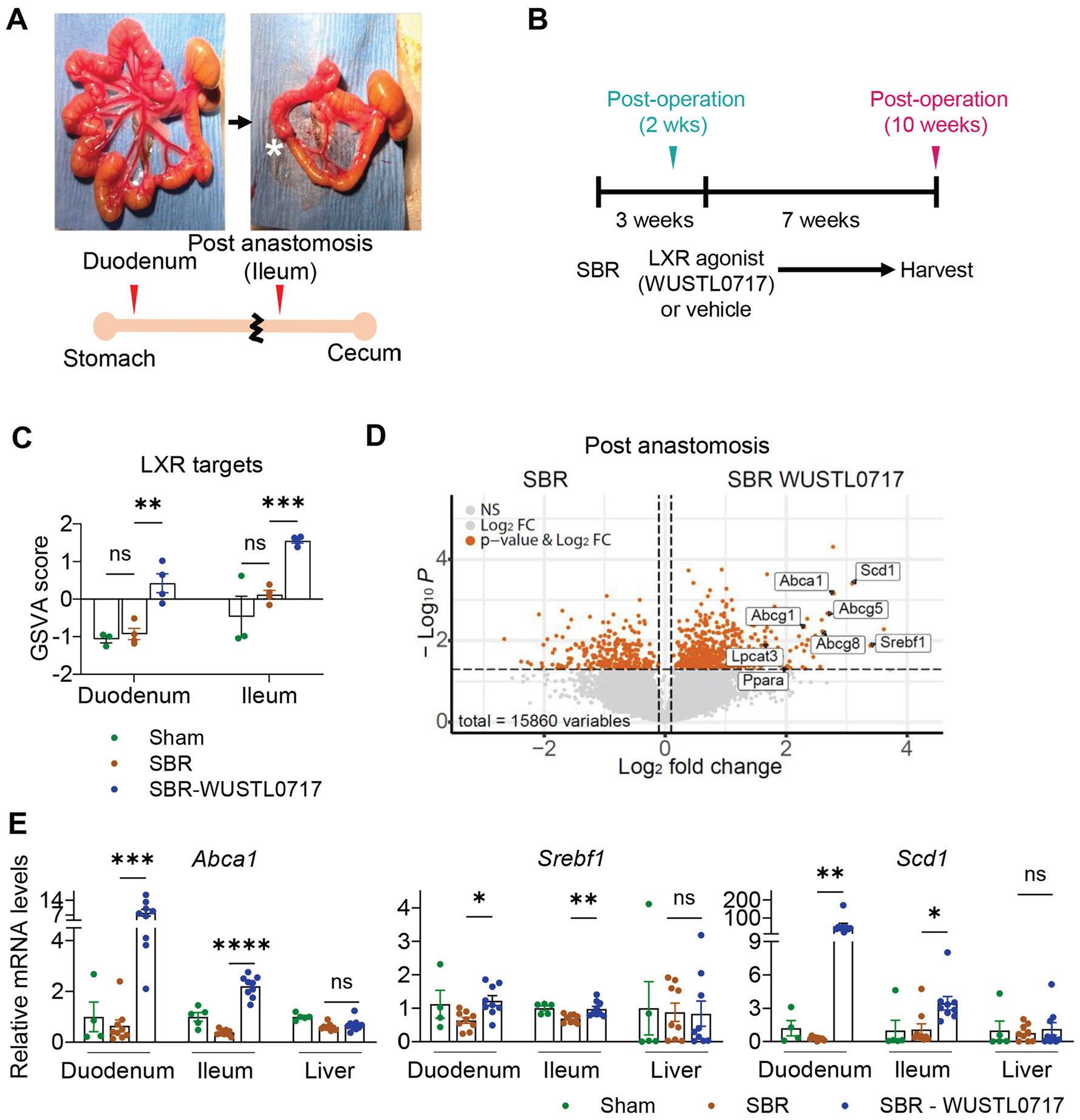
WUSTL0717 exhibits small intestine–restricted LXR agonist activity following SBR. (*A*) Images and schematic representations of the mouse small intestine before and after SBR. *White star*: the anastomosis region. (*B*) Scheme of the SBR procedure and vehicle or WUSTL0717 dosing schedule. WT mice underwent sham or SBR operation. Beginning 3 weeks post-surgery, the mice received either vehicle or WUSTL0717 (30 mg/kg, PO) daily for 7 weeks before euthanasia. (*C*) Gene set variation analysis of LXR target genes from RNA-seq data in male mice subjected to sham (vehicle) or SBR with vehicle or WUSTL0717 treatment (n = 3–4/group). (*D*) Volcano plot of the post-anastomosis ileum from RNA-seq showing differentially expressed genes (DEGs) between vehicle- and WUSTL0717-treated SBR groups. DEGs were defined as adjusted *P* value < .05 and log_2_ fold-change thresholds, with significant genes in *orange* (n = 4). (*E*) Transcript levels of LXR target genes in the duodenum, post-anastomosis ileum, and the liver from mice subjected to sham (vehicle) or SBR with vehicle or WUSTL0717 treatment (n = 4–9), analyzed by qRT-PCR. Statistical analysis was performed using 1-way analysis of variance (ANOVA) (*E*) or 2-way ANOVA (*C*) with Tukey’s honestly significant difference for multiple comparisons. Mean ± SEM; *P < .05, **P < .01, ***P < .001, ****P < .0001; ns, not significant. Mean ± SEM and *P* value thresholds are applied consistently across figures unless otherwise noted.

To investigate whether WUSTL0717 can activate intestinal LXR target genes in an SBS model, we used a mouse SBR model that induces liver fibrosis,^8,18^ wherein 75% of the proximal small intestine is resected (Figure 2A and Supplementary Figure 1A), while preserving the duodenum and the terminal ileum. WUSTL0717 (30 mg/kg, PO) or vehicle was administered daily to mice for 7 weeks, starting 3 weeks after SBR. This design allowed the mice to recover from the surgery before drug exposure (Figure 2B). To investigate WUSTL0717 efficacy, bulk RNA-seq was performed on the duodenum and the post-anastomotic ileum of the intestine as well as the liver following SBR. Gene set variation analysis of LXR target pathways identified from the Molecular Signatures Database (MSigDB) revealed increased LXR activity in the duodenum and ileum but not in the liver (Figure 2C and Supplementary Figure 1B). Key LXR target genes, including *Abca1*, *Srebf1*, and *Scd1* were upregulated in the RNA-seq dataset and confirmed by quantitative reverse transcription polymerase chain reaction (qRT-PCR) in both sexes after the 7-week treatment (Figure 2D and E, and Supplementary Figure 1C), with similar results observed after a shorter 10-day treatment (Supplementary Figure 1D). A heatmap of the top 50 genes altered in the duodenum after SBR showed marked downregulation of histone-encoding genes, a feature not rescued by WUSTL0717 (Supplementary Figure 2A). This may relate to chromosomal remodeling as the intestine adapts to a shortened absorptive surface. In the duodenum comparison of SBR vs SBR-WUSTL0717, messenger RNAs for antimicrobial peptides *Reg3g* and *Reg3b* were reduced (Supplementary Figure 2B), consistent with earlier evidence that bile acids and LXRs regulate antimicrobial peptides.^19^ Supporting the shift in regional identity, with the post-anastomotic ileum adopting features of the more absorptive duodenum, the transcription factor *Gata4*, a key regulator of jejunal and ileal identity,^20^ was downregulated after SBR and not restored by WUSTL0717 (Supplementary Figure 2C). In contrast, WUSTL0717 upregulated numerous LXR targets in the post-anastomotic ileum, while reducing expression of *Irf1*, a generator of the immune-modulatory metabolite itaconate in the tricarboxylic acid cycle,^21^ together with host defense genes *Irgm1* and *Irgm2* (Supplementary Figure 2D). Collectively, these results indicate that WUSTL0717 induces intestinal LXR targets, downregulates select host defense-associated genes, and spares activation of hepatic LXR targets.

### WUSTL0717 Favors Nutrient Acquisition and Metabolic Health Following Small Bowel Resection

**Figure 3.**
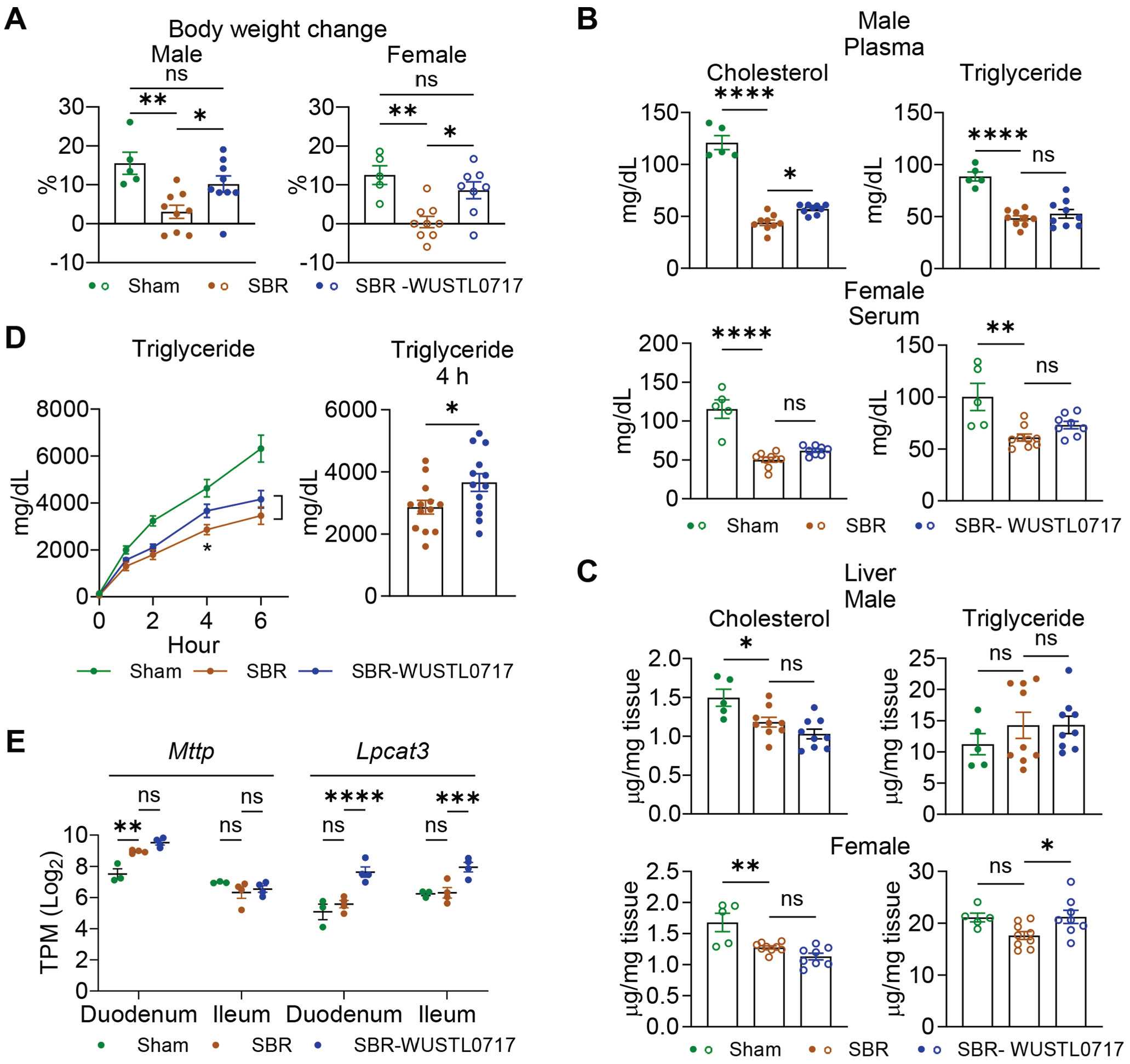
Rescue of body weight loss induced by SBR through intestinal-restricted LXR agonism. (*A–C, E*) WT male (*filled circles*) or female (*open circles*) mice underwent sham or SBR operation. Beginning 3 weeks post-surgery, the mice received either vehicle or WUSTL0717 (30 mg/kg, PO) daily for up to 7 weeks (n = 5–9/group). (*A*) Percentage change in body weight, calculated as (week 10 – week 2) post operation divided by the body weight at week 2. (*B*) Cholesterol and triglyceride levels from males (plasma) and females (serum). (*C*) Liver cholesterol and triglyceride levels. (*D*) Mice 10 to 11 weeks post operation, treated with vehicle or WUSTL0717 for 7 to 8 weeks, were used. Data from 2 independent experiments were combined. Plasma triglyceride levels were measured at 0, 1, 2, 4, and 6 hours after a 10 *μ*L/g olive oil gavage, preceded by a 500 mg/kg Tyloxapol intravenous injection (n = 12–14/group). (*E*) *Mttp* and *Lpcat3* transcript levels (TPM, transcripts per million) in the duodenum and the post-anastomosis ileum by RNA-seq (n = 3–4/group). Unpaired Student *t* test (*D*), 1-way analysis of variance (ANOVA) (*A–C*) or 2-way ANOVA (*E*) with Tukey’s honestly significant difference statistical test was applied.

Clinically, extensive SBR hinders weight gain and typically necessitates intravenous supplementation to maintain nutrition.^22,23^ Indeed, mice lost weight in the first 2 weeks after SBR and failed to regain it between 2 and 10 weeks (Figure 3A and Supplementary Figure 3A). Treatment with WUSTL0717 promoted ~10% gain in body weight between 2 and 10 weeks, similar to the normal growth rate in C57/BL6 mice over 8 weeks (Jackson Laboratory, Body Weight Information for Aged B6 [000664], https://www.jax.org) and similar to sham (Figure 3A and Supplementary Figure 3A). Both adiposity and activity decreased after SBR and remained unchanged with WUSTL0717 treatment (Supplementary Figure 3B and C). However, WUSTL0717 reversed the reduction in body temperature (Supplementary Figure 3D), consistent with increased metabolic heat production (Supplementary Figure 3E). Compared with sham, SBR diminished use of fatty acids as a fuel source, as evidenced by increased respiratory exchange ratio, which was not altered by WUSTL0717 (Supplementary Figure 3F). SBR profoundly reduced plasma cholesterol and triglycerides (Figure 3B). Blood triglyceride levels, as well as hepatic cholesterol and triglyceride levels, were not increased by WUSTL0717 in males or females, although in females, liver triglycerides were restored to sham levels by WUSTL0717, indicating that the gut-restricted LXR agonist did not promote hypertriglyceridemia or adverse lipogenesis in the liver (Figure 3B and C).

The top-ranked pathways upregulated after SBR in the post-anastomosis ileum involved triglyceride, fatty acid, and cholesterol metabolism, along with signaling pathways associated with peroxisome proliferator-activated receptors, CCAAT-enhancer-binding proteins, integrins, and epidermal growth factor, perhaps as compensation for reduced absorptive capacity (Supplementary Figure 3G). SBR itself also resulted in elevated *Pparg* (Supplementary Figure 3H), whereas genes highly expressed in the proximal region of the small intestine^24^ such as *Ppara*, *Apoc3*, and *Apoa4* were upregulated following WUSTL0717 treatment (Supplementary Figure 3H and I). *Nr1h3* encoding LXR*α* was elevated by WUSTL0717, suggesting an autoactivation loop (Supplementary Figure 3H). By contrast, *Nr1h2* encoding LXRß was unchanged by WUSTL0717 (Supplementary Figure 3H). Overall, Gene Set Enrichment Analysis pathways elevated by WUSTL0717 included peroxisome proliferator-activated receptor signaling, lipid and lipoprotein metabolism, pyruvate metabolism, lipid digestion mobilization, and transport (Supplementary Figure 3J). We thus assessed intestinal chylomicron secretion as a measure of intestinal lipid absorption and release into the circulation. SBR significantly reduced this output (Figure 3D), as previously published,^25^ but WUSTL0717 partially restored it (Figure 3D). By comparison, glucose uptake after glucose gavage was unaffected by WUSTL0717, supporting its specificity for affecting lipid and cholesterol metabolism (Supplementary Figure 3K). Although chylomicron synthesis genes like *Mttp* were not upregulated by WUSTL0717, WUSTL0717 increased intestinal *Lpcat3* (Figure 3E), an enzyme that improves lipid absorption by augmenting membrane permeability.^26^ Taking these data together, we conclude that WUSTL0717 positively affects metabolism, favorably remodeling the intestine in a manner that ameliorates metabolic deficits brought on by SBR.

### Metabolic Impact of WUSTL0717 Administered to Healthy Wild Type Mice

**Figure 4.**
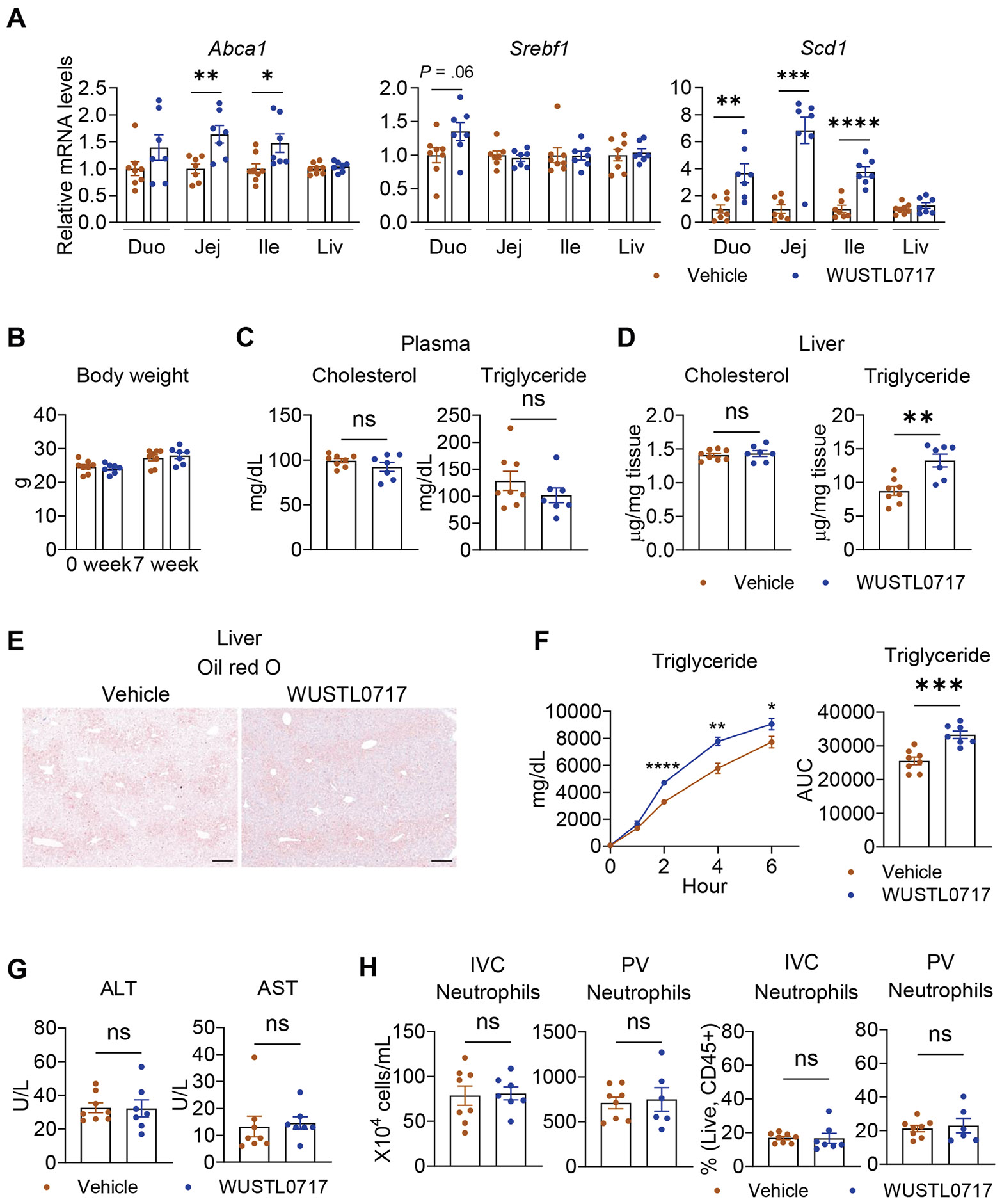
WUSTL0717 avoids metabolic dysfunction while promoting increased nutrient absorption. WT mice were treated with vehicle or WUSTL0717 (30 mg/kg, PO) daily for 7 weeks (n = 7–8/group). (*A*) Transcript levels of LXR target genes in the duodenum (Duo), jejunum (Jej), ileum (Ile), and liver (Liv) analyzed by qRT-PCR. (*B*) Body weight at baseline and after 7 weeks of WUSTL0717 treatment. (*C*) Plasma cholesterol and triglyceride levels. (*D*) Liver cholesterol and triglyceride levels. (*E*) Oil red O staining in the liver (*scale bar*, 200 *μ*m). The images shown are representative of images from 8 mice treated with vehicle, 7 mice treated with WUSTL0717. (*F*) Plasma triglyceride levels and area under the curve (AUC) in WUSTL0717-treated and vehicle groups, measured 9 days before euthanization at 0, 1, 2, 4, and 6 hours after a 10-*μ*L/kg olive oil gavage preceded by a 500-mg/kg intravenous Tyloxapol pretreatment. (*G*) Plasma ALT and AST activities. (*H*) Systemic (inferior vena cava [IVC]) and portal (PV) venous plasma neutrophils were measured by flow cytometry. Unpaired Student *t* test was applied for statistical evaluation.

The increase in chylomicron output by WUSTL0717 after SBR (Figure 3D) might relate to correction of SBR-linked impairments in lymphatic transport,^27^ unfavorable changes in the villus surface after SBR, or an independent impact on chylomicron synthesis and secretion. To investigate these alternatives and characterize metabolic effects under steady-state conditions, we next administered WUSTL0717 (30 mg/kg, PO) daily for 7 weeks to unoperated C57BL/6 WT mice. Representative LXR target genes were upregulated in the small intestine, but not in the liver (Figure 4A). Body weight, adiposity, food intake, activity, heat generation, and respiratory exchange ratio were comparable between vehicle- and WUSTL0717-treated mice (Figure 4B; Supplementary Figure 4A-E). Blood cholesterol and triglyceride levels as well as hepatic cholesterol levels, were unchanged, whereas liver triglyceride levels showed a modest WUSTL0717-induced increase within physiological range (Figure 4C and D).^28^ Oil Red O staining was comparable between groups, indicating minimal lipid accumulation in either the liver or small intestine following WUSTL0717 treatment (Figure 4E and Supplementary Figure 4F). Intestinal triglyceride absorption, reflected by chylomicron secretion, was indeed elevated by WUSTL0717 (Figure 4F). Liver enzymes ALT and AST levels remained normal in plasma, indicating that WUSTL0717 did not promote liver damage (Figure 4G). Furthermore, whereas systemic LXR agonists previously caused adverse neutrophil reductions in mice and humans,^29^ gut-restricted WUSTL0717 did not alter neutrophil counts or percentages among leukocytes in systemic (inferior vena cava) or portal blood (Figure 4H and Supplementary Figure 4G). These data indicate that long-term WUSTL0717 treatment in healthy mice minimally affects metabolism, lipid status, or leukocyte homeostasis. It modestly elevates chylomicron secretion, mirroring our observations following SBR, reflecting a direct drug effect on lipid absorption rather than adaptation or lymphatic changes specific to SBS.

### WUSTL0717 Protects the Liver From Fibrotic Injury After Small Bowel Resection

**Figure 5.**
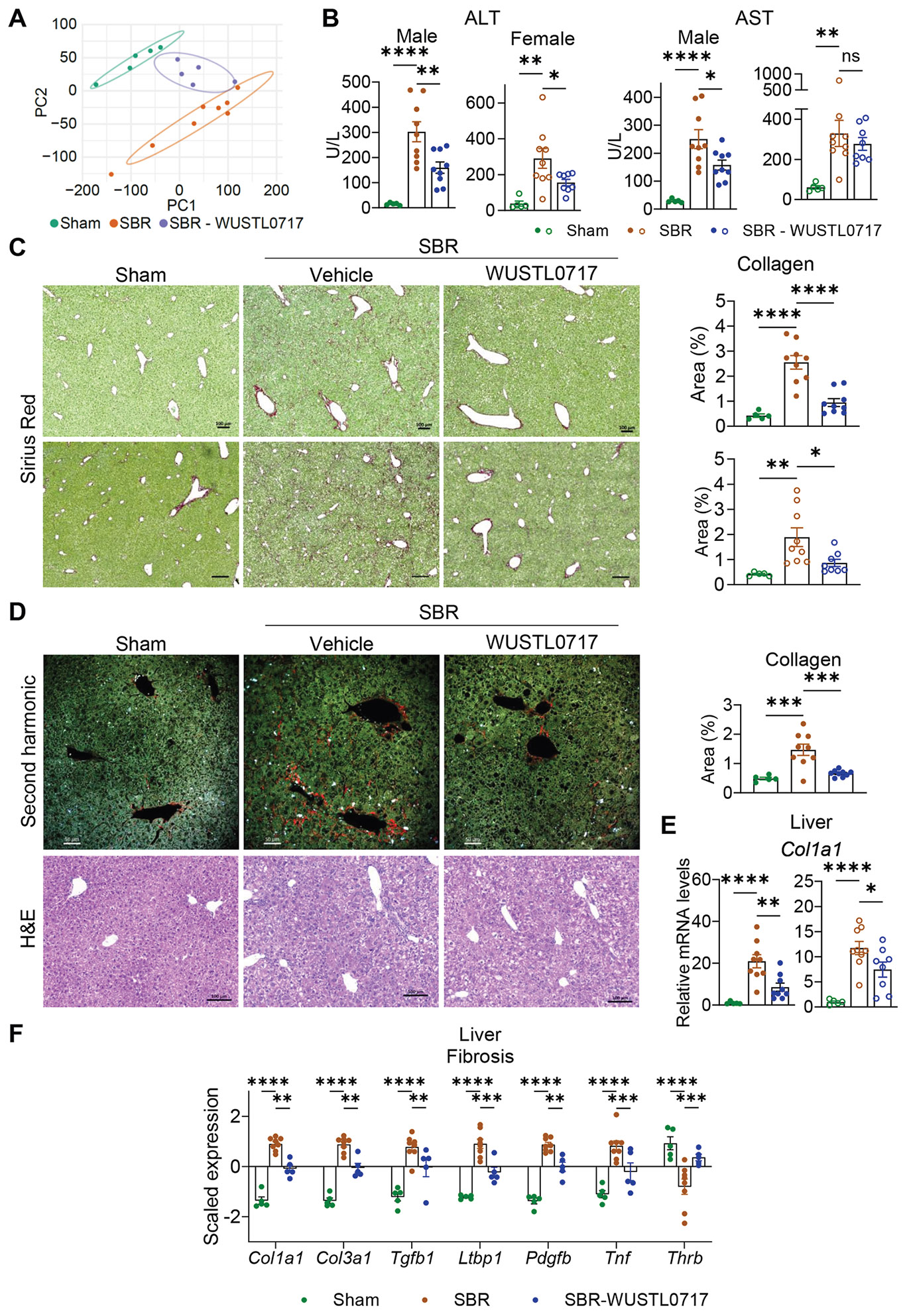
Protection against SBS-induced liver fibrosis through intestine-restricted LXR activation. WT male (*filled circles*) or female (*open circles*) mice underwent sham or SBR operation. Beginning 3 weeks post-surgery, the mice received either vehicle or WUSTL0717 (30 mg/kg, PO) daily for 7 weeks and were then euthanized for analysis (n = 5–9/group). (*A*) Principal component analysis of liver transcriptomes from the indicated groups. (*B*) ALT and AST from males (plasma) and females (serum). (*C*) Sirius Red–stained liver sections (upper: male; lower: female) with quantification from 5 areas per mouse, plotted as *individual dots* (*scale bar*, 100 *μ*m). (*D*) Second-harmonic generation (SHG)-based collagen detection with quantification from 6 to 12 different areas per mouse, shown as *individual dots*, and representative hematoxylin-eosin (H&E)–stained liver sections. Scale bars: 50 *μ*m (SHG), 100 *μ*m (H&E). (*E*) Col1a1 transcript levels in the liver analyzed by qRT-PCR. (*F*) Differentially expressed genes associated with collagen accumulation, hepatic stellate cell activation, and cytokine-related pathways in the liver. Statistical evaluations used 1-way analysis of variance (ANOVA) (*B, E*; Dunnett’s test), 1-way ANOVA (C and D; Tukey’s honestly significant difference [HSD]), and 2-way ANOVA (*F*; Tukey’s HSD).

To examine the intestine-restricted effects of WUSTL0717 on liver fibrosis, we performed RNA-seq on liver samples from sham- or SBR-operated mice. Principal component analysis revealed distinct clustering of SBR and sham-operated groups. WUSTL0717 diminished this segregation, shifting profiles toward the sham cohort (Figure 5A). Blood ALT and AST activities indicated reduced liver damage in the WUSTL0717-treated male mice, with a similar ALT reduction in females (Figure 5B). Collagen accumulation in the liver following SBR was reversed by WUSTL0717, as shown by Sirius Red staining and second-harmonic generation imaging (Figure 5C and D). Elevated *Col1a1* transcripts (Supplementary Materials and Methods, Supplementary Table 4), a key contributor to liver fibrosis, were also reduced (Figure 5E). Similar reductions in Sirius Red staining and *Col1a1* expression were observed in females (Figure 5C and E). Differentially expressed genes revealed that fibrosis-associated genes were significantly downregulated in the liver following WUSTL0717 treatment after SBR, including those for collagen accumulation (*Col1a1*, *Col3a1*), stellate activation and extracellular matrix remodeling (*Tgfb1*, *Ltbp1*, *Pdgfb*), and inflammatory cytokines (*Tnf*) (Figure 5F and Supplementary Figure 5A). Moreover, thyroid receptor *ß* (*Thrb*), downregulated after SBR, was restored by WUSTL0717 treatment (Figure 5F), highlighting its therapeutic potential, especially as a thyroid hormone receptor beta agonist was recently approved by the Food and Drug Administration for metabolic dysfunction–associated steatohepatitis with fibrosis.^30^ Genes associated with cholestasis were also affected. SBR increased *Krt7* (Keratin 7)^31^ while decreasing *Cyp8b1*, and these changes were reversed by WUSTL0717, which also upregulated *Abcb11*, suggesting a restoration of normal bile secretion (Supplementary Figure 5A and B). Antioxidant genes were upregulated by WUSTL0717 (Supplementary Figure 5C). Gene set variation analysis confirmed that collagen accumulation and extracellular matrix remodeling pathways, significantly upregulated after SBR, were downregulated by WUSTL0717 (Supplementary Figure 5D). The top-ranked downregulated liver genes after SBR were often restored in the presence of WUSTL0717 and included numerous major urinary protein family members (Supplementary Figure 5E), whose downregulation is implicated in metabolic liver disease.^32^ The top genes upregulated by SBR—including collagen genes and *Spp1* (*Osteopontin*), often produced by macrophages and associated with fibrosis^33^—were decreased by WUSTL0717 (Supplementary Figure 5E). These data demonstrate that WUSTL0717 acts in the intestine and robustly reduces liver fibrosis after SBR.

### Phospholipids in the Portal Vein Correlate With the Protective Effects of the Gut-Restricted Liver X Receptor Agonist WUSTL0717 in Short Bowel Syndrome

**Figure 6.**
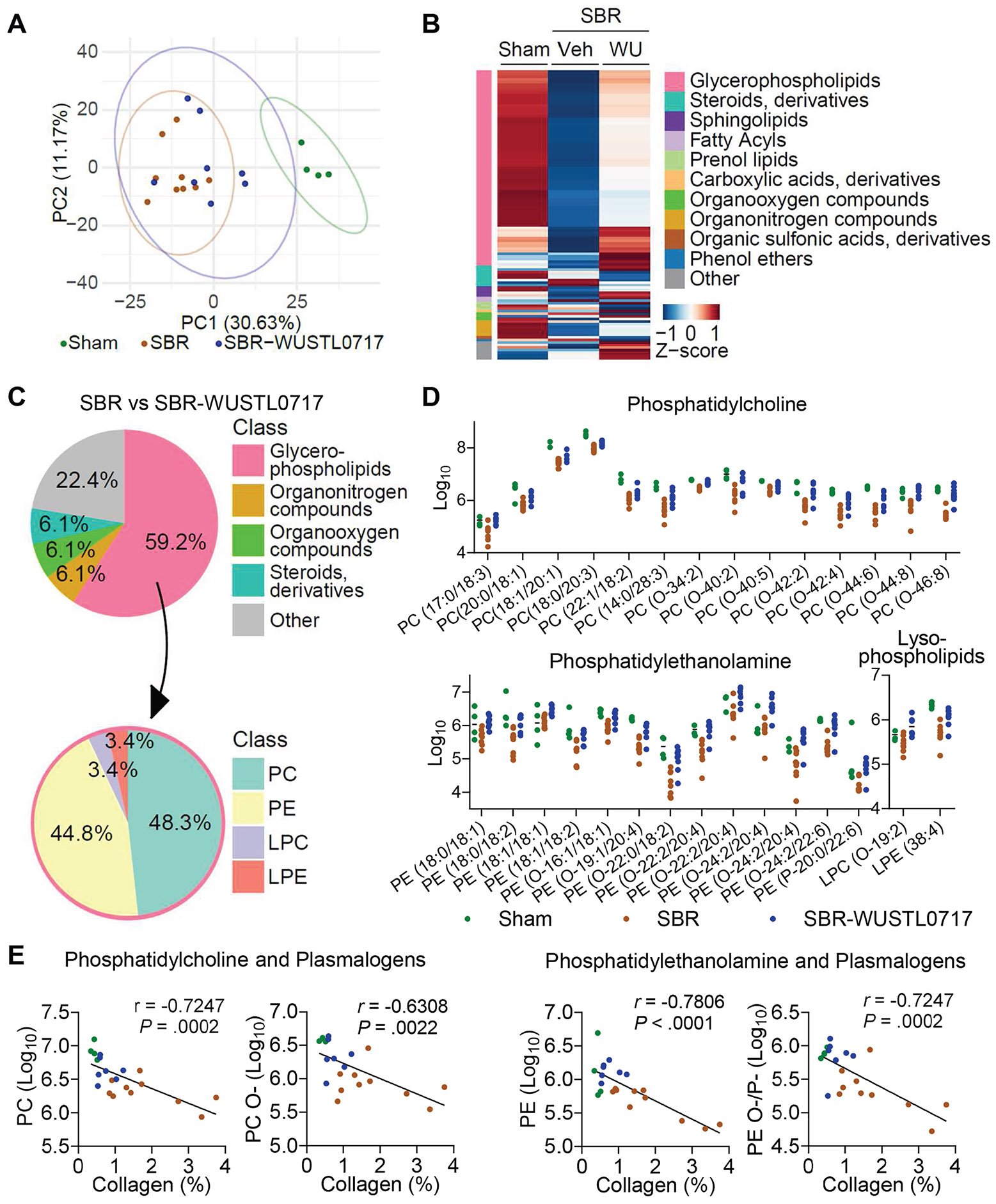
Association of portal venous phospholipids with the hepatoprotective effects of the gut-restricted LXR agonist after SBR. Lipid and metabolite profiling of portal venous serum using LC-MS/MS in WT female mice with sham or SBR surgery. Three weeks after surgery, mice received vehicle or WUSTL0717 for 7 weeks (n = 4–9/group). (*A*) Principal component analysis of overall metabolites. (*B*) Heatmap of metabolites with significant differences between SBR vs SBR-WUSTL0717 and sham vs SBR. (*C–E*) Analysis of portal venous serum metabolites significant at false discovery rate < 0.1 and fold change > 1.5, focusing on glycerophospholipids and their association with liver fibrosis. (*C*) Pie charts of metabolite classes (*left*) and the sub-classes of glycerophospholipids (*right*). (*D*) Individual glycerophospholipid metabolites from the sub-classes in (*C*) (*right*). PE (O-22:2/20:4) and PE (O-24:2/20:4) are each represented twice as isomeric species. (*E*) Correlation of portal venous serum PC, PC O-, PE, PE O-, and plasmenyl PE (PE P-) with liver collagen area (Figure 5C, lower). Each dot represents a matched individual from (*D*). Pearson correlation.

We earlier concluded that intestinal HDL that travels in the portal vein is central to protecting the liver from IFALD.^8^ This past work focused on eliminating intestinal epithelial cell expression of ABCA1,^8^ a key transporter of cholesterol and phospholipids packaged by HDL. Indeed, phospholipids are largely dependent on lipoproteins for transport in plasma and HDL is the dominant lipoprotein in mice.^14,15,34^ When we herein carried out lipidomic and metabolite profiling of the portal vein samples in our experimental cohorts, a clear distinction was revealed between sham and SBR groups, as demonstrated by principal component analysis (Figure 6A). SBR led to a marked reduction in numerous lipid classes, while a few were elevated (Figure 6B). Treatment with WUSTL0717 produced an overall modest shift (Figure 6A). Closer examination revealed that glycerophospholipids were significantly decreased in SBR and partially restored or increased to sham levels with WUSTL0717 treatment (Figure 6B). To further characterize treatment-specific changes, we performed statistical filtering based on log_2_ fold-change and adjusted *P* value between the SBR and SBR-WUSTL0717 groups, identifying 49 significantly altered metabolites. Glycerophospholipids again emerged as the predominant class, as illustrated in the pie chart, with PC and phosphatidylethanolamine (PE) being the most represented sub-classes within this group (Figure 6C and D). Correlation analysis within this subset revealed that both PC and ether-linked PC (PC O-), as well as PE and ether-linked PE (PE O-), were each inversely correlated with liver collagen accumulation (Figure 6E). Thus, WUSTL0717 increases several phospholipids in the portal vein, an outcome possibly linked to the protective effects of WUSTL0717 against liver fibrosis after SBR.

### Intestinal Apoa1 and High-density Lipoprotein Cholesterol Protect Against Liver Injury Following Small Bowel Resection

**Figure 7.**
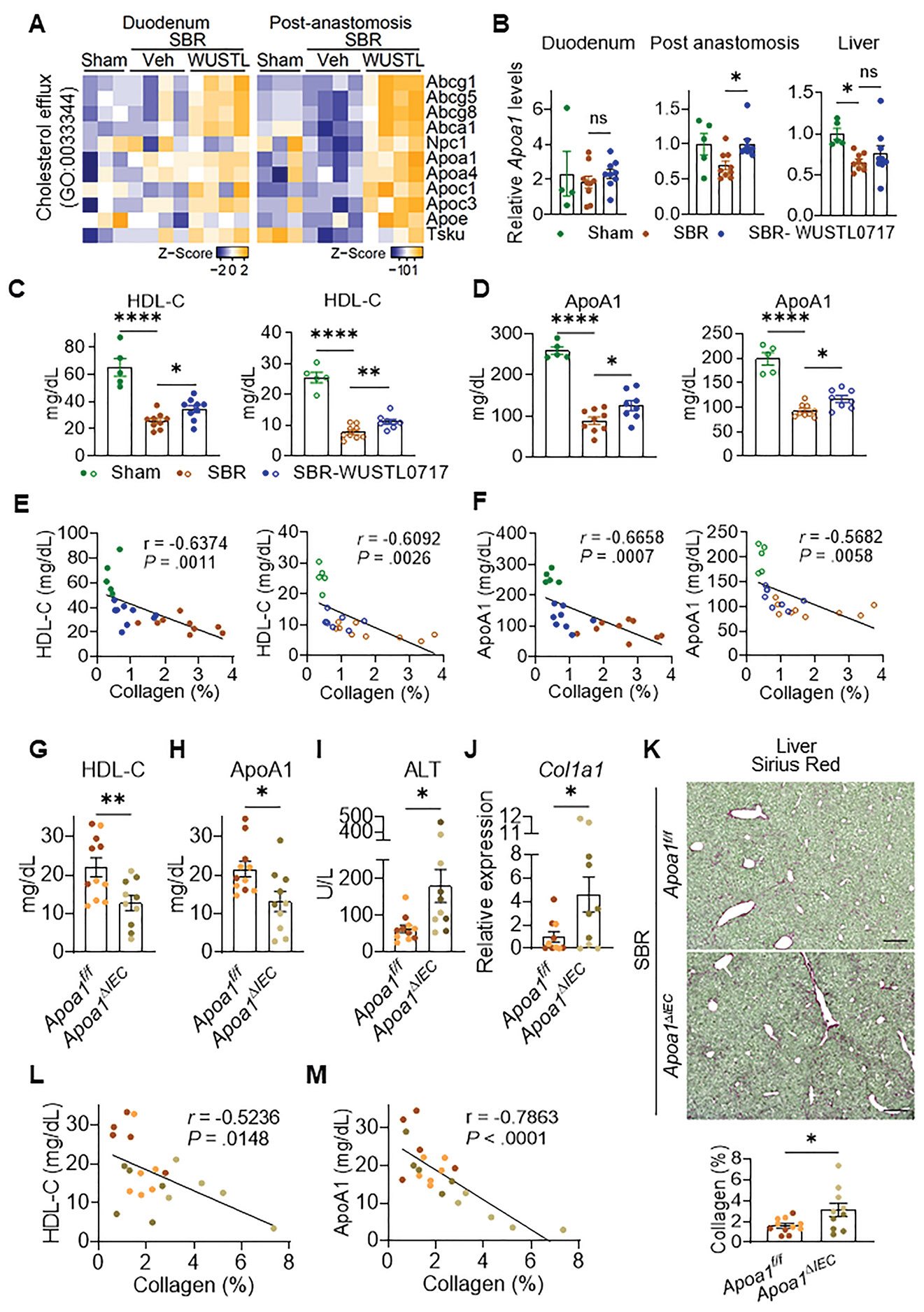
Aggravated IFALD following the deficiency of intestinal ApoA1 and HDL-C after SBR. (*A–D*) WT male (*filled circles*) or female (*open circles*) mice underwent sham or SBR. Starting 3 weeks later, mice received daily vehicle or WUSTL0717 (30 mg/kg, PO) for 7 weeks, followed by analysis (n = 5–9/group). (*A*) Heatmap of cholesterol efflux–related transcripts in the duodenum and post-anastomosis ileum from male mice (n = 3–4/group). (*B*) qRT-PCR analysis of *Apoa1* transcripts in the duodenum, post-anastomosis ileum, and liver (n = 4–9/group). (*C* and *D*) Portal venous HDL-C (*C*) and ApoA1 (*D*) levels from males (plasma) and females (serum) (n = 5–9/group). (*E* and *F*) Correlation of portal venous plasma HDL-C and ApoA1 levels with liver collagen area (Figure 5C, *upper*). Each *dot* represents a matched individual from (*C*) and (*D*). (*G–M*) *Apoa1^ΔIEC^* mice underwent SBR and were euthanized 11 weeks later. *Dark dots* represent males and *light dots* represent females (males, n = 7/group; females: n = 6–7/group). (*G* and *H*) Portal venous serum HDL-C and ApoA1 levels. (*I*) Systemic (inferior vena cava) serum ALT levels. (*J*) Liver *Col1a1* transcript levels analyzed by qRT-PCR. (*K*) Sirius Red–stained liver sections (*scale bar*, 200 *μ*m) with quantification from 5 to 6 areas per liver, 1 image per mouse (males, n = 7/group; females: n = 6–7/group). (*L* and *M*) Correlation of portal venous serum HDL-C and ApoA1 levels with liver collagen area. Each *dot* represents a matched individual from (*K*)–(*M*). Statistical evaluations were done using unpaired Student *t* test (*C*, *G–K*), 1-way analysis of variance with Tukey’s honestly significant difference (*B*, *D*), or Pearson correlation (*E*, *F*, *L*, and *M*).

Because HDL would be expected to be the major carrier of phospholipids in mouse plasma,^15^ we hypothesized that intestine-derived ApoA1 itself would be important for hepatic protection, as ApoA1 and phospholipids together support HDL particle integrity.^34^ In mice receiving SBR, WUSTL0717 administration enriched the cholesterol efflux gene ontology in both the post-anastomosis ileum and the duodenum (Figure 7A), but *Apoa1* transcript levels increased only in the post-anastomosis ileum (Figure 7B), underscoring the region-specific impact of WUSTL0717 on intestinal gene expression. After WUSTL0717 administration, portal venous HDL-C levels and ApoA1 levels were elevated in both sexes (Figure 7C and D). In sham-operated mice, systemic HDL-C exceeded portal venous HDL-C, consistent with previous reports,^8^ but SBR reversed this relationship, lowering systemic HDL-C below portal venous levels (Figure 7C and Supplementary Figure 6A). WUSTL0717 treatment elevated HDL-C in both the portal and systemic veins (Figure 7C and Supplementary Figure 6A), partially reversing the suppression caused by SBR. Portal venous HDL-C and ApoA1 levels were inversely correlated with liver collagen accumulation (Figure 7E and F). ApoA1 was also detected in the ileal mucus and increased after WUSTL0717 in male mice (Supplementary Figure 6B and C). Given its presence in the mucus and the concept that HDL in the mucus might act as an antimicrobial agent or microbiome modifier,^35^ we examined whether mucosal ApoA1 levels were associated with liver fibrosis; however, no correlation was observed (Supplementary Figure 6D and E). Fecal 16S ribosomal RNA sequencing revealed that the microbiome was stable before surgery but diverged across groups 10 weeks after surgery (Supplementary Figure 7A-C). Alpha diversity index increased after SBR compared with sham, and WUSTL0717 did not alter this increase, whereas WUSTL0717 treatment shifted beta diversity between the sham and SBR groups (Supplementary Figure 7A and B). Notably, the abundance of *Akkermansia*—a bacterium known to ameliorate metabolic dysfunction–associated fatty liver disease and liver fibrosis^36^—was markedly reduced by SBR but restored with WUSTL0717 treatment (Supplementary Figure 7C-E). However, neither liver collagen area nor portal venous ApoA1 levels correlated with *Akkermansia* abundance (Supplementary Figure 7F). These data suggest that the impact of WUSTL0717, through alterations in portal venous contents, is more pertinent to hepatic protection than changes in the microbiome or mucus.

To test the hypothesis that intestinal ApoA1 is required for liver protection, SBR was performed on mice lacking *Apoa1* in intestinal epithelial cells (*Apoa1^ΔIEC^*). In these mice, portal venous HDL-C levels were further reduced compared with the already low levels in WT SBR mice (Figure 7G), as was portal venous ApoA1 (Figure 7H). In contrast, serum ALT levels were elevated (Figure 7I), and loss of intestinal ApoA1 also increased markers of liver fibrosis, including type I collagen messenger RNA (Figure 7J) and Sirius Red staining (Figure 7K). The extent of fibrotic area was inversely correlated with portal venous HDL-C and ApoA1 (Figure 7L and M). We conclude that portal venous HDL-C and ApoA1 protect the liver from injury after SBR, and that the elevation of HDL in the gut-liver axis achieved by WUSTL0717 treatment mitigates IFALD.

## Discussion

Building on our previous work linking intestine-derived HDL to liver injury via the portal vein,^8,37^ we explored the therapeutic potential of LXR agonists with activity confined to the intestine. Although systemic LXR agonists failed clinically due to hepatic lipogenesis,^29^ the sole report of GW6340 as a putative gut-restricted LXR agonist^13^ seemed a promising alternative, but the report lacked pharmacokinetic evaluation, preventing rigorous evaluation of its intestinal restriction and functional impact. To address the lack of information on gut-restricted LXR agonists, we synthesized GW6340, described in the patent PCT/US01/27622, and named it WUSTL0717. Pharmacokinetic and molecular analyses confirmed its intestinal restriction, showing LXR targets induction in the intestine but not in the liver. The amide structure of WUSTL0717 is central to its gut-restricted activity. Compared with the carboxylic acid of GW3965^9^—the sole structural difference between the 2 compounds—the amide remains largely nonionized at intestinal pH and is less polar and more metabolically stable, collectively limiting systemic uptake and promoting gut luminal retention. In addition, the high molecular weight (581.078 g/mol), 13 rotatable bonds, and low kinetic solubility (0.29 mM) of WUSTL0717 fall outside the optimal range associated with high oral bioavailability.^38^ Because WUSTL0717 must enter intestinal epithelial cells to activate LXR, it could theoretically exit these cells to mobilize systemically via lymphatics or blood,^39^ but our pharmacokinetic studies detected minimal trafficking from the intestine. It seems likely that, in addition to its nonpolar character, robust catabolism may also contribute to its confinement to the intestine.

WUSTL0717 enhances transcription of lipid metabolic genes and improves chylomicron secretion, conferring metabolic benefit by fostering weight regain after SBR, likely via proximal intestinal action. In unoperated mice, WUSTL0717 modestly increased chylomicron secretion, without obvious negative metabolic effects, suggesting neutrality in health but clear benefits after SBR. This adaptive effect may relate to a recently reported role of LXR signaling in promoting intestinal stem cell–driven regeneration and tissue adaptation after damage.^40^ That LXR agonists can promote intestinal epithelial health without promoting tumorigenesis bodes well for translational potential of targeting LXR in a gut-restricted manner. Indeed, intestinal remodeling capacity is considered critical in the context of SBS, as impaired adaptation contributes to nutritional deficiency and hepatic complications.^1^ IFALD from SBS can be severe, sometimes necessitating a liver transplant, yet no standard therapy exists. We propose that the application of gut-restricted LXR agonists may be particularly valuable following substantial resection, creating a window for preventive intervention. In our model, WUSTL0717 protected the liver by attenuating fibrosis, inflammation, and cholestasis while restoring bile secretion, antioxidant pathways, and *Thrb* expression. Notably, a thyroid hormone receptor beta agonist was recently approved by the Food and Drug Administration for liver fibrosis.^30^ WUSTL0717 also modestly altered some host defense–related genes, with the functional significance of these changes requiring further study.

Our findings suggest that the phospholipids restored by WUSTL0717 likely originate from the intestine^41^ and reflect elevated portal venous ApoA1 and HDL that, in turn, support hepatoprotection. Higher portal phospholipid levels correlated with reduced fibrosis, supported by studies of phosphatidylcholine supplementation reducing liver fibrosis.^42^ However, whether these phospholipids themselves protect the liver via HDL delivery, by altering cargo-affiliated HDL, or directly affect hepatic function remains unknown. We show that expression of ApoA1 by the intestine is, like enterocyte-derived ABCA1,^8^ important in quelling IFALD. Although ApoA1, the protein backbone of HDL, was elevated in ileal mucus^35^ by WUSTL0717, its presence in mucus did not correlate with liver fibrosis. Similarly, the fecal microbiome, comparable across the mice before surgery, diverged markedly by 10 weeks. *Akkermansia muciniphila*, a microbe considered beneficial,^36^ was reduced after SBR but restored with WUSTL0717 treatment. However, these changes did not correlate with liver fibrosis outcomes, suggesting a secondary rather than a primary protective mechanism. In contrast, portal ApoA1 and HDL strongly correlated inversely with fibrosis, and intestinal *Apoa1* deletion worsened liver outcomes, underscoring that gut-derived HDL protects the liver primarily through the portal circulation. In conclusion, our characterization of WUSTL0717 as a gut-restricted LXR agonist that modulates portal HDL biogenesis and lipid handling supports future evaluation of WUSTL0717 in large animal models to translationally assess whether gut-restricted LXR agonists may be therapeutically viable to reduce intestine-driven liver disease.

## Supplementary Material

1

2

Note: To access the supplementary material accompanying this article, visit the online version of *Gastroenterology* at www.gastrojournal.org, and at https://doi.org/10.1053/j.gastro.20110.1053/j.gastro.2025.12.015.

## Data Availability

The sequencing and expression data have been deposited in the Gene Expression Omnibus of the National Center for Biotechnology Information under accession code GSE287046. On publication, all data will be accessible through the Open Science Framework. The corresponding author also may be contacted as needed to clarify any concerns around data or resource access.

## WHAT YOU NEED TO KNOW

### BACKGROUND AND CONTEXT

Liver X receptor activation in the small intestine protects against liver failure after extensive small bowel resection, but only gut-restricted liver X receptor agonists would be translationally feasible. Yet, a gut-restricted liver X receptor agonist has never been characterized.

### NEW FINDINGS

Here, we synthesize and study the pharmacokinetics of a gut-restricted liver X receptor WUSTL0717, showing its strong liver X receptor activation restricted to the intestine. WUSTL0717 augments metabolic recovery after bowel resection in mice, protecting the liver against fibrosis in a manner that relies on enterocyte ApoA1, the core protein of high-density lipoprotein, and raises portal venous phospholipids.

### LIMITATIONS

Future studies are needed to evaluate the potential of a gut-restricted liver X receptor agonist to protect the liver after gut resection without adverse effects in humans.

### CLINICAL RESEARCH RELEVANCE

Therapies for intestinal failure–associated liver disease are needed. There has long been interest in action of liver X receptor limited to the intestine. This is the first full demonstration of a gut-restricted liver X receptor agonist.

### BASIC RESEARCH RELEVANCE

We show that WUSTL0717 preserves some phospholipid transport from the intestine through the portal vein that is diminished after small bowel resection. This is likely due to phospholipid transport by high-density lipoprotein and, accordingly, we show that enterocyte-derived ApoA1 protects the liver against inflammatory injury, underscoring major physiological roles of high-density lipoprotein beyond cardiovascular health. We considered the possibility that high-density lipoprotein in the intestinal mucus controls the microbiome to protect the liver but found that its presence in the portal vein was most critical.

## Abbreviations used in this paper:

ALT: alanine aminotransferase
ApoA1: Apolipoprotein A1
AST: aspartate aminotransferase
HDL: high-density lipoprotein
HDL-C: high-density lipoprotein cholesterol
IFALD: intestinal failure–associated liver disease
LC-MS/MS: liquid chromatography–tandem mass spectrometry
LXR: liver X receptor
PC: phosphatidylcholine
PC O-: ether-linked PC
PE: phosphatidylethanolamine
PE O-: ether-linked PE
qRT-PCR: quantitative reverse transcription polymerase chain reaction
PO: per os
RNA-seq: RNA sequencing
SBR: small bowel resection
SBS: short bowel syndrome
WT: wild type
