# Supplementary material for: A Gut-Restricted Liver X Receptor Agonist Ameliorates Liver Injury in Experimental Short Bowel Syndrome
Source: Gastroenterology. Author manuscript; Available in PMC 2026 May 11. (PMC13160593; doi:10.1053/j.gastro.2025.12.015)
Supplement: 1 [file NIHMS2168396-supplement-1.pdf]

Supplementary Materials and Methods

Molecular modeling

WUSTL0717 and GW3965 were modeled in LXRα protein from reference structure PDBID: 3IPU and based on the coordinates of GW3965 in Xray structure 1PQ6.[1, 2] WUSTL0717 was modeled in LXRβ based on the coordinates of GW3965 in Xray structure 1PQ6. Charges were modeled using AM1BCC, energy minimization using Amber ff14SB, Steepest descent,[3] and conjugate gradient algorithms[4] implemented in UCSF Chimera.[5]

Ligand binding in LanthaScreen TR-FRET LXRβ coactivator assays

Binding assays of graded concentrations of WUSTL0717 or GW3965 to LXRβ were performed using the LanthaScreen TR-FRET LXRβ Coactivator kit (Thermo Fisher Scientific) following the manufacturer's protocol. Briefly, after the addition of 5 nM LXRβ-LBD to the compounds, a mixture of peptide (100 nM, Fluorescein-D22 for LXRβ) and antibody (10 nM, Tb-anti-GST) was added to the reaction followed by incubation in room temperature for 4 hours. TR-FRET ratio was calculated by dividing the emission at 520 nm by the emission at 495 nm using either Synergy 2 (BioTek) or FlexStation 3 (Molecular Devices) plate reader. The TR-FRET ratio was then normalized by dividing the ratio of each concentration by the averaged ratio of the positive control, which was set equal to 100%. Concentration-response curves were fitted using 4-variable non-linear regression.

Activation of transcriptional functions of LXRα and LXRβ

Plasmids LXR-α (79514; Addgene), LXR-β (79513; Addgene), and LXRE_Luc (177622; Addgene) were transfected into HEK293T cells (ATCC CRL-3216) using the Lipofectamine™ 3000 kit (L3000001; Thermo Fisher Scientific). 24 hours post-transfection, the cells were plated in a 96-well plate at a density of 30,000 cells per well. After an additional 24 hours, the cells were treated with WUSTL0717 or GW3965 for another 24 hours. Luciferase activity was then measured using a Synergy HTX (BioTek) plate reader with the ONE-Glo™ luciferase assay kit (E8130; Promega) at room temperature. Concentration-response curves were fitted using 4-variable non-linear regression.

Kinetic Solubility

Compound from a 10 mM DMSO stock solution was introduced to pre-warmed pH 7.4 phosphate buffered saline in a 96-well plate with a final DMSO concentration of 1%. The plate was maintained at 37°C for 24 hours on an orbital shaker and centrifuged through a Millipore Multiscreen Solvinter 0.45 micron low binding PTFE hydrophilic filter plate and analyzed by HPLC. Peak area was compared to standards of known concentration.

Plasma protein binding

Plasma protein binding was determined using equilibrium dialysis. All samples were tested in triplicate using the RED Rapid Equilibrium Dialysis Device (Thermo Fisher Scientific). The initial drug concentration in the plasma chamber was 2 µM, and phosphate buffered saline was added to the receiver chamber. The plate was covered and allowed to shake in a 37ºC incubator for 6 hours. 25 µl was sampled from the plasma and PBS chambers, which were then diluted with either blank PBS or plasma to achieve a 1:1 ratio or plasma:PBS for all samples. The concentration of the drug in the plasma and PBS chambers was determined by LC-MS/MS. The fraction bound was calculated as ([plasma] – [PBS]) / [plasma].

Hepatic microsomal stability

Microsome stability was evaluated by incubating 1 µM test compound with 1 mg/mL hepatic microsomes in 100 mM KPi, pH 7.4. The reaction was initiated by adding NADPH (1 mM final concentration). Aliquots were removed at 0, 5, 10, 20, 40, and 60 minutes and added to acetonitrile (5X, v:v) to stop the reaction and precipitate the protein. NADPH dependence of the reaction was evaluated by setting up incubations without NADPH. At the end of the assay, the samples were centrifuged through a Millipore Multiscreen Solvinter 0.45 micron low binding PTFE hydrophilic filter plate and analyzed by LC-MS/MS. Data were log-transformed and represented as half-life. The microsomes were purchased from B1oIVT. Human were av200 donor pool/mixed sex.

Metabolic cages

Mice were weighed, and their core body temperature was measured using a rectal thermometer. Body composition was assessed using EchoMRI-100H 2n1 (EchoMRI). Afterwards, the mice were housed individually in Comprehensive Laboratory Animal Monitoring System (CLAMS) metabolic cages (Columbus Instruments) for one day of acclimation, followed by 24 hours for recording. The cages were contained within a light-sealed environmental enclosure with LED white light strips next to each cage, which were turned on at 6:00am (Zeitbeber Time 0) and turned off at 6:00pm (Zeitgeber Time 12) each day for a 12h:12h light:dark cycle that matches that of the standard animal facility. The enclosure temperature was set a 22.2 °C. The airflow rate was set at 0.90 L/min, with an 18-second bleed of the air sampling tube followed by a 2-second measurement period for each cage, with sampling of enclosure air in each interval. Energy expenditure (heat) and RER were calculated from measurements of the volume of oxygen consumption and carbon dioxide production using indirect calorimetry with a zirconia O_2 sensor and CO_2 sensors. Ambulatory activity was measured using infrared laser detector arrays positioned along the X- and Y-axes at animal height.

qRT-PCR assays

Total RNA from tissues was extracted using Direct-zol RNA Miniprep Kits (R2052; Zymo Research) and was reverse-transcribed into cDNA using the High-Capacity cDNA Reverse Transcription Kit (4368814; Thermo Fisher Scientific). cDNA was amplified either TaqMan Fast Advanced Master Mix (4444554; Thermo Fisher Scientific) with the following probes: *Abca1* (Mm00442646_m1), *Srebf1* (Mm00550338_m1), *Scd1* (Mm00772290_m1), *Apoa1* (Mm00437569_m1), *Actb* (Mm02619580_g1), or *18S* (Hs99999901_s1), or SYBR Green qRT-PCR Master Mix (A46110; Thermo Fisher) with primer sequences listed in Supplementary Table 4. qRT-PCR was conducted using QuantStudio 6 Pro (Applied Biosystems).

Preparation of ileal mucosal protein for ApoA1 ELISA

Mucus collection was performed as previously described[6] with minor modifications. After flushing the small intestine with PBS, a 2-cm segment of ileum was dissected, opened lengthwise and pinned flat. Any remaining luminal contents were gently flushed with additional PBS rinses. The mucosal surface was subsequently rinsed with 2 mL of 10 mM Tris-HCl (pH 7.4) containing 5 mM $CaCl_2$, followed by gentle scraping using a cell lifter (08-100-240; Fisher Scientific). The collected mucus was transferred into 500 μL of lysis buffer [50 mM Tris-HCl (pH 7.5), 100 mM NaCl, 5 mM EDTA, 1% Triton X-100, and protease inhibitor]. The lysate was centrifuged twice at 13,000 rpm for 10 min to remove debris, and the final supernatant was stored at −20°C. Total protein was quantified by the Bradford assay, and 1:15 diluted samples were analyzed for ApoA1 levels using an ELISA kit (3750-1HP; Mabtech).

Intestinal lipid absorption and chylomicron secretion assay

Mice that were 9 to 11 weeks post-SBR with 6 to 8 weeks of WUSTL0717 administration, as well as non-operated mice that had received 6 weeks of WUSTL0717 treatment, underwent an intestinal triglyceride absorption assay. Mice were fasted in the dark cycle for 8–11 hours, then injected *i.v.* with 500 mg/kg body weight of Tyloxapol (T0307; Sigma-Aldrich) and then 10 µl/g body weight of olive oil (O1514; Sigma-Aldrich) via oral gavage one hour later. Blood was collected from tail veins at baseline and at 1, 2, 4, and 6 hours. Plasma triglycerides were measured using the L-Type TG-H kit (Wako Chemicals) read using a Cytation 5 Cell Imaging Multi-Mode Reader (BioTek).

Glucose uptake assay

Mice were 13 weeks post-SBR and had received 10 weeks of WUSTL0717 treatment before being fasted overnight for 15 hours. They were then administered a glucose solution (G8270; Sigma-Aldrich) via oral gavage at a dose of 2 g/kg body weight. Blood glucose levels were determined from the tail veins at baseline and 15, 30, 60, and 120 minutes after glucose injection using GLUCOCARD Vital (760001; Arkray).

Flow cytometry

Cells from blood stained with acridine orange were counted using an automated cell counter (Cellometer Auto T4; Nexelcom Bioscience). Red blood cells were lysed using BD Pharm Lyse (555899; BD Biosciences) for 8 minutes at room temperature, followed by centrifugation at 500 × g for 5 minutes. The lysis step was repeated 2–3 times as needed. The remaining cells were resuspended in PBS for 30 minutes. The cells were then stained with Live Zombie NiR (423106; Biolegends) in PBS for an additional 30 minutes to exclude dead cells. Next, the cells were stained with antibodies against cell surface markers for 30 minutes in FACs buffer consists of 2% FBS, 2 mM EDTA, 0.02% sodium azide, and 20% Brilliant buffer (563794; BD Biosciences) in PBS. Conjugated antibodies against CD45 (568336) and CD11b (612801) were sourced from BD Biosciences, and Ly6G to identify neutrophils was sourced from BioLegend (127641). Cells were acquired using the 5-lasers, 64-channels Cytek Aurora spectral flow cytometer (Cytek Biosciences) equipped with SpectroFlo software (version 3.1.0) and analyzed with FlowJo v10. All steps involving Live Zombie NiR staining, antibody staining, and fixation were performed on ice and in the dark.

Histological analysis

Liver and small intestine tissues were fixed in 4% paraformaldehyde, embedded in paraffin, and sectioned at a thickness of 4 µm. The sections were mounted on slides and stained with Hematoxylin & Eosin (H&E) for morphology analysis or with Sirius Red to assess collagen accumulation. Alternatively, 8-µm thick cryosections of snap-frozen liver samples were mounted on slides and stained with Oil Red O through Washington University Musculoskeletal Research Center. Slides were imaged using Zeiss Axio Scan Z1 (Zeiss) and analyzed using Zen and Fiji-ImageJ software. For the analysis of SHG, deparaffinized liver tissue slides were subjected to 2-photon microscopy. Two-photon microscopy images were collected using a customized dual-laser system (InSight® & Mai Tai®, Spectra-Physics) on a Leica SP8 upright microscope equipped with a 25x, 0.95 numerical aperture water-immersion objective. The Mai Tai® laser was tuned to 920 nm, and signal separation was achieved using three long-pass dichroic beam splitters (FF640-FDi01, FF562-FDi03, and FF495-Di03, Semrock) to generate channels at approximately 390–495 nm (SHG), 495–562 nm, and 562–640 nm. Fluorescence emission was directed to external hybrid photodetectors (Leica). Analysis of the second harmonic signal was carried out using Imaris software in combination with Fiji-ImageJ software.

RNA sequencing

Total RNA integrity was determined using an Agilent Bioanalyzer or 4200 Tapestation. Library preparation was performed with 500 ng to 1 µg of total RNA. Ribosomal RNA was removed using an RNase H method with RiboErase kits (Kapa Biosystems). mRNA was then fragmented in reverse transcriptase buffer by heating to 94°C for 8 minutes. The fragmented mRNA was reverse transcribed to yield cDNA using SuperScript III RT enzyme (Life Technologies) and random hexamers. A second-strand reaction was performed to yield double-stranded cDNA. cDNA was blunt-ended, had an A base added to the 3' ends, and Illumina sequencing adapters were ligated to the ends. Ligated fragments were then amplified for 12–15 cycles using primers incorporating unique dual index tags. Fragments were sequenced on an Illumina NovaSeq X Plus using paired-end reads extending 150 bases. Base calling and demultiplexing were performed with Illumina's bcl2fastq software with a maximum of one mismatch in the indexing read. RNA-seq reads were then aligned to the Ensembl release 101 primary assembly with STAR version 2.7.9a1. Gene counts were derived from the number of uniquely aligned unambiguous reads by Subread:featureCounts (version 2.0.32). Sequencing performance was assessed for the total number of aligned reads, the total number of uniquely aligned reads, and features detected. The ribosomal fraction, known junction saturation, and read distribution over known gene models were quantified with RSeQC version 4.04, and RNA-seq was conducted by the Genome Technology Access Center. All gene counts were then imported into the R/Bioconductor package EdgeR5, and TMM normalization size factors were calculated to adjust for differences in library size across samples. For downstream analysis, the TPM files were directly loaded into R for further analysis. Gene names were standardized, and duplicate entries were removed to retain unique gene features. Genes with zero TPM values across all samples were excluded. To reduce the impact of extreme values and facilitate comparisons, the TPM data were log2-transformed. Batch effects between experimental groups were

corrected using the ComBat function from the sva R package. Normalized data were visualized with boxplots to confirm consistent sample distributions and ensure comparability across datasets. Filtered TPM matrices were then subjected to downstream analyses. DEGs were identified by comparing experimental groups. Log_2FC and p-values were calculated for each gene using unpaired two-sample t-tests and Wilcoxon rank-sum tests. Genes were ranked by log_2FC and p-values, and the Benjamini–Hochberg procedure was used to control the false discovery rate (FDR). Genes with an adjusted *P*-value < .05 and an absolute log_2FC above a set threshold were considered significantly differentially expressed. Preranked GSEA was performed using the GSEA_4.0.3 with MSigDB collections. Significant enrichment was determined by normalized enrichment scores (NES) and adjusted FDR q-values <0.05. GSVA scores were calculated across samples and standardized (z-score normalization) using custom gene sets, including those associated with LXR signaling, which were defined using the GSEABase package. PCA, heatmaps, enrichment plots, and volcano plots, were generated using R (ggplot2, gplots, dplyr, EnhancedVolcano).

16S rRNA sequencing

DNA was extracted using the ZymoBIOMICS®-96 MagBead DNA Kit (Zymo Research). Library preparation for targeted 16S sequencing was performed using the Quick-16S™ Plus NGS Library Prep Kit with V3–V4 primers. Amplification was performed using a qPCR–based method to minimize chimera formation. PCR products were quantified, pooled at equal molarity, and purified. Final libraries were quantified and sequenced on an Illumina® NextSeq 2000™ (600-cycle P1 kit). Positive controls (ZymoBIOMICS® Microbial Community Standard or DNA Standard) and negative controls (blank extraction and library controls) were included to monitor contamination. Sequencing reads were processed with the DADA2 pipeline for denoising and chimera removal. Taxonomy was assigned using UCLUST from QIIME v.1.9.1 with the Zymo Research 16S reference database. Diversity and composition analyses were conducted using QIIME v.1.9.1, and Linear Discriminant Analysis Effect Size (LEfSe) was used to identify taxa with significant group differences. Sequencing and analysis were performed by Zymo Research.

Lipid and metabolite extraction and LC-MS/MS Analysis

Portal venous serum from female mice was collected. Sample preparation and LC-MS/MS for lipid and metabolite profiling were subsequently performed by the Mass Spectrometry Technology Access Center at the McDonnell Genome Institute. Samples were subjected to biphasic extraction using methyl tert-butyl ether. Samples were vortexed 3 times and incubated at –80°C for 1 hour. After phase separation, the upper phase containing lipids was collected and dried using a SpeedVac without applying heat. The lower phase containing polar metabolites was centrifuged, and the resulting supernatant was similarly dried. Dried lipid extracts were reconstituted in 19 µL of methanol:acetonitrile:water (2:1:1, v/v/v), and dried metabolite extracts were reconstituted in 19 µL of 50% methanol. Reconstituted samples were analyzed by LC-MS/MS using a Vanquish Horizon UHPLC system (Thermo Fisher Scientific) equipped with C8 and C18 columns, and coupled to an Orbitrap Tribrid ID-X mass spectrometer (Thermo Fisher Scientific) operating in both positive and negative ion modes with the AcquireX DeepScan workflow. In total, 2,293 features were annotated as metabolites and lipid species. Quantification was performed based on MS1 peak areas (AUC). Imputed data were used for downstream analyses, and statistical analysis and plot generation were performed using R. For PCA, the imputed data were processed by log_{10} transformation, interquartile range filtering at 40%, sum normalization, and autoscaling. Metabolites with an FDR < 0.2 and an FC > 1.5 in SBR-WUSTL0717 compared to SBR were log_{10}-transformed and used for heatmap generation. For generating pie charts and for analyses of individual significant metabolites, we followed the workflow typically used for volcano plots: data were processed by log_2 transformation and sum normalization without autoscaling, and significant metabolites were defined as those with an FDR < 0.1 and an FC > 1.5, according to the standard MetaboAnalyst (https://metaboanalyst.ca) workflow. Finally, the mean log_{10} value of each group was used to analyze the Pearson correlation with liver collagen accumulation.

References

1. Färnegårdh M, Bonn T, Sun S, et al. The three-dimensional structure of the liver X receptor beta reveals a flexible ligand-binding pocket that can accommodate fundamentally different ligands. J Biol Chem 2003;278:38821-8.
2. Fradera X, Vu D, Nimz O, et al. X-ray structures of the LXRalpha LBD in its homodimeric form and implications for heterodimer signaling. J Mol Biol 2010;399:120-32.
3. Meza JC. Steepest descent. WIREs Computational Statistics 2010;2:719-722.
4. Nazareth JL. Conjugate gradient method. WIREs Computational Statistics 2009;1:348-353.
5. Pettersen EF, Goddard TD, Huang CC, et al. UCSF Chimera--a visualization system for exploratory research and analysis. J Comput Chem 2004;25:1605-12.
6. Mukherjee P, Chattopadhyay A, Grijalva V, et al. Oxidized phospholipids cause changes in jejunum mucus that induce dysbiosis and systemic inflammation. J Lipid Res 2022;63:100153.

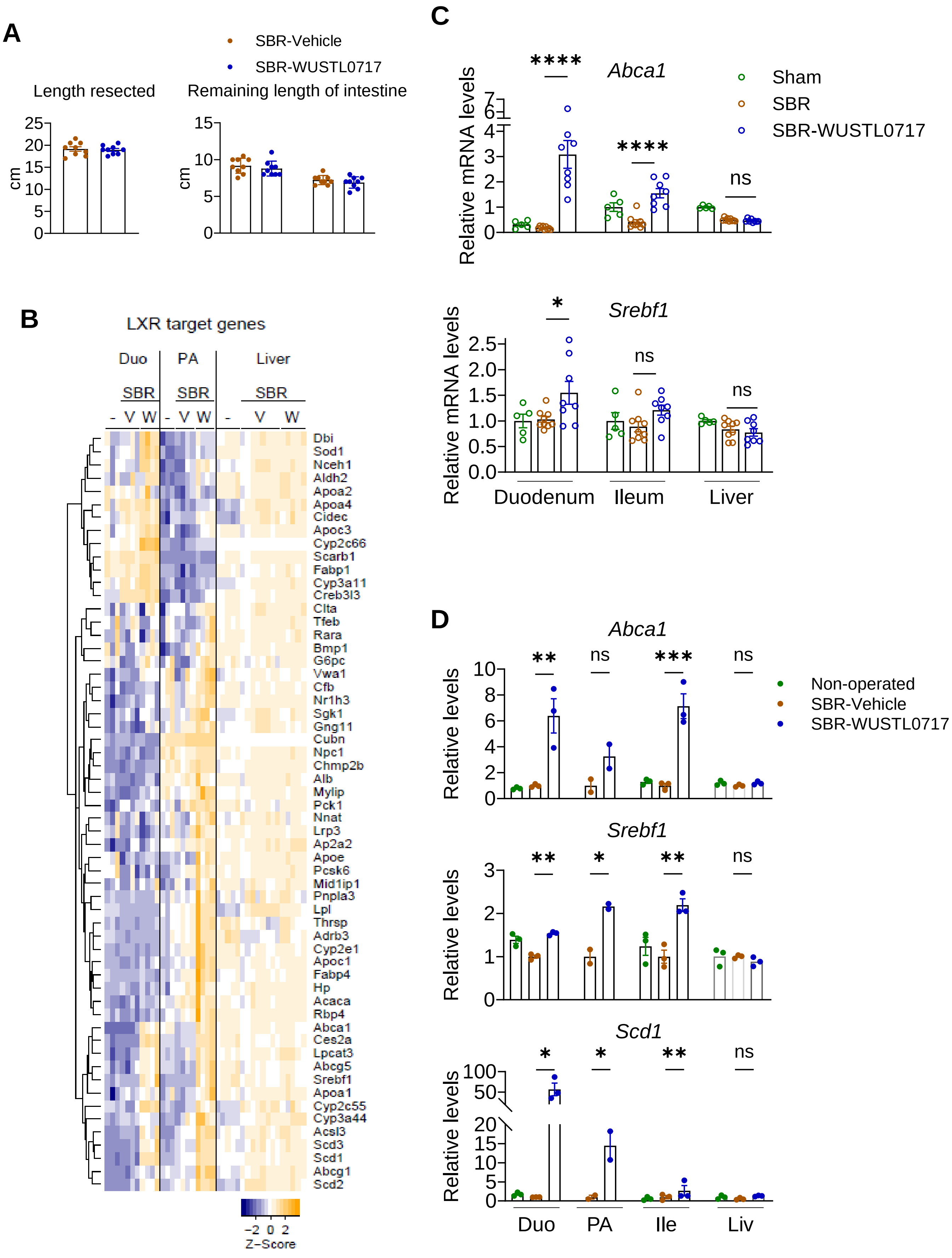

Supplementary Figure 1

Supplementary Figure 1. Intestine-restricted LXR agonist activity following treatment with WUSTL0717.

(A–C) WT male (filled circles) or female (open circles) mice underwent sham or SBR surgery. Starting 3 weeks post-surgery, the mice were treated daily with vehicle or WUSTL0717 (30 mg/kg, p.o.) for 7 weeks (n = 5–9/group).

(A) Length of the small intestine resected during SBR and remaining length at 10 weeks post-SBR.

(B) Heatmap showing LXR target gene expression in the duodenum (Duo), post-anastomosis ileum (PA), and liver (Liv), based on RNA-seq. V, vehicle; W, WUSTL0717.

(C) Transcript levels of LXR target genes in the duodenum, post-anastomosis ileum, or liver, measured by qRT-PCR.

(D) WT male mice (n = 2–3/group) underwent sham or SBR surgery and were treated with vehicle or WUSTL0717 starting 5 days post-surgery for 10 days before sacrifice.

Statistical analysis was performed using one-way ANOVA with Dunnett's (C) or Tukey's HSD (D) test for multiple comparisons. Data are presented as mean ± SEM; *$P < .05$, **$P < .01$, ***$P < .001$, ****$P < .0001$; ns, not significant. Mean ± SEM and p-value thresholds are applied consistently across figures unless otherwise noted.

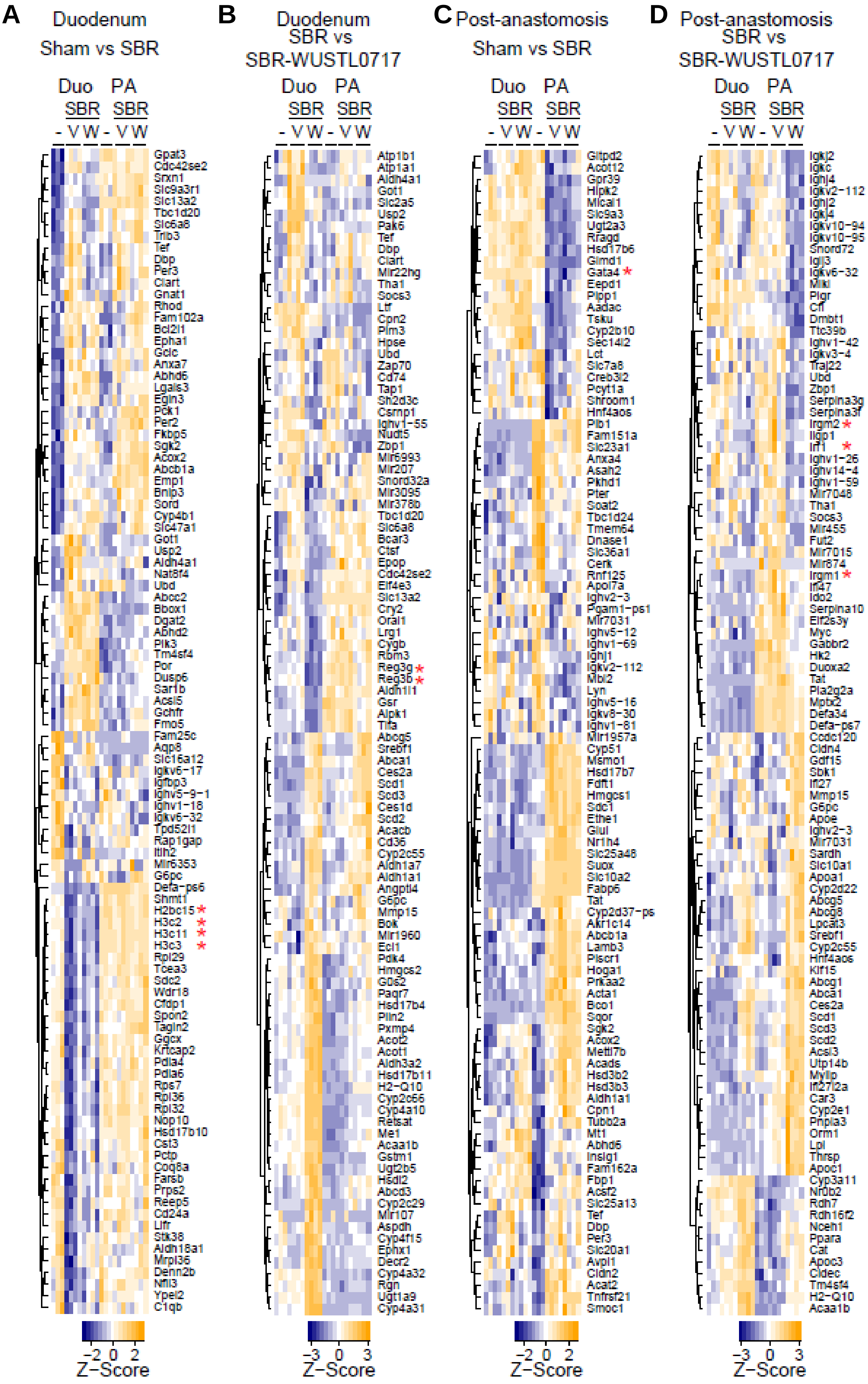

Supplementary Figure 2

Supplementary Figure 2. Intestinal adaptation following SBR and WUSTL0717 treatment.

(A–D) WT male mice underwent sham or SBR surgery. 3 weeks later, mice were treated daily with vehicle or WUSTL0717 (30 mg/kg, p.o.) for 7 weeks before euthanasia. Heatmaps show the top 50 DEGs ($p < 0.05$, FC ≥ 2) identified by RNA-seq in the duodenum (A, B) and post-anastomosis ileum (C, D). Comparisons are sham vs. SBR (A, C) and vehicle- (V) vs. WUSTL0717-treated (W) mice following SBR (B, D) (n = 3–4/group).

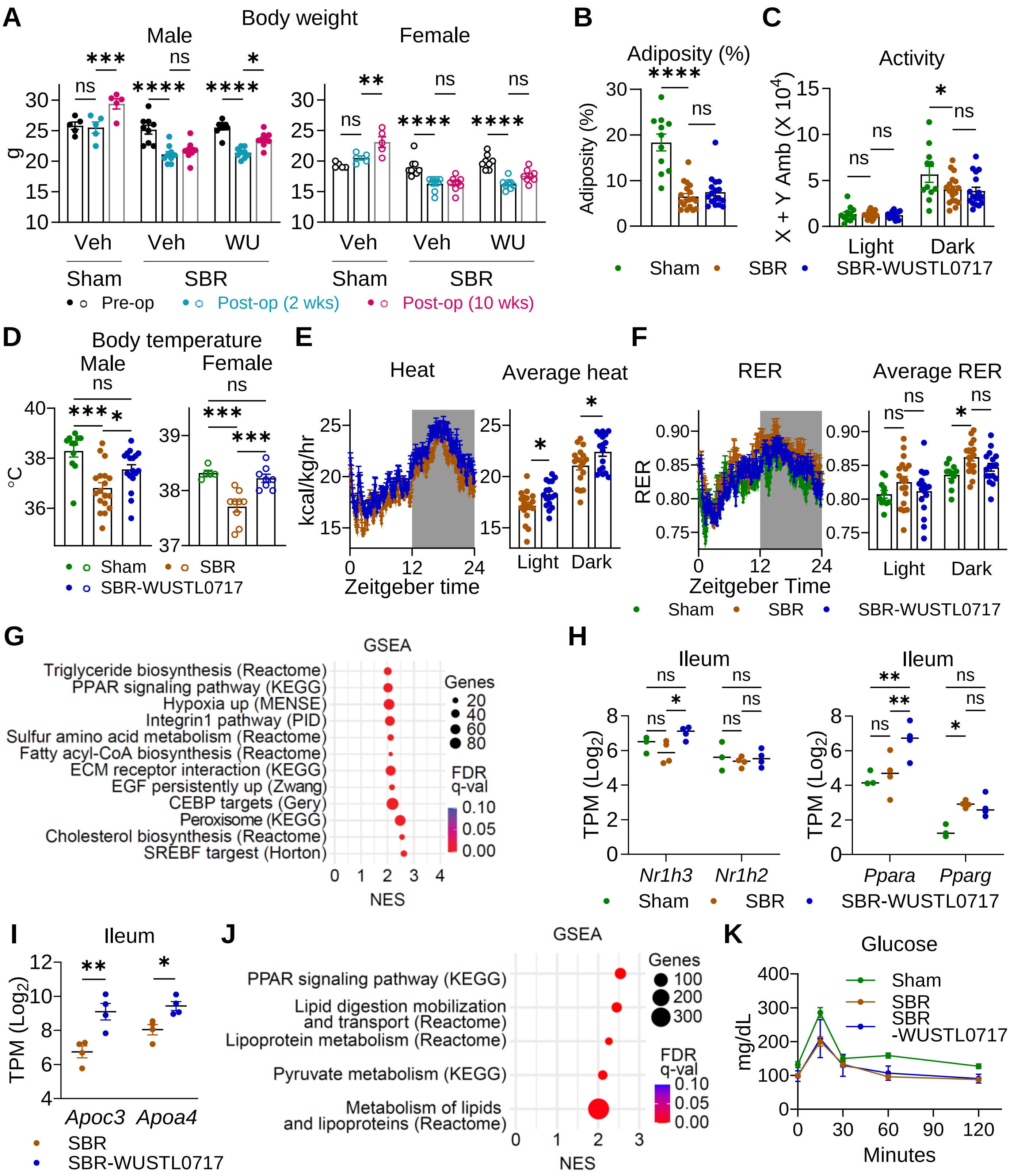

Supplementary Figure 3

Supplementary Figure 3. Metabolic phenotype of WUSTL0717 treatment in WT mice following SBR.

WT male (filled circles) or female (open circles) mice underwent sham or SBR surgery. Beginning 3 weeks post-surgery, mice received vehicle or WUSTL0717 (30 mg/kg, p.o.) daily for up to 7 weeks before euthanasia, unless otherwise specified (I). For the glucose tolerance test (I), treatment continued for 10 weeks, and measurements were performed at 13 weeks post-surgery.

(A) Body weight of mice measured at the indicated weeks (n = 5–9/group). Veh, vehicle; WU, WUSTL0717.

(B–F) At 8–9 weeks post-operation, mice treated with vehicle or WUSTL0717 for 5–6 weeks were subjected to adiposity measurement (B) and core body temperature assessment (D). Mice were individually housed in metabolic cages for a 24-hour evaluation of locomotor activity (C), heat generation (E) and RER (F). The time traces in panels E and F represent group mean values over time, while the adjacent bar plots show the corresponding light- and dark-phase averages for each individual mouse, with each dot representing one mouse. Data from male mice were combined from two independent experiments (male: n = 11–18 /group; female: n = 5–9/group).

(G, J) GSEA of RNA-seq data from post-anastomosis ileum, with enrichment plots illustrating normalized enrichment scores (NES) and associated gene signatures from GSEA of RNA-seq data. (G) Signatures upregulated in SBR relative to sham; (J) signatures upregulated in WUSTL0717-treated group compared to vehicle following SBR (n = 3–4/group).

(H, I) Transcript levels linked to proximal intestinal identity in the post-anastomosis ileum, analyzed by RNA-seq (n = 3–4/group).

(K) Blood glucose levels were measured in overnight-fasted mice 13 weeks post-surgery at 0, 15, 30, 60, and 120 minutes after a 2 g/kg glucose gavage (n = 8–11/group).

Unpaired Student’s t-test (E, I), one-way ANOVA (B, D), or two-way ANOVA with Tukey’s HSD (A, C, F, H, K) was used for statistical analysis.

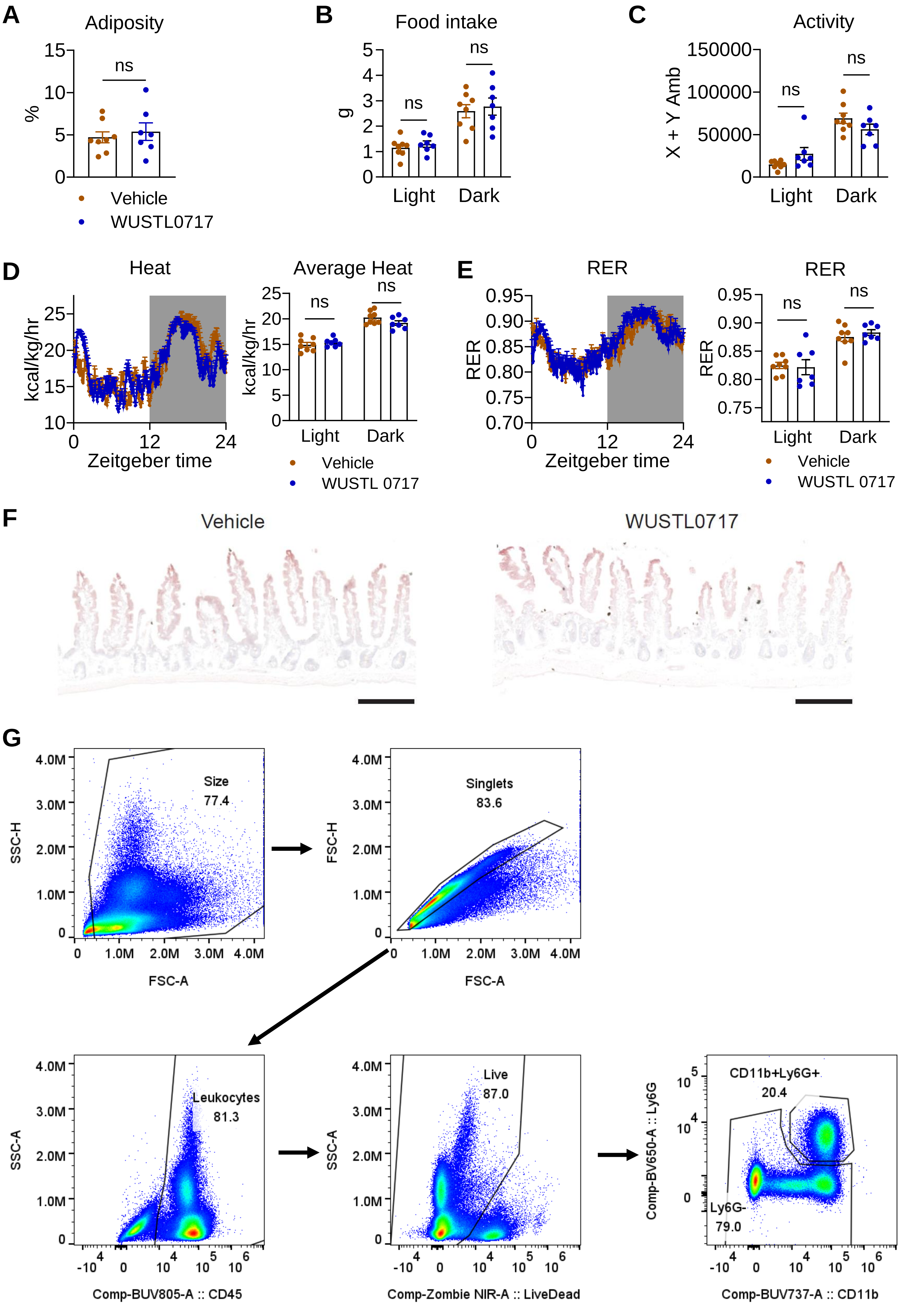

Supplementary Figure 4

Supplementary Figure 4. Metabolic phenotype of WUSTL0717 treatment in WT mice.

(A–G) WT male mice treated daily with either vehicle (n = 8) or WUSTL0717 (30 mg/kg, p.o.) (n = 7) for up to 7 weeks before euthanasia.

(A–E) After 5 weeks of treatment, mice underwent adiposity measurement (A) and were then individually housed in metabolic cages for 24-hour assessment of food intake (B), locomotor activity (C), heat generation (D), and RER (E) under a 12-hour light-dark cycle.

(F) Representative Oil Red O staining of the jejunum from each group after euthanasia (scale bar: 200 μm). Images shown are representative of evaluations from 8 vehicle- and 7 WUSTL0717-treated mice.

(G) Representative flow cytometry plots showing the gating strategy for blood cell suspensions collected after euthanasia, referenced to Figure 4H.

Unpaired Student's t-test was used for statistical evaluation.

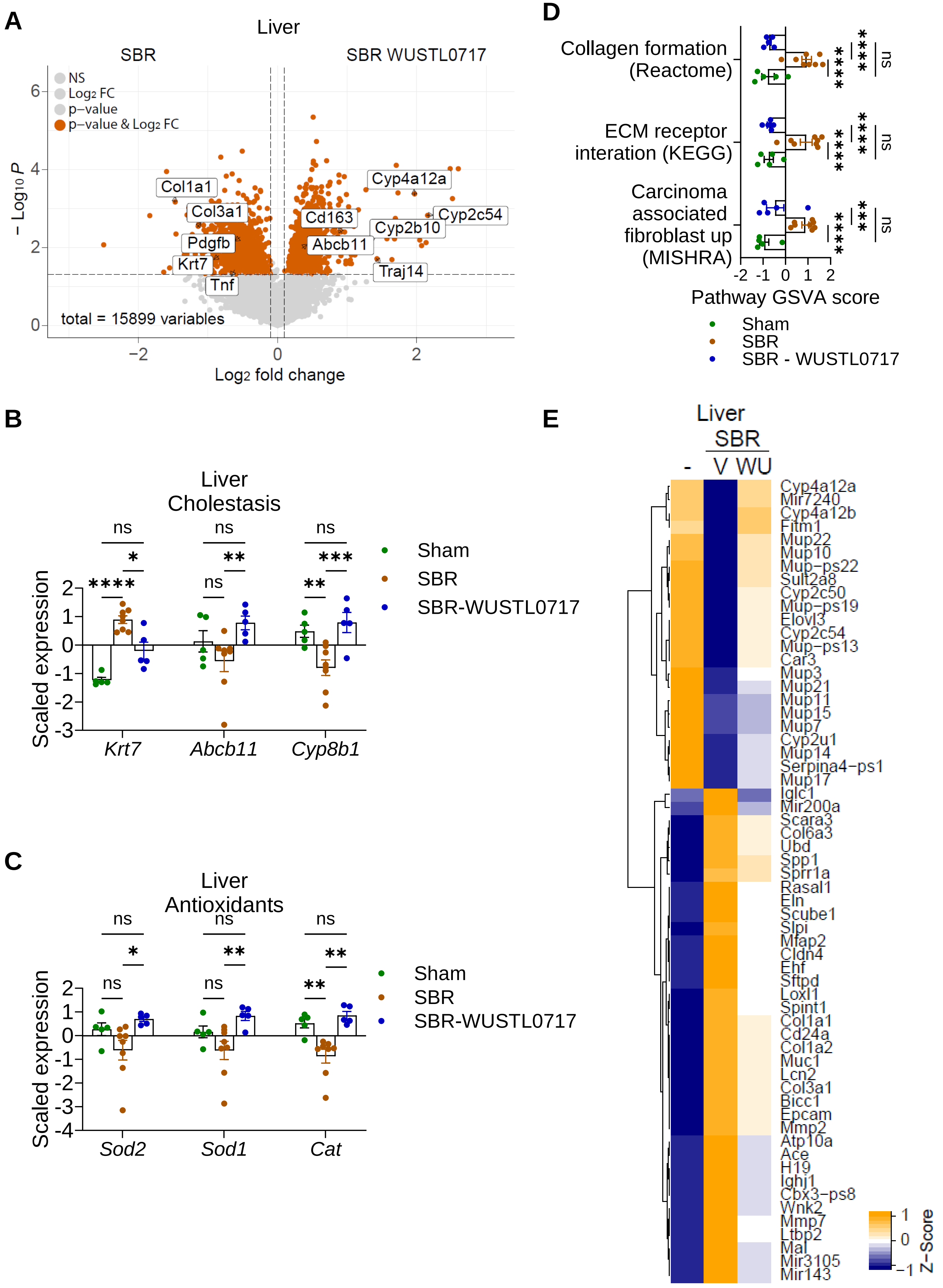

Supplementary Figure 5

Supplementary Figure 5. Analysis of DEGs associated with liver fibrosis in sham or SBR, WUSTL0717-treated groups.

(A, B, D–F) RNA-seq was performed on liver tissue from WT male mice that underwent sham or SBR surgery and were treated daily with vehicle or WUSTL0717 (30 mg/kg, p.o.) for 7 weeks (n = 5–8/group).

(A) Volcano plot showing DEGs in the liver between vehicle- and WUSTL0717-treated groups following SBR. DEGs were identified based on an adjusted p-value < 0.05 and log_2FC thresholds, with significant genes highlighted in orange.

(B) Transcript levels of *Krt7, Abcb11*, and *Cyp8b1* in the liver.

(C) Transcript levels of *Sod2*, *Sod1*, and *Cat* in the liver.

(D) GSVA scores for liver fibrosis-related pathways.

(E) Heatmap of DEGs in the liver, showing the up- or downregulated genes with fold change (*P*-value < .05 and FC ≥ 2) when comparing the vehicle and WUSTL0717-treated groups following SBR. The heatmap also highlights genes with significant differences (*P*-value < .05) between sham and SBR groups. For visualization, a single group mean was normalized to a z-score and shown in the heatmap.

Statistical evaluations used two-way ANOVA (B–D) with Tukey's HSD.

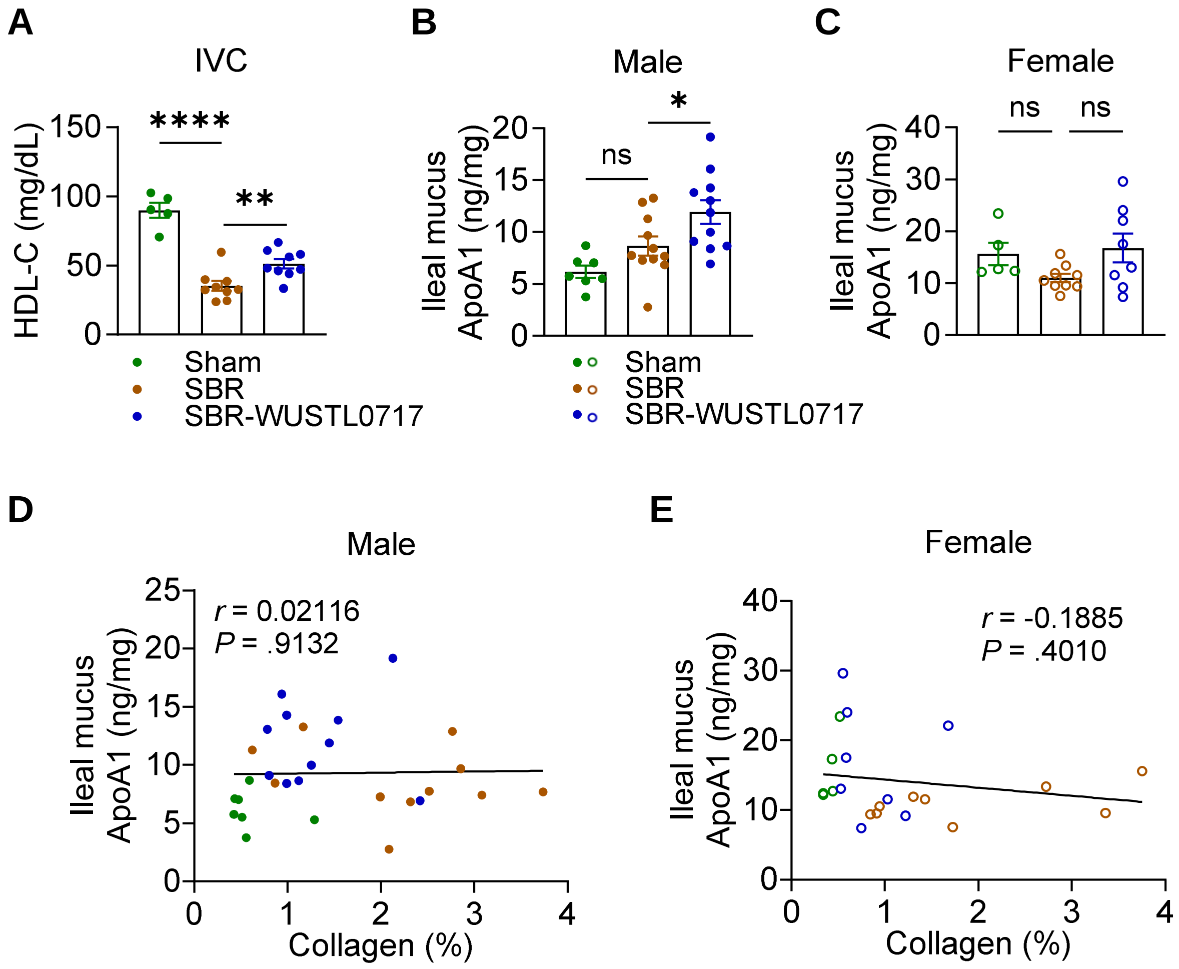

Supplementary Figure 6

Supplementary Figure 6. HDL-C and correlation of ileal mucus ApoA1 with liver fibrosis after WUSTL0717 treatment following SBR.

(A–E) WT male (filled circles) and female (open circles) mice underwent sham or SBR surgery. 3 weeks later, the mice received vehicle or WUSTL0717 (30 mg/kg, p.o.) daily for 7 (A, C, E) or 12 (B, D) weeks, after which they were euthanized for analysis (n = 5–11/group).

(A) Systemic plasma HDL-C levels (IVC) in each group.

(B, C) ApoA1 levels in the ileal mucus layer measured by ELISA and normalized to total protein content.

(D, E) Correlation between ileal mucosal ApoA1 levels (B, C) and liver collagen area (Figure 5C). Each dot represents a matched individual from panels B and C.

Statistical evaluations were performed using unpaired Student's t-test (A), one-way ANOVA with Tukey's HSD (B, C), or Pearson correlation (D-E).

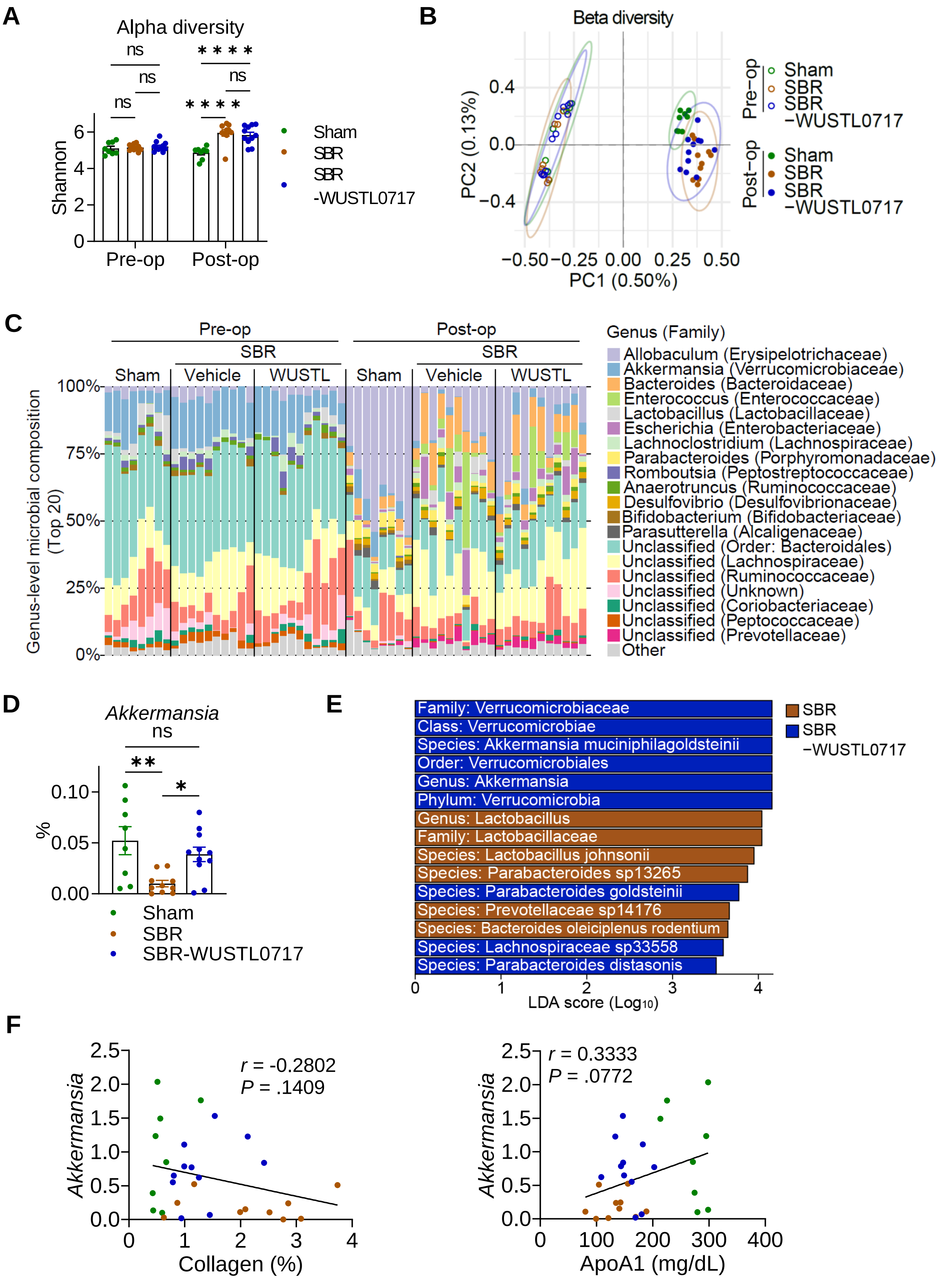

Supplementary Figure 7

Supplementary Figure 7. Fecal microbiome analysis of WUSTL0717-treated mice after SBR.

16S rRNA sequencing was performed on fecal samples from WT male mice collected before surgery and 10 weeks after sham or SBR surgery, following daily treatment with vehicle or WUSTL0717 (30 mg/kg, p.o.) for 7 weeks (n = 8–11/group).

(A) Shannon alpha diversity index.

(B) Bray–Curtis beta diversity index.

(C) Top 20 genera in the microbial composition. Genus and family information are shown in the figure for each group.

(D–F) Analysis of post-operative groups at 10 weeks after surgery.

(D) Percentage of *Akkermansia* in the total microbial composition.

(E) Taxa with LEfSe LDA scores > 3.5 are displayed on a log scale.

(F) Correlation between *Akkermansia* abundance (D) and either liver collagen area or portal venous plasma ApoA1 levels. Each dot represents an individual sample matched to panel D.

One-way ANOVA (D), two-way ANOVA (A) with Tukey's HSD, and Pearson correlation (F) were used for statistical analyses.

Supplementary Table 1. Genotyping primers (mouse)

Primer (mouse)	Forward	Reverse
Apoa1 flox	CGAAGTTATGAATTCTATCTCGCACCT	TGACCAGGATCCATAACTTCGTATAATGT
Apoa1 flox-WT	ACCGTGGATATCTCGCACCTT	TCTGACCAGTACTGGGGGTTA
Villin-Cre	GCCTTCTCCTCTAGGCTCGT	AGGCAAATTTTGGTGTACGG
		TATAGGGCAGAGCTGGAGGA (Internal primer)

Supplementary Table 2. WUSTL0717 instrument settings

LC (Shimadzu UFLC XR) conditions

Compound	WUSTL0717	I.S. (Carbamazepine)
Column	Thermo Betasil C18 5μ, 50x2.1mm	
Mobile phase	A: Water with 0.1% Formic Acid<br>B: Acetonitrile with 0.1% Formic Acid	
Flow rate (mL/minutes)	0.35	
Temperature (°C)	35	
Injection volume(μL)	10	

Gradient elution conditions:

Time (minutes)	Mobile phase A (%)	Mobile phase B (%)
0.2	90	10
0.5	90	10
2.0	5	95
3.0	5	95
4.0	90	10
5.9	90	10

MS (API6500+) conditions

Compound	WUSTL0717	I.S. (Carbamazepine)
MRM(+)	582/181	237.2/194.1
Collision Gas	7	
Curtain GAS	35	
Ion Source Gas1	55	
Ion Source Gas2	50	
Ion Spray Voltage	5500	
Temperature (°C)	550	
Collision Energy	41	26
Declustering Potential	25	136
Entrance Potential	10	
Collision Cell Exit Potential	14	

Supplementary Table 3. *In-Vitro* ADME parameters WUSTL0717.HCL

Compound	Kinetic Solubility (µM)	Mouse PPB (%bound)	HLM $t_{1/2}$ (minutes) / Cl_{int} (µl/minutes/mg)	MLM $t_{1/2}$ (minutes) / Cl_{int} (µl/minutes/mg)
WUSTL0717.HCl	0.29	99.81	12.4 / 112	49 / 18

PPB, plasma protein binding; HLM, human liver microsomes; MLM, mouse liver microsomes; $t_{1/2}$, half-life; Clint, intrinsic clearance.

Supplementary Table 4. qRT-PCR primers (mouse)

**Primer (mouse)**	**Forward**	**Reverse**
Col1a1	GACATCCCTGAAGTCAGCTGC	TCCCTTGGGTCCCTCGAC
bactin	GATCATTGCCTCCTGAGC	GTCATAGTCCGCCTAGAAGCAT
18S	GTAACCCGTTGAACCCCATT	CCATCCAATCGGTAGTAGCG
