# Supplementary material for: A Gut-Restricted Liver X Receptor Agonist Ameliorates Liver Injury in Experimental Short Bowel Syndrome
Source: Gastroenterology. Author manuscript; Available in PMC 2026 May 11. (PMC13160593; doi:10.1053/j.gastro.2025.12.015)
Supplement: 2 [file NIHMS2168396-supplement-2.pdf]

Gastroenterology 2026;170:1017–1032

A Gut-Restricted Liver X Receptor Agonist Ameliorates Liver Injury in Experimental Short Bowel Syndrome

Ayoung Kim,[1] Daniel M. Alligood,[2] Lingaiah Maram,[3,4] Hannah M. Phelps,[2] Michael Cameron,[5] Jacob T. DeRousse,[3,4] Jichang Han,[1] Taylor J. Dunning,[1] Rachel L. Mintz,[1,6] Alex Park,[2] Daniel D. Lee,[1] Deanna L. Davis,[1] Christopher G. Huckstep,[1] Rachael L. Field,[1] Lamees Hegazy,[4,7] Bernd H. Zinselmeyer,[1] Jonathan R. Brestoff,[1] Colin A. Martin,[2] Brad W. Warner,[2,†] Bahaa Elgendy,[3,4] and Gwendalyn J. Randolph[1]

[1]Department of Pathology and Immunology, Washington University School of Medicine, St Louis, Missouri; [2]Department of Surgery, Washington University School of Medicine, St Louis, Missouri; [3]Department of Anesthesiology, Washington University School of Medicine in St Louis, St Louis, Missouri; [4]Center for Clinical Pharmacology, Washington University School of Medicine and University of Health Sciences and Pharmacy, St Louis, Missouri; [5]Department of Molecular Medicine, University of Florida, Jupiter, Florida; [6]Department of Biomedical Engineering, Washington University in St Louis, St Louis, Missouri; and [7]Department of Pharmaceutical and Administrative Sciences, University of Health Sciences and Pharmacy, St Louis, Missouri

HEPATOBILIARY

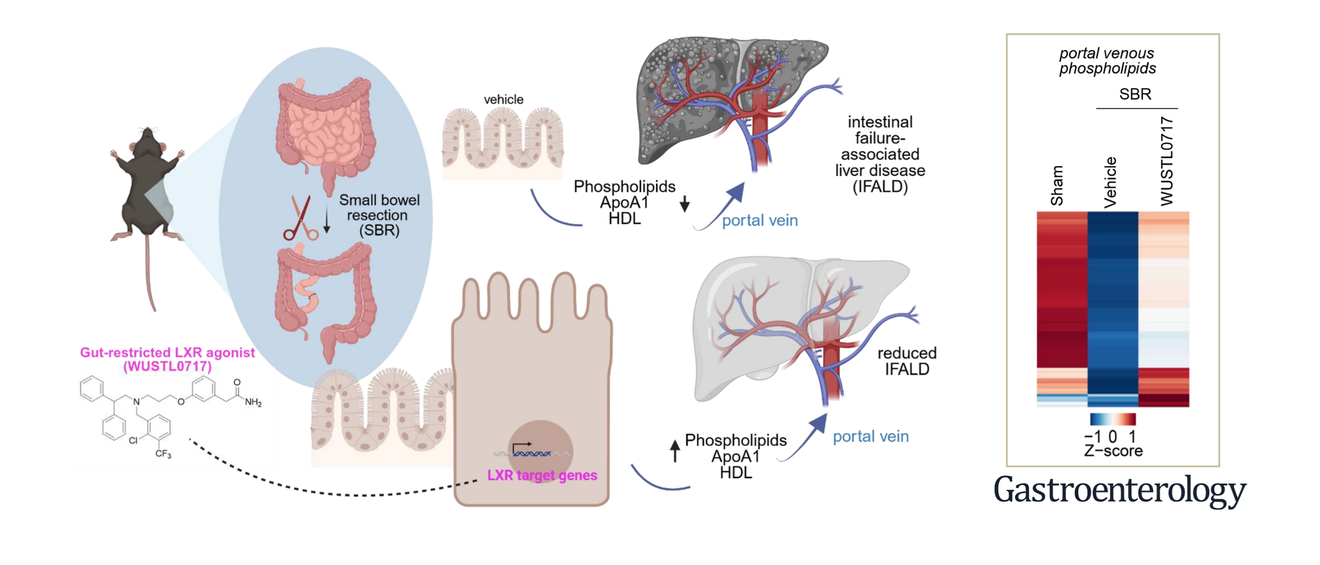

BACKGROUND & AIMS: Short bowel syndrome (SBS) arises from the surgical removal of extensive portions of the small intestine and is associated with high morbidity, including intestinal failure–associated liver disease (IFALD). Earlier studies revealed that orally administered systemic liver X receptor (LXR) agonist suppresses IFALD in mice and implicated intestinally derived high-density lipoprotein (HDL) in liver protection. Here we aimed to move away from the use of systemic LXR agonists because they have failed in clinical trials due to hepatic steatosis and hyperlipidemia, to determine if a gut-restricted LXR agonist could provide hepatoprotection in SBS. **METHODS:** We synthesized and characterized WUSTL0717, an amide analog of GW3965, as a putative gut-restricted LXR agonist, and evaluated its potential to improve the outcomes in a preclinical mouse model of SBS. **RESULTS:** WUSTL0717 exhibited exceptional intestinal retention in pharmacokinetic analyses and activated LXR target genes in the small intestine but not the liver. Whereas small bowel resection lowered many lipid metabolites in portal venous plasma, WUSTL0717 treatment increased portal venous Apolipoprotein A1 (ApoA1), the core protein of HDL, and spared portal venous phospholipids known to be enriched on HDL. Accordingly, intestinal ApoA1 deficiency exacerbated IFALD, and in wild-type mice, portal venous ApoA1 and phospholipids inversely correlated with hepatic collagen accumulation. In addition, WUSTL0717 improved nutrient absorption and promoted body weight restoration in SBS. **CONCLUSIONS:** These data underscore the potential of gut-restricted LXR agonists to preserve metabolic health in the context of SBS. By acting locally in the intestine, WUSTL0717 positively mitigates profibrotic liver injury while avoiding systemic availability.

Keywords: Liver X Receptors; Enterohepatic Circulation; Lipid Transport; Fibrosis; Inflammation.

WHAT YOU NEED TO KNOW

BACKGROUND AND CONTEXT

Liver X receptor activation in the small intestine protects against liver failure after extensive small bowel resection, but only gut-restricted liver X receptor agonists would be translationally feasible. Yet, a gut-restricted liver X receptor agonist has never been characterized.

NEW FINDINGS

Here, we synthesize and study the pharmacokinetics of a gut-restricted liver X receptor WUSTL0717, showing its strong liver X receptor activation restricted to the intestine. WUSTL0717 augments metabolic recovery after bowel resection in mice, protecting the liver against fibrosis in a manner that relies on enterocyte ApoA1, the core protein of high-density lipoprotein, and raises portal venous phospholipids.

LIMITATIONS

Future studies are needed to evaluate the potential of a gut-restricted liver X receptor agonist to protect the liver after gut resection without adverse effects in humans.

CLINICAL RESEARCH RELEVANCE

Therapies for intestinal failure–associated liver disease are needed. There has long been interest in action of liver X receptor limited to the intestine. This is the first full demonstration of a gut-restricted liver X receptor agonist.

BASIC RESEARCH RELEVANCE

We show that WUSTL0717 preserves some phospholipid transport from the intestine through the portal vein that is diminished after small bowel resection. This is likely due to phospholipid transport by high-density lipoprotein and, accordingly, we show that enterocyte-derived ApoA1 protects the liver against inflammatory injury, underscoring major physiological roles of high-density lipoprotein beyond cardiovascular health. We considered the possibility that high-density lipoprotein in the intestinal mucus controls the microbiome to protect the liver but found that its presence in the portal vein was most critical.

HEPATOBILIARY

The small intestine absorbs nutrients to sustain life. Various conditions, including neonatal necrotizing enterocolitis, may require small bowel resection (SBR) that can lead to malabsorption[1] and partial compensatory post-intestinal adaptations,[2,3] sequelae collectively termed short bowel syndrome (SBS).[1] Prolonged SBS leads to morbidity in the liver, referred to as intestinal failure–associated liver disease (IFALD), which involves hepatic steatosis, cholestasis, and fibrosis.[4] Glucagon-like peptide-2 agonists are used clinically to improve intestinal adaptation (NCT05018286). However, no established therapeutics exist for SBS-associated IFALD once it develops, making prevention the primary management goal. Approaches under investigation for IFALD management include omega-3 enriched parenteral lipids[5] and the medium-chain fatty acid cocktail SEFA-6179 (NCT05919680).[6]

Liver X receptors (LXRs), including LXRα (*NR1H3*) and LXRβ (*NR1H2*), are nuclear receptors that govern genes involved in lipid and sterol metabolism.[7] We previously reported that intestinal ATP-binding cassette transporter A1 (*Abca1*), a key LXR target for high-density lipoprotein (HDL) biogenesis, promoted portal venous HDL that protected the liver from lipopolysaccharide-induced injury.[8] Oral administration of the LXR agonist GW3965[9] appeared to have potential therapeutic value by upregulating *Abca1* and increasing portal HDL.[8] However, systemic LXR agonists cause adverse effects, primarily hepatic lipogenesis, hindering their development as treatments for atherosclerosis and other diseases.[10,11] Hence, we reasoned that a gut-restricted LXR agonist might offer safer and more effective therapy. In our search for gut-restricted LXR agonists to treat SBS, we identified WUSTL0717. Reported by GlaxoSmithKline in 2002, this LXR agonist was not further developed, as the slightly more potent analog GW3965 was favored.[12] WUSTL0717 (previously referred to as GW6340) was later described as an intestinally selective compound, with limited published evidence, and as an ester analog of GW3965.[13] However, the GlaxoSmithKline patent (PCT/US01/27622) refers to GW6340 as an amide analog of GW3965, which we designate as WUSTL0717.

Here, we synthesized and carried out pharmacokinetic studies of WUSTL0717 in mice to evaluate its intestinal retention and its effects on SBS and IFALD. We also examined its impact on metabolites in the portal vein, where we found that it primarily spared a reduction in phospholipids within the portal vein caused by SBR. Phospholipids, particularly phosphatidylcholine (PC) species, are major HDL components along with Apolipoprotein A1 (ApoA1) and cholesteryl esters.[14,15] We thus evaluated the impact of intestinal epithelial cell deletion of *Apoa1* on SBR. Our data further solidify the key role of intestinal HDL in protecting the liver through its passage in the portal vein and suggest therapeutic benefits of WUSTL0717 for managing IFALD and improving intestinal functionality, with action restricted to inducing LXR target genes specifically within the intestine.

Materials and Methods

Mice

C57BL/6 wild-type (WT) (JAX 000664) mice were purchased from The Jackson Laboratory. Mice generated by crossing

† Deceased

Abbreviations used in this paper: **ALT, alanine aminotransferase; ApoA1, Apolipoprotein A1; AST, aspartate aminotransferase; HDL, high-density lipoprotein; HDL-C, high-density lipoprotein cholesterol; IFALD, intestinal failure–associated liver disease; LC-MS/MS, liquid chromatography–tandem mass spectrometry; LXR, liver X receptor; PC, phosphatidylcholine; PC O-, ether-linked PC; PE, phosphatidylethanolamine; PE O-, ether-linked PE; qRT-PCR, quantitative reverse transcription polymerase chain reaction; PO, per os; RNA-seq, RNA sequencing; SBR, small bowel resection; SBS, short bowel syndrome; WT, wild type.**

Most current article

© 2026 The Author(s). Published by Elsevier Inc. on behalf of the AGA Institute. This is an open access article under the CC BY license (http://creativecommons.org/licenses/by/4.0/).
0016-5085
https://doi.org/10.1053/j.gastro.2025.12.015

Villin-Cre (JAX 004586) mice with *Apoa1*$^{f/f}$ mice (*Apoa1*$^{\Delta IEC}$)[16] were on a mixed genetic background, predominantly C57BL/6 with additional contributions from FVB/NJ or DBA/2J. Mice were housed at Washington University in St Louis in a 12-hour light-dark cycle in a specific pathogen-free facility with a controlled temperature (21 ± 1°C) and humidity (50% ± 20%). Food and water were provided ad libitum unless otherwise specified. Genotypes were confirmed by polymerase chain reaction using primer pairs listed in Supplementary Table 1, and the absence of Cre-mediated germline recombination was verified by Transnetyx qPCR. All procedures were conducted during the light phase. Age- and weight-matched mice were randomly divided into experimental subgroups based on the distribution. Experimenters were not blinded to genotypes or treatment due to the study design and group size. The controls in all experiments were sibling littermates of the experimental cohort, and siblings, even when of different genotypes, were co-housed throughout all experiments. The studies were approved by the Washington University Animal Studies Committee (protocols 22-0433, 23-0421, and 22-0286).

Synthesis of WUSTL0717, Molecular Modeling and Screening, and Use In Vivo

A detailed synthesis protocol for WUSTL0717 is described at the accompanying submission in protocols.io ("Synthesis of WUSTL0717" available at: https://www.protocols.io/view/synthesis-of-wustl0717-bp2l6dy2kvqe/v1). Molecular modeling, evaluation, and ligand-binding assays are described in the Supplementary Materials and Methods. For in vivo administration, WUSTL0717.HCl was suspended in 0.5% hydroxypropyl methylcellulose and 4% Tween 80 in 60 mM phosphate buffer (pH 7). WUSTL0717 or vehicle was administered orally (per os [PO]) by gavage once daily at 30 mg/kg body weight, starting either 1 week after arrival at the facility or 3 weeks after SBR or sham surgery to allow recovery. For short-term WUSTL0717 administration, mice were treated with WUSTL0717 starting 5 days post-SBR and continued for 10 days. In nonsurgical studies, WUSTL0717 treatment was initiated in WT mice at 8 weeks of age.

Pharmacokinetics

At the specified time point after administration of a single oral gavage dose of WUSTL0717 at 30 mg/kg, 8-week-old WT male mice were euthanized and dissected tissues or plasma were snap-frozen in liquid nitrogen. Untreated mice served as baseline control. For quantification, 5-μL plasma samples were directly loaded onto a 96-well Millipore Multiscreen Solvinert 0.45-μm low-binding polytetrafluoroethylene hydrophilic filter plate. Tissue samples were homogenized with water (1:3 dilution), and 5 μL of the homogenate was loaded onto the filter plate. All plasma and tissue samples were treated with 75 μL of a 90/10 acetonitrile/water solution containing carbamazepine as the internal standard to extract the analyte and precipitate proteins. The plates were agitated on ice for 10 minutes before centrifugation into a collection plate. Separate standard curves were prepared in blank mouse plasma and tissue homogenate and processed in parallel with the samples. The filtrate was directly analyzed by liquid chromatography–tandem mass spectrometry (LC-MS/MS). The high-performance liquid chromatography and MS/MS parameters are provided in Supplementary Table 2. C_{max} (maximum concentration) and T_{max} (time to maximum concentration) were determined from the observed concentration–time data.

Small Bowel Resection

Unless otherwise specified, 8- to 9-week-old male or female mice were subjected to SBR. Mice underwent 75% proximal bowel resection or a sham control operation, as previously described.[8] Briefly, a midline laparotomy was performed to exteriorize the small bowel. In SBR, the small bowel was cut 1 to 2 cm distal to the ligament of Treitz and 6 cm proximal to the ileocecal junction, then reconnected by an anastomosis. In sham operations, the small bowel was transected 6 cm proximal to the ileocecal junction and immediately re-anastomosed. All anastomoses were hand-sewn end-to-end with interrupted 9–0 nylon sutures. After surgery, the mice received a 1-mL bolus of normal saline and were placed in a warm cage inside an incubator. During the night following surgery, they were restricted to water only. On postoperative day 1, a second 1-mL bolus of saline was administered, and a liquid diet (PMI Micro-Stabilized Rodent Liquid Diet LD 101; TestDiet) was introduced. Mice were without food for less than 24 hours after surgery. They remained in the incubator until postoperative day 7, then were moved to standard cages with continued ad libitum access to the liquid diet until euthanasia.

Blood Chemistry and Tissue Lipid Measurements

Portal venous blood (60 μL) was collected with needle bevel facing the intestine, and systemic blood was collected from the inferior vena cava or via cheek bleed. For plasma isolation, blood was collected in EDTA tubes and centrifuged at 1000*g* for 10 minutes at 4°C. For serum isolation, coagulated blood was centrifuged at 2000*g* for 15 minutes at room temperature. Plasma or serum cholesterol, triglycerides, alanine aminotransferase (ALT), and aspartate aminotransferase (AST) were assessed by the DCM Research Animal Diagnostic Laboratory at Washington University. HDL-cholesterol (HDL-C) levels were measured using the HDL-C assay kit (STA-394; Cell Biolabs), and ApoA1 levels by enzyme-linked immunosorbent assay (3750–1HP; Mabtech). Liver cholesterol and triglyceride levels were quantified by the Diabetes Models Phenotyping Core Services at Washington University.

RNA Sequencing

Liver (median right lobe), duodenum, or post-anastomotic ileum from sham- or SBR-operated mice, with or without WUSTL0717 treatment, were subjected to RNA sequencing (RNA-seq) by the Genome Technology Access Center. Data were analyzed as detailed in the Supplementary Materials and Methods and deposited in the Gene Expression Omnibus (accession GSE287046).

Lipid and Metabolomics LC-MS/MS Analysis

Mouse portal venous serum samples were extracted and analyzed by LC-MS/MS on a Vanquish Horizon UHPLC system coupled to an Orbitrap Tribrid ID-X mass spectrometer (Thermo Fisher Scientific) by the Mass Spectrometry Technology Access Center, as detailed in the Supplementary Materials and Methods.

Statistics

Data are presented as mean ± standard error of the mean (SEM). Statistical significance was assessed using unpaired Student *t* test or 1-way/2-way analysis of variance followed by Tukey's honestly significant difference for multiple group comparisons, unless otherwise specified. All statistical analyses were performed using Prism software (GraphPad), except where noted. Statistical approaches for high-dimensional datasets, including RNA-seq, 16S ribosomal RNA sequencing, and lipidomics, are described in their respective subsections in the Supplementary Materials and Methods. Details on significance levels, sample sizes, and specific statistical tests are provided in the figure legends.

Results

Synthesis and In Vivo Pharmacokinetics of WUSTL0717 Reveal Intestinal Retention

WUSTL0717 was synthesized in a multi-step process, ensuring high yield and purity[17] (Figure 1*A*; details in protocols.io, available at: https://www.protocols.io/view/synthesis-of-wustl0717-bp2l6dy2kvqe/v1). LanthaScreen Time-Resolved Förster Resonance Energy Transfer (TR-FRET) assays revealed that GW3965 exhibited ∼5-fold higher potency compared with WUSTL0717 in binding to LXR*β* (Figure 1*B*). In contrast, WUSTL0717 demonstrated enhanced activity in a luciferase assay, showing ∼2-fold greater potency than GW3965 (Figure 1*C*). Molecular modeling of WUSTL0717 in the ligand-binding pocket of LXR*α* and LXR*β* revealed that, similar to GW3965, its chlorotrifluoromethyl benzyl groups primarily formed hydrophobic interactions. However, the amide group of WUSTL0717 exhibited distinct interactions, engaging Arg232 in LXR*α* and with Leu330 and Glu281 in LXR*β*. In contrast, the carboxyl group of GW3965 interacted with Arg305 and Arg232 in LXR*α*, and with Leu330 and R319 in LXR*β* (Figure 1*D*). In vitro absorption, distribution, metabolism, and excretion assessments revealed that WUSTL0717 has very low kinetic solubility (0.29 *μ*M) and high mouse plasma protein binding (99.81%). It showed metabolic stability in mouse liver microsomes ($t_{1/2}$ = 49 minutes), but less stability in human microsomes ($t_{1/2}$ = 12.4 minutes), indicating species-dependent metabolism (Supplementary Table 3). In vivo pharmacokinetic studies, in which a single dose of WUSTL0717 (30 mg/kg) was administered to individual mice and the distribution of the drug followed over a 24-h period, revealed a strong but transient drug signal in the duodenum, followed by the jejunum and ileum. Drug distribution to the liver was detected at levels substantially lower than those in the intestine (Figure 1*E* and *F*). Complete clearance of the drug from all sites occurred within 24 hours (Figure 1*E* and *F*). At most sites, peak accumulation was observed at 0.5 to 3 h after dosing, and at all sites, except the feces, waned after 3 hours. Fecal detection of WUSTL0717 peaked at 6 hours and waned thereafter (Figure 1*E* and *F*). We conclude that orally administered WUSTL0717 is a potent LXR agonist with activity primarily confined to the small intestine before undergoing rapid catabolism.

WUSTL0717 Activates Liver X Receptor Target Genes in the Intestine But Not the Liver in a Mouse Model of SBS

To investigate whether WUSTL0717 can activate intestinal LXR target genes in an SBS model, we used a mouse SBR model that induces liver fibrosis,[8,18] wherein 75% of the proximal small intestine is resected (Figure 2*A* and Supplementary Figure 1*A*), while preserving the duodenum and the terminal ileum. WUSTL0717 (30 mg/kg, PO) or vehicle was administered daily to mice for 7 weeks, starting 3 weeks after SBR. This design allowed the mice to recover from the surgery before drug exposure (Figure 2*B*). To investigate WUSTL0717 efficacy, bulk RNA-seq was performed on the duodenum and the post-anastomotic ileum of the intestine as well as the liver following SBR. Gene set variation analysis of LXR target pathways identified from the Molecular Signatures Database (MSigDB) revealed increased LXR activity in the duodenum and ileum but not in the liver (Figure 2*C* and Supplementary Figure 1*B*). Key LXR target genes, including *Abca1*, *Srebf1*, and *Scd1* were upregulated in the RNA-seq dataset and confirmed by quantitative reverse transcription polymerase chain reaction (qRT-PCR) in both sexes after the 7-week treatment (Figure 2*D* and *E*, and Supplementary Figure 1*C*), with similar results observed after a shorter 10-day treatment (Supplementary Figure 1*D*). A heatmap of the top 50 genes altered in the duodenum after SBR showed marked downregulation of histone-encoding genes, a feature not rescued by WUSTL0717 (Supplementary Figure 2*A*). This may relate to chromosomal remodeling as the intestine adapts to a shortened absorptive surface. In the duodenum comparison of SBR vs SBR-WUSTL0717, messenger RNAs for antimicrobial peptides *Reg3g* and *Reg3b* were reduced (Supplementary Figure 2*B*), consistent with earlier evidence that bile acids and LXRs regulate antimicrobial peptides.[19] Supporting the shift in regional identity, with the post-anastomotic ileum adopting features of the more absorptive duodenum, the transcription factor *Gata4*, a key regulator of jejunal and ileal identity,[20] was downregulated after SBR and not restored by WUSTL0717 (Supplementary Figure 2*C*). In contrast, WUSTL0717 upregulated numerous LXR targets in the post-anastomotic ileum, while reducing expression of *Irf1*, a generator of the immune-modulatory metabolite itaconate in the tricarboxylic acid cycle,[21] together with host defense genes *Irgm1* and *Irgm2* (Supplementary Figure 2*D*). Collectively, these results indicate that WUSTL0717 induces intestinal LXR targets, downregulates select host defense-associated genes, and spares activation of hepatic LXR targets.

WUSTL0717 Favors Nutrient Acquisition and Metabolic Health Following Small Bowel Resection

Clinically, extensive SBR hinders weight gain and typically necessitates intravenous supplementation to maintain nutrition.[22,23] Indeed, mice lost weight in the first 2 weeks after SBR and failed to regain it between 2 and 10 weeks (Figure 3*A* and Supplementary Figure 3*A*). Treatment with

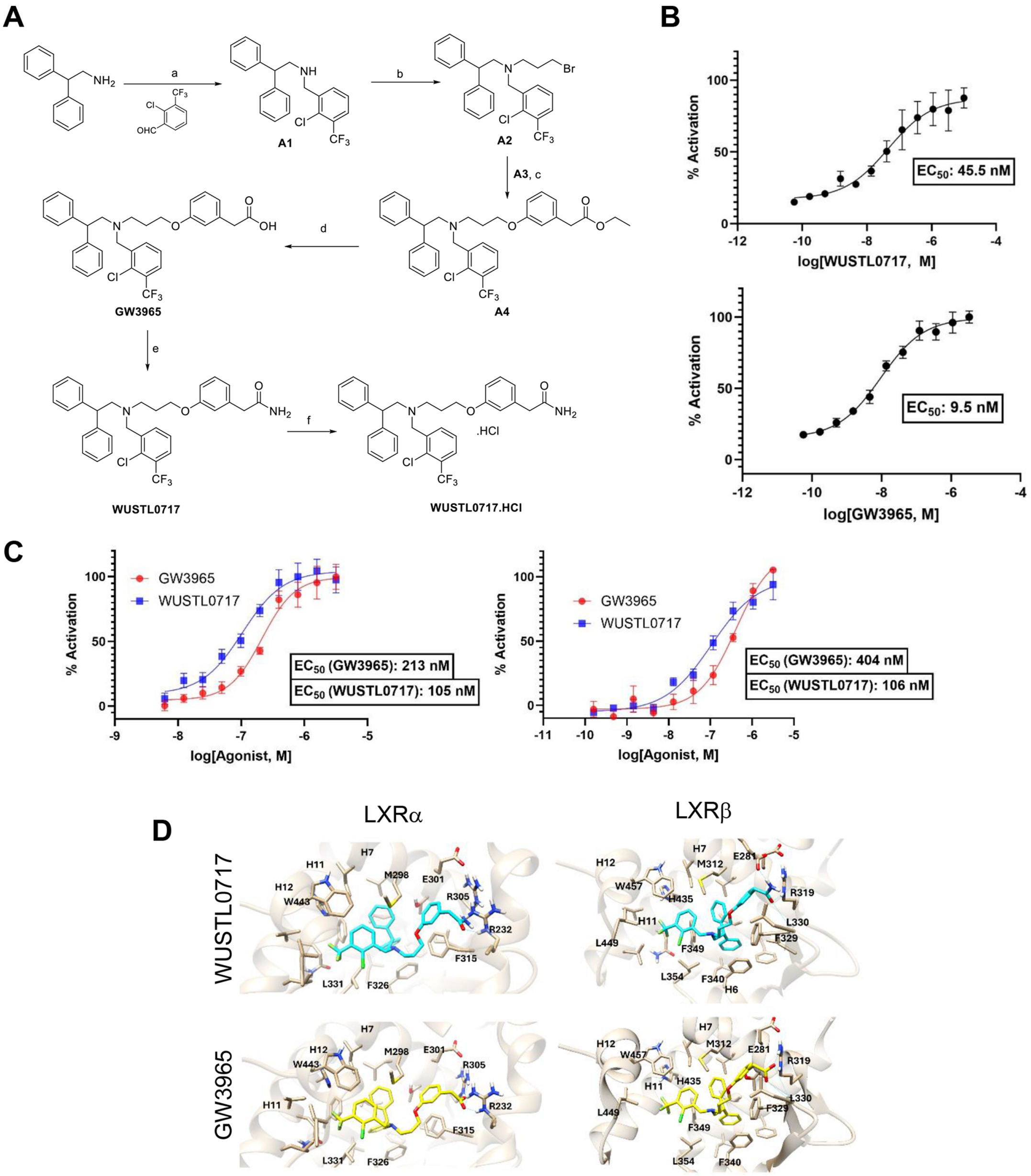

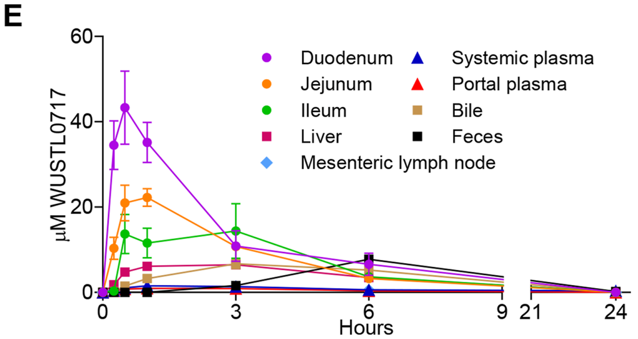

F

Sites	C_{max} (μM)	T_{max} (h)
Duodenum	43.332	0.5
Jejunum	22.268	1
Ileum	14.390	3
Systemic plasma	1.727	3
Portal plasma	0.950	1
Mesenteric Lymph Node	1.323	3
Liver	6.505	3
Bile	6.729	3

Figure 1. Synthesis overview and pharmacokinetic profile of WUSTL0717. (*A*) Chemical reaction steps for WUSTL0717 synthesis. Reaction conditions are described in detail in protocols.io (available at: https://www.protocols.io/view/synthesis-of-wustl0717-bp2l6dy2kvqe/v1). (*B* and *C*) Dose-response curve of WUSTL0717 and GW3965 in LanthaScreen LXR-β coactivator assay (*B*) and cell-based LXRα and LXRβ activation luciferase assay (*C*). Curves were fitted to measurements from 4 wells per concentration, using 4-variable nonlinear regression. (*D*) Binding poses of WUSTL0717 (cyan) in LXRα and LXRβ; and GW3965 (yellow) in LXRα and LXRβ. Hydrogen bonding interactions are illustrated as *blue lines*. (*E* and *F*) Pharmacokinetic analysis of WUSTL0717. (*E*) Tissue distribution of WUSTL0717 after a single 30-mg/kg oral dose in male mice (n = 5 per time point). Fecal samples from each time point were pooled into 1 sample. Graph depicts mean ± standard error of the mean (SEM). (*F*) Table of the mean values of C_{max} and T_{max}.

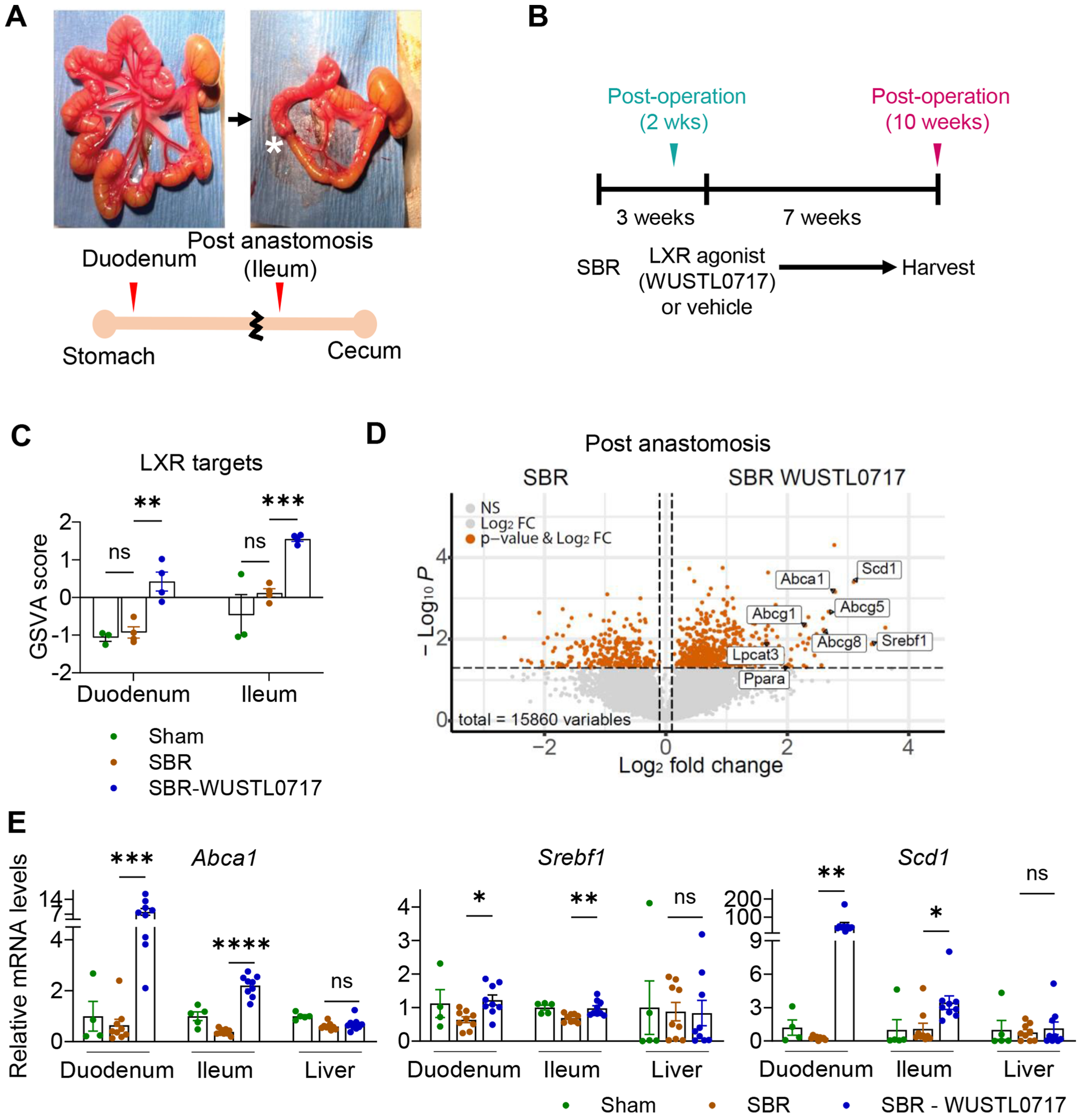

Figure 2. WUSTL0717 exhibits small intestine–restricted LXR agonist activity following SBR. (*A*) Images and schematic representations of the mouse small intestine before and after SBR. *White star*: the anastomosis region. (*B*) Scheme of the SBR procedure and vehicle or WUSTL0717 dosing schedule. WT mice underwent sham or SBR operation. Beginning 3 weeks post-surgery, the mice received either vehicle or WUSTL0717 (30 mg/kg, PO) daily for 7 weeks before euthanasia. (*C*) Gene set variation analysis of LXR target genes from RNA-seq data in male mice subjected to sham (vehicle) or SBR with vehicle or WUSTL0717 treatment (n = 3–4/group). (*D*) Volcano plot of the post-anastomosis ileum from RNA-seq showing differentially expressed genes (DEGs) between vehicle- and WUSTL0717-treated SBR groups. DEGs were defined as adjusted *P* value < .05 and $\log_2$ fold-change thresholds, with significant genes in *orange* (n = 4). (*E*) Transcript levels of LXR target genes in the duodenum, post-anastomosis ileum, and the liver from mice subjected to sham (vehicle) or SBR with vehicle or WUSTL0717 treatment (n = 4–9), analyzed by qRT-PCR. Statistical analysis was performed using 1-way analysis of variance (ANOVA) (*E*) or 2-way ANOVA (*C*) with Tukey's honestly significant difference for multiple comparisons. Mean ± SEM; **P* < .05, ***P* < .01, ****P* < .001, *****P* < .0001; ns, not significant. Mean ± SEM and *P* value thresholds are applied consistently across figures unless otherwise noted.

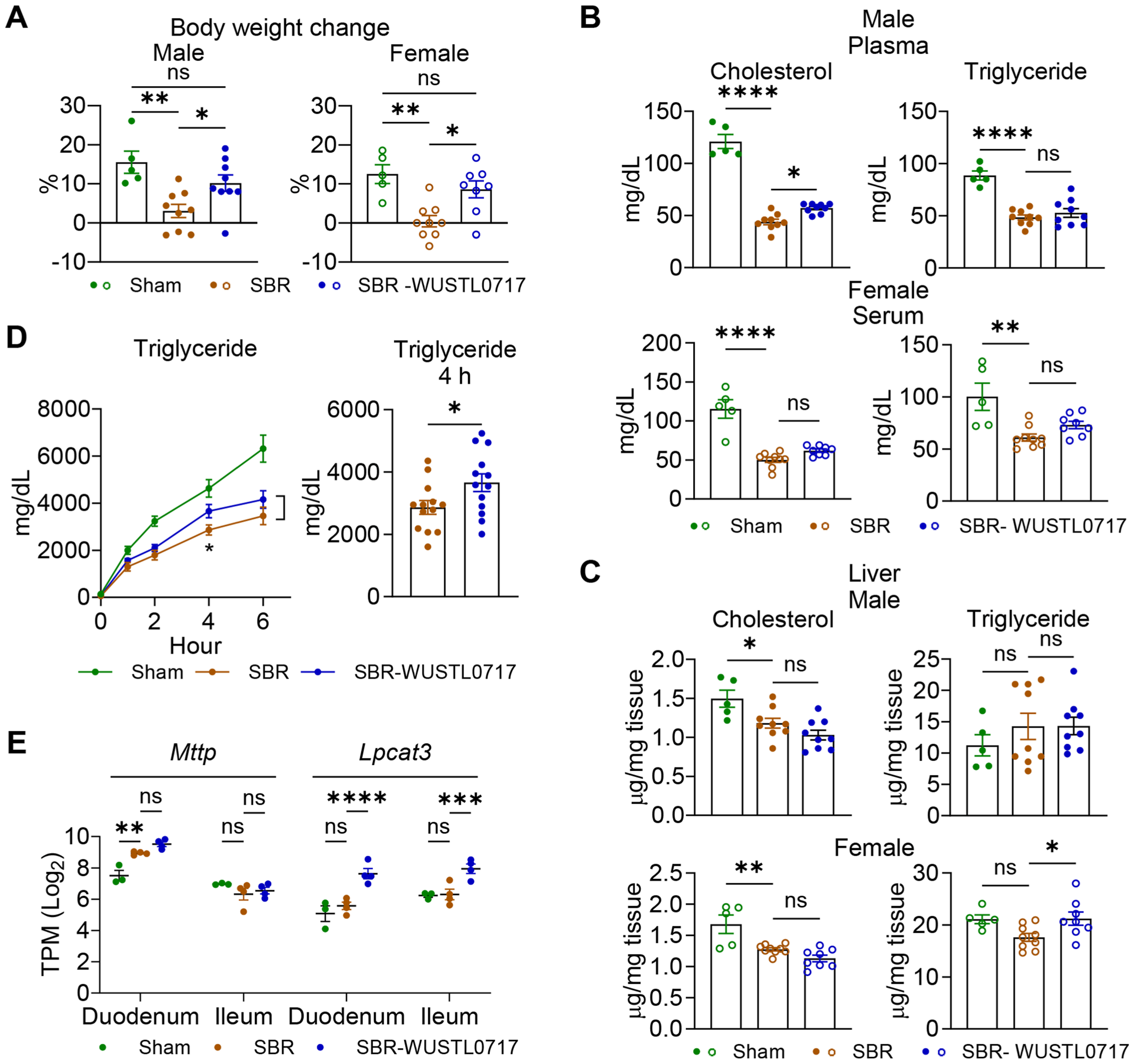

Figure 3. Rescue of body weight loss induced by SBR through intestinal-restricted LXR agonism. (*A–C*, *E*) WT male (*filled circles*) or female (*open circles*) mice underwent sham or SBR operation. Beginning 3 weeks post-surgery, the mice received either vehicle or WUSTL0717 (30 mg/kg, PO) daily for up to 7 weeks (n = 5–9/group). (*A*) Percentage change in body weight, calculated as (week 10 – week 2) post operation divided by the body weight at week 2. (*B*) Cholesterol and triglyceride levels from males (plasma) and females (serum). (*C*) Liver cholesterol and triglyceride levels. (*D*) Mice 10 to 11 weeks post operation, treated with vehicle or WUSTL0717 for 7 to 8 weeks, were used. Data from 2 independent experiments were combined. Plasma triglyceride levels were measured at 0, 1, 2, 4, and 6 hours after a 10 μL/g olive oil gavage, preceded by a 500 mg/kg Tyloxapol intravenous injection (n = 12–14/group). (*E*) *Mttp* and *Lpcat3* transcript levels (TPM, transcripts per million) in the duodenum and the post-anastomosis ileum by RNA-seq (n = 3–4/group). Unpaired Student *t* test (*D*), 1-way analysis of variance (ANOVA) (*A–C*) or 2-way ANOVA (*E*) with Tukey's honestly significant difference statistical test was applied.

WUSTL0717 promoted ~10% gain in body weight between 2 and 10 weeks, similar to the normal growth rate in C57/BL6 mice over 8 weeks (Jackson Laboratory, Body Weight Information for Aged B6 [000664], https://www.jax.org) and similar to sham (Figure 3*A* and Supplementary Figure 3*A*). Both adiposity and activity decreased after SBR and remained unchanged with WUSTL0717 treatment (Supplementary Figure 3*B* and *C*). However, WUSTL0717 reversed the reduction in body temperature (Supplementary Figure 3*D*), consistent with increased metabolic heat production (Supplementary Figure 3*E*). Compared with sham, SBR diminished use of fatty acids as a fuel source, as evidenced by increased respiratory exchange ratio, which was not altered by WUSTL0717 (Supplementary Figure 3*F*). SBR profoundly reduced plasma cholesterol and triglycerides (Figure 3*B*). Blood triglyceride levels, as well as hepatic cholesterol and triglyceride levels, were not increased

by WUSTL0717 in males or females, although in females, liver triglycerides were restored to sham levels by WUSTL0717, indicating that the gut-restricted LXR agonist did not promote hypertriglyceridemia or adverse lipogenesis in the liver (Figure 3*B* and *C*).

The top-ranked pathways upregulated after SBR in the post-anastomosis ileum involved triglyceride, fatty acid, and cholesterol metabolism, along with signaling pathways associated with peroxisome proliferator-activated receptors, CCAAT-enhancer-binding proteins, integrins, and epidermal growth factor, perhaps as compensation for reduced absorptive capacity (Supplementary Figure 3*G*). SBR itself also resulted in elevated *Pparg* (Supplementary Figure 3*H*), whereas genes highly expressed in the proximal region of the small intestine[24] such as *Ppara, Apoc3*, and *Apoa4* were upregulated following WUSTL0717 treatment (Supplementary Figure 3*H* and *I*). *Nr1h3* encoding LXRα was elevated by WUSTL0717, suggesting an autoactivation loop (Supplementary Figure 3*H*). By contrast, *Nr1h2* encoding LXRß was unchanged by WUSTL0717 (Supplementary Figure 3*H*). Overall, Gene Set Enrichment Analysis pathways elevated by WUSTL0717 included peroxisome proliferator-activated receptor signaling, lipid and lipoprotein metabolism, pyruvate metabolism, lipid digestion mobilization, and transport (Supplementary Figure 3*J*). We thus assessed intestinal chylomicron secretion as a measure of intestinal lipid absorption and release into the circulation. SBR significantly reduced this output (Figure 3*D*), as previously published,[25] but WUSTL0717 partially restored it (Figure 3*D*). By comparison, glucose uptake after glucose gavage was unaffected by WUSTL0717, supporting its specificity for affecting lipid and cholesterol metabolism (Supplementary Figure 3*K*). Although chylomicron synthesis genes like *Mttp* were not upregulated by WUSTL0717, WUSTL0717 increased intestinal *Lpcat3* (Figure 3*E*), an enzyme that improves lipid absorption by augmenting membrane permeability.[26] Taking these data together, we conclude that WUSTL0717 positively affects metabolism, favorably remodeling the intestine in a manner that ameliorates metabolic deficits brought on by SBR.

Metabolic Impact of WUSTL0717 Administered to Healthy Wild Type Mice

The increase in chylomicron output by WUSTL0717 after SBR (Figure 3*D*) might relate to correction of SBR-linked impairments in lymphatic transport,[27] unfavorable changes in the villus surface after SBR, or an independent impact on chylomicron synthesis and secretion. To investigate these alternatives and characterize metabolic effects under steady-state conditions, we next administered WUSTL0717 (30 mg/kg, PO) daily for 7 weeks to unoperated C57BL/6 WT mice. Representative LXR target genes were upregulated in the small intestine, but not in the liver (Figure 4*A*). Body weight, adiposity, food intake, activity, heat generation, and respiratory exchange ratio were comparable between vehicle- and WUSTL0717-treated mice (Figure 4*B*; Supplementary Figure 4*A*–*E*). Blood cholesterol and triglyceride levels as well as hepatic cholesterol levels, were unchanged, whereas liver triglyceride levels showed a modest WUSTL0717-induced increase within physiological range (Figure 4*C* and *D*).[28] Oil Red O staining was comparable between groups, indicating minimal lipid accumulation in either the liver or small intestine following WUSTL0717 treatment (Figure 4*E* and Supplementary Figure 4*F*). Intestinal triglyceride absorption, reflected by chylomicron secretion, was indeed elevated by WUSTL0717 (Figure 4*F*). Liver enzymes ALT and AST levels remained normal in plasma, indicating that WUSTL0717 did not promote liver damage (Figure 4*G*). Furthermore, whereas systemic LXR agonists previously caused adverse neutrophil reductions in mice and humans,[29] gut-restricted WUSTL0717 did not alter neutrophil counts or percentages among leukocytes in systemic (inferior vena cava) or portal blood (Figure 4*H* and Supplementary Figure 4*G*). These data indicate that long-term WUSTL0717 treatment in healthy mice minimally affects metabolism, lipid status, or leukocyte homeostasis. It modestly elevates chylomicron secretion, mirroring our observations following SBR, reflecting a direct drug effect on lipid absorption rather than adaptation or lymphatic changes specific to SBS.

WUSTL0717 Protects the Liver From Fibrotic Injury After Small Bowel Resection

To examine the intestine-restricted effects of WUSTL0717 on liver fibrosis, we performed RNA-seq on liver samples from sham- or SBR-operated mice. Principal component analysis revealed distinct clustering of SBR and sham-operated groups. WUSTL0717 diminished this segregation, shifting profiles toward the sham cohort (Figure 5*A*). Blood ALT and AST activities indicated reduced liver damage in the WUSTL0717-treated male mice, with a similar ALT reduction in females (Figure 5*B*). Collagen accumulation in the liver following SBR was reversed by WUSTL0717, as shown by Sirius Red staining and second-harmonic generation imaging (Figure 5*C* and *D*). Elevated *Col1a1* transcripts (Supplementary Materials and Methods, Supplementary Table 4), a key contributor to liver fibrosis, were also reduced (Figure 5*E*). Similar reductions in Sirius Red staining and *Col1a1* expression were observed in females (Figure 5*C* and *E*). Differentially expressed genes revealed that fibrosis-associated genes were significantly downregulated in the liver following WUSTL0717 treatment after SBR, including those for collagen accumulation (*Col1a1, Col3a1*), stellate activation and extracellular matrix remodeling (*Tgfb1, Ltbp1, Pdgfb*), and inflammatory cytokines (*Tnf*) (Figure 5*F* and Supplementary Figure 5*A*). Moreover, thyroid receptor β (*Thrb*), downregulated after SBR, was restored by WUSTL0717 treatment (Figure 5*F*), highlighting its therapeutic potential, especially as a thyroid hormone receptor beta agonist was recently approved by the Food and Drug Administration for metabolic dysfunction–associated steatohepatitis with fibrosis.[30] Genes associated with cholestasis were also affected. SBR increased *Krt7* (Keratin 7)[31] while

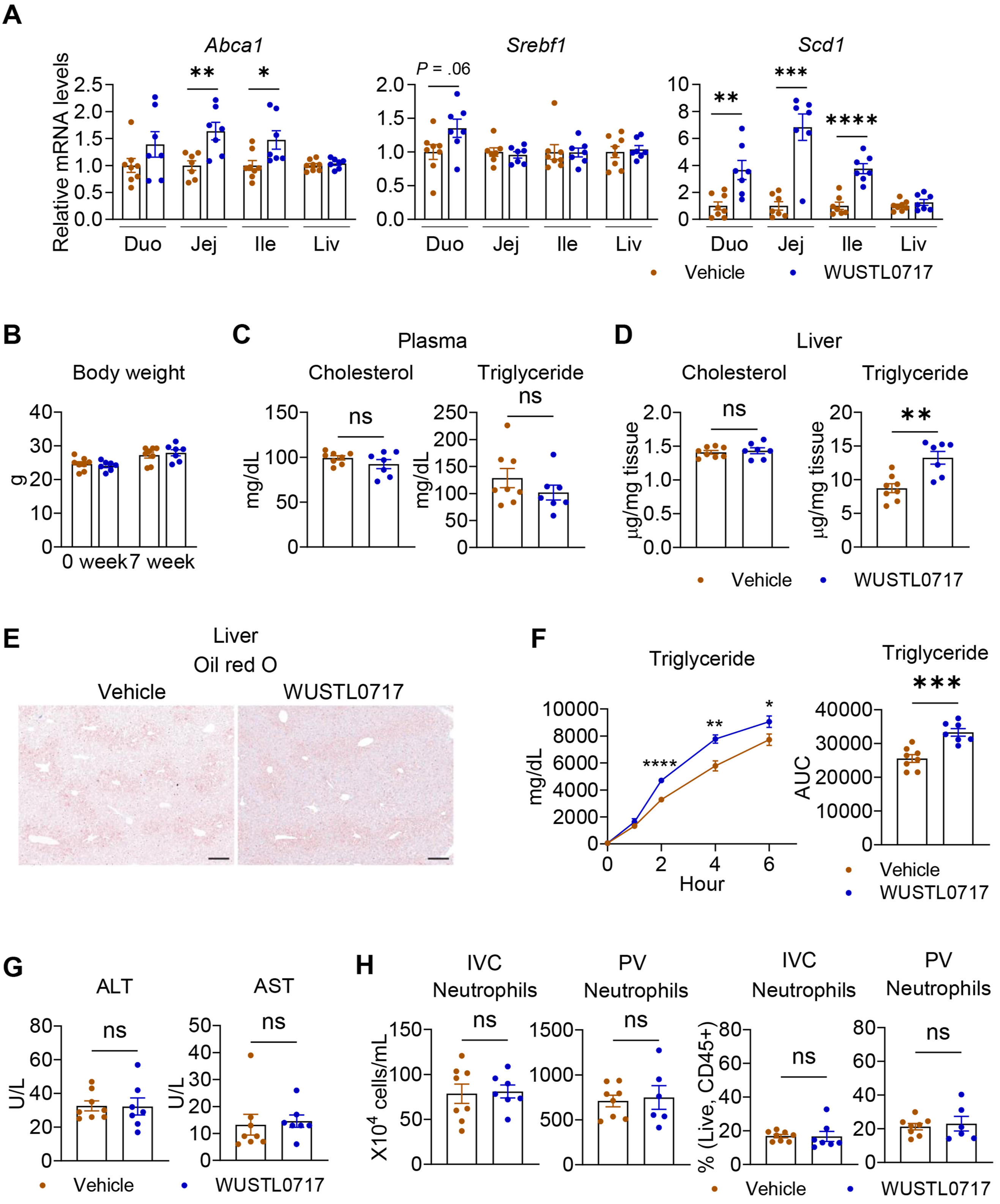

Figure 4. WUSTL0717 avoids metabolic dysfunction while promoting increased nutrient absorption. WT mice were treated with vehicle or WUSTL0717 (30 mg/kg, PO) daily for 7 weeks (n = 7–8/group). (*A*) Transcript levels of LXR target genes in the duodenum (Duo), jejunum (Jej), ileum (Ile), and liver (Liv) analyzed by qRT-PCR. (*B*) Body weight at baseline and after 7 weeks of WUSTL0717 treatment. (*C*) Plasma cholesterol and triglyceride levels. (*D*) Liver cholesterol and triglyceride levels. (*E*) Oil red O staining in the liver (*scale bar*, 200 μm). The images shown are representative of images from 8 mice treated with vehicle, 7 mice treated with WUSTL0717. (*F*) Plasma triglyceride levels and area under the curve (AUC) in WUSTL0717-treated and vehicle groups, measured 9 days before euthanization at 0, 1, 2, 4, and 6 hours after a 10-μL/kg olive oil gavage preceded by a 500-mg/kg intravenous Tyloxapol pretreatment. (*G*) Plasma ALT and AST activities. (*H*) Systemic (inferior vena cava [IVC]) and portal (PV) venous plasma neutrophils were measured by flow cytometry. Unpaired Student *t* test was applied for statistical evaluation.

HEPATOBILIARY

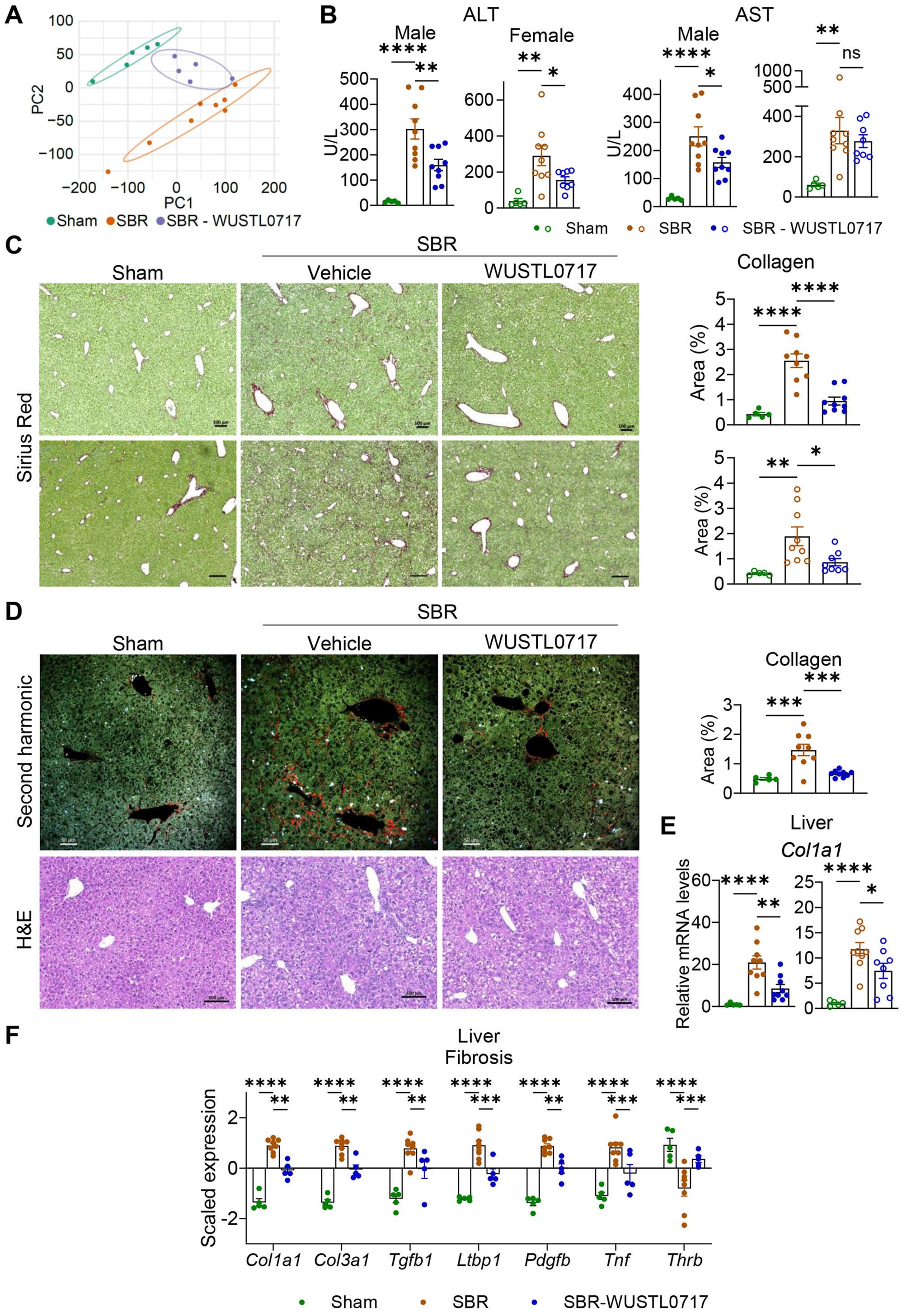

Figure 5. Protection against SBS-induced liver fibrosis through intestine-restricted LXR activation. WT male (*filled circles*) or female (*open circles*) mice underwent sham or SBR operation. Beginning 3 weeks post-surgery, the mice received either vehicle or WUSTL0717 (30 mg/kg, PO) daily for 7 weeks and were then euthanized for analysis (n = 5–9/group). (*A*) Principal component analysis of liver transcriptomes from the indicated groups. (*B*) ALT and AST from males (plasma) and females (serum). (*C*) Sirius Red–stained liver sections (upper: male; lower: female) with quantification from 5 areas per mouse, plotted as *individual dots* (*scale bar*, 100 μm). (*D*) Second-harmonic generation (SHG)-based collagen detection with quantification from 6 to 12 different areas per mouse, shown as *individual dots*, and representative hematoxylin-eosin (H&E)–stained liver sections. *Scale bars*: 50 μm (SHG), 100 μm (H&E). (*E*) *Col1a1* transcript levels in the liver analyzed by qRT-PCR. (*F*) Differentially expressed genes associated with collagen accumulation, hepatic stellate cell activation, and cytokine-related pathways in the liver. Statistical evaluations used 1-way analysis of variance (ANOVA) (*B*, *E*; Dunnett's test), 1-way ANOVA (*C* and *D*; Tukey's honestly significant difference [HSD]), and 2-way ANOVA (*F*; Tukey's HSD).

decreasing *Cyp8b1*, and these changes were reversed by WUSTL0717, which also upregulated *Abcb11*, suggesting a restoration of normal bile secretion (Supplementary Figure 5*A* and *B*). Antioxidant genes were upregulated by WUSTL0717 (Supplementary Figure 5*C*). Gene set variation analysis confirmed that collagen accumulation and extracellular matrix remodeling pathways, significantly upregulated after SBR, were downregulated by WUSTL0717 (Supplementary Figure 5*D*). The top-ranked downregulated liver genes after SBR were often restored in the presence of WUSTL0717 and included numerous major urinary protein family members (Supplementary Figure 5*E*), whose

downregulation is implicated in metabolic liver disease.[32] The top genes upregulated by SBR—including collagen genes and *Spp1* (*Osteopontin*), often produced by macrophages and associated with fibrosis[33]—were decreased by WUSTL0717 (Supplementary Figure 5*E*). These data demonstrate that WUSTL0717 acts in the intestine and robustly reduces liver fibrosis after SBR.

Phospholipids in the Portal Vein Correlate With the Protective Effects of the Gut-Restricted Liver X Receptor Agonist WUSTL0717 in Short Bowel Syndrome

We earlier concluded that intestinal HDL that travels in the portal vein is central to protecting the liver from IFALD.[8] This past work focused on eliminating intestinal epithelial cell expression of ABCA1,[8] a key transporter of cholesterol and phospholipids packaged by HDL. Indeed, phospholipids are largely dependent on lipoproteins for transport in plasma and HDL is the dominant lipoprotein in mice.[14,15,34] When we herein carried out lipidomic and metabolite profiling of the portal vein samples in our experimental cohorts, a clear distinction was revealed between sham and SBR groups, as demonstrated by principal component analysis (Figure 6*A*). SBR led to a marked reduction in numerous lipid classes, while a few were elevated (Figure 6*B*). Treatment with WUSTL0717 produced an overall modest shift (Figure 6*A*). Closer examination revealed that glycerophospholipids were significantly decreased in SBR and partially restored or increased to sham levels with WUSTL0717 treatment (Figure 6*B*). To further characterize treatment-specific changes, we performed statistical filtering based on $\log_2$ fold-change and adjusted *P* value between the SBR and SBR-WUSTL0717 groups, identifying 49 significantly altered metabolites. Glycerophospholipids again emerged as the predominant class, as illustrated in the pie chart, with PC and phosphatidylethanolamine (PE) being the most represented sub-classes within this group (Figure 6*C* and *D*). Correlation analysis within this subset revealed that both PC and ether-linked PC (PC O-), as well as PE and ether-linked PE (PE O-), were each inversely correlated with liver collagen accumulation (Figure 6*E*). Thus, WUSTL0717 increases several phospholipids in the portal vein, an outcome possibly linked to the protective effects of WUSTL0717 against liver fibrosis after SBR.

Intestinal Apoa1 and High-density Lipoprotein Cholesterol Protect Against Liver Injury Following Small Bowel Resection

Because HDL would be expected to be the major carrier of phospholipids in mouse plasma,[15] we hypothesized that intestine-derived ApoA1 itself would be important for hepatic protection, as ApoA1 and phospholipids together support HDL particle integrity.[34] In mice receiving SBR, WUSTL0717 administration enriched the cholesterol efflux gene ontology in both the post-anastomosis ileum and the duodenum (Figure 7*A*), but *Apoa1* transcript levels increased only in the post-anastomosis ileum (Figure 7*B*), underscoring the region-specific impact of WUSTL0717 on intestinal gene expression. After WUSTL0717 administration, portal venous HDL-C levels and ApoA1 levels were elevated in both sexes (Figure 7*C* and *D*). In sham-operated mice, systemic HDL-C exceeded portal venous HDL-C, consistent with previous reports,[8] but SBR reversed this relationship, lowering systemic HDL-C below portal venous levels (Figure 7*C* and Supplementary Figure 6*A*). WUSTL0717 treatment elevated HDL-C in both the portal and systemic veins (Figure 7*C* and Supplementary Figure 6*A*), partially reversing the suppression caused by SBR. Portal venous HDL-C and ApoA1 levels were inversely correlated with liver collagen accumulation (Figure 7*E* and *F*). ApoA1 was also detected in the ileal mucus and increased after WUSTL0717 in male mice (Supplementary Figure 6*B* and *C*). Given its presence in the mucus and the concept that HDL in the mucus might act as an antimicrobial agent or microbiome modifier,[35] we examined whether mucosal ApoA1 levels were associated with liver fibrosis; however, no correlation was observed (Supplementary Figure 6*D* and *E*). Fecal 16S ribosomal RNA sequencing revealed that the microbiome was stable before surgery but diverged across groups 10 weeks after surgery (Supplementary Figure 7*A*–*C*). Alpha diversity index increased after SBR compared with sham, and WUSTL0717 did not alter this increase, whereas WUSTL0717 treatment shifted beta diversity between the sham and SBR groups (Supplementary Figure 7*A* and *B*). Notably, the abundance of *Akkermansia*—a bacterium known to ameliorate metabolic dysfunction–associated fatty liver disease and liver fibrosis[36]—was markedly reduced by SBR but restored with WUSTL0717 treatment (Supplementary Figure 7*C*–*E*). However, neither liver collagen area nor portal venous ApoA1 levels correlated with *Akkermansia* abundance (Supplementary Figure 7*F*). These data suggest that the impact of WUSTL0717, through alterations in portal venous contents, is more pertinent to hepatic protection than changes in the microbiome or mucus.

To test the hypothesis that intestinal ApoA1 is required for liver protection, SBR was performed on mice lacking *Apoa1* in intestinal epithelial cells (*Apoa1*$^{\Delta IEC}$). In these mice, portal venous HDL-C levels were further reduced compared with the already low levels in WT SBR mice (Figure 7*G*), as was portal venous ApoA1 (Figure 7*H*). In contrast, serum ALT levels were elevated (Figure 7*I*), and loss of intestinal ApoA1 also increased markers of liver fibrosis, including type I collagen messenger RNA (Figure 7J) and Sirius Red staining (Figure 7*K*). The extent of fibrotic area was inversely correlated with portal venous HDL-C and ApoA1 (Figure 7*L* and *M*). We conclude that portal venous HDL-C and ApoA1 protect the liver from injury after SBR, and that the elevation of HDL in the gut-liver axis achieved by WUSTL0717 treatment mitigates IFALD.

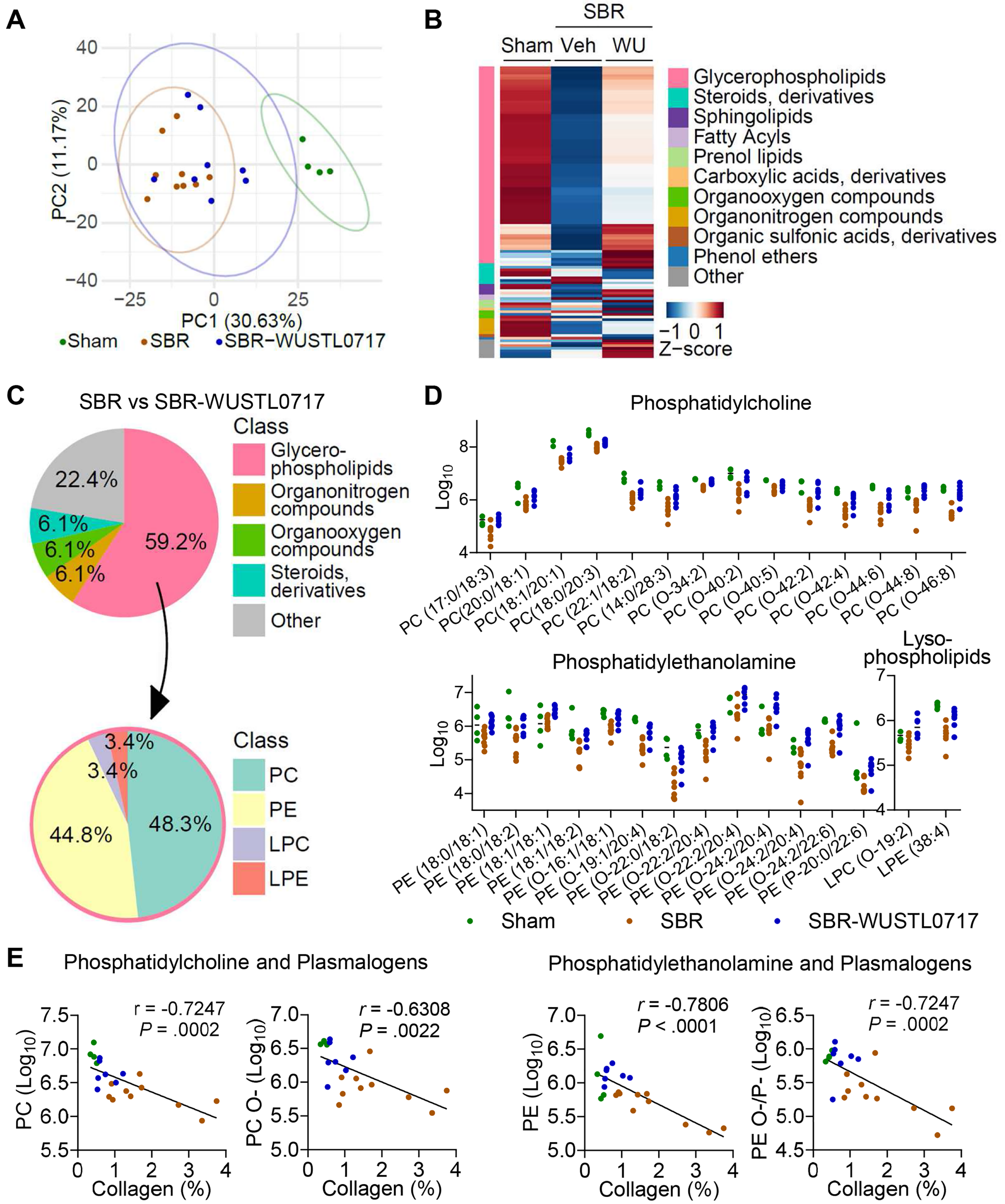

Figure 6. Association of portal venous phospholipids with the hepatoprotective effects of the gut-restricted LXR agonist after SBR. Lipid and metabolite profiling of portal venous serum using LC-MS/MS in WT female mice with sham or SBR surgery. Three weeks after surgery, mice received vehicle or WUSTL0717 for 7 weeks (n = 4–9/group). (*A*) Principal component analysis of overall metabolites. (*B*) Heatmap of metabolites with significant differences between SBR vs SBR-WUSTL0717 and sham vs SBR. (*C–E*) Analysis of portal venous serum metabolites significant at false discovery rate < 0.1 and fold change > 1.5, focusing on glycerophospholipids and their association with liver fibrosis. (*C*) Pie charts of metabolite classes (*left*) and the sub-classes of glycerophospholipids (*right*). (*D*) Individual glycerophospholipid metabolites from the sub-classes in (*C*) (*right*). PE (O-22:2/20:4) and PE (O-24:2/20:4) are each represented twice as isomeric species. (*E*) Correlation of portal venous serum PC, PC O-, PE, PE O-, and plasmenyl PE (PE P-) with liver collagen area (Figure 5*C*, lower). Each *dot* represents a matched individual from (*D*). Pearson correlation.

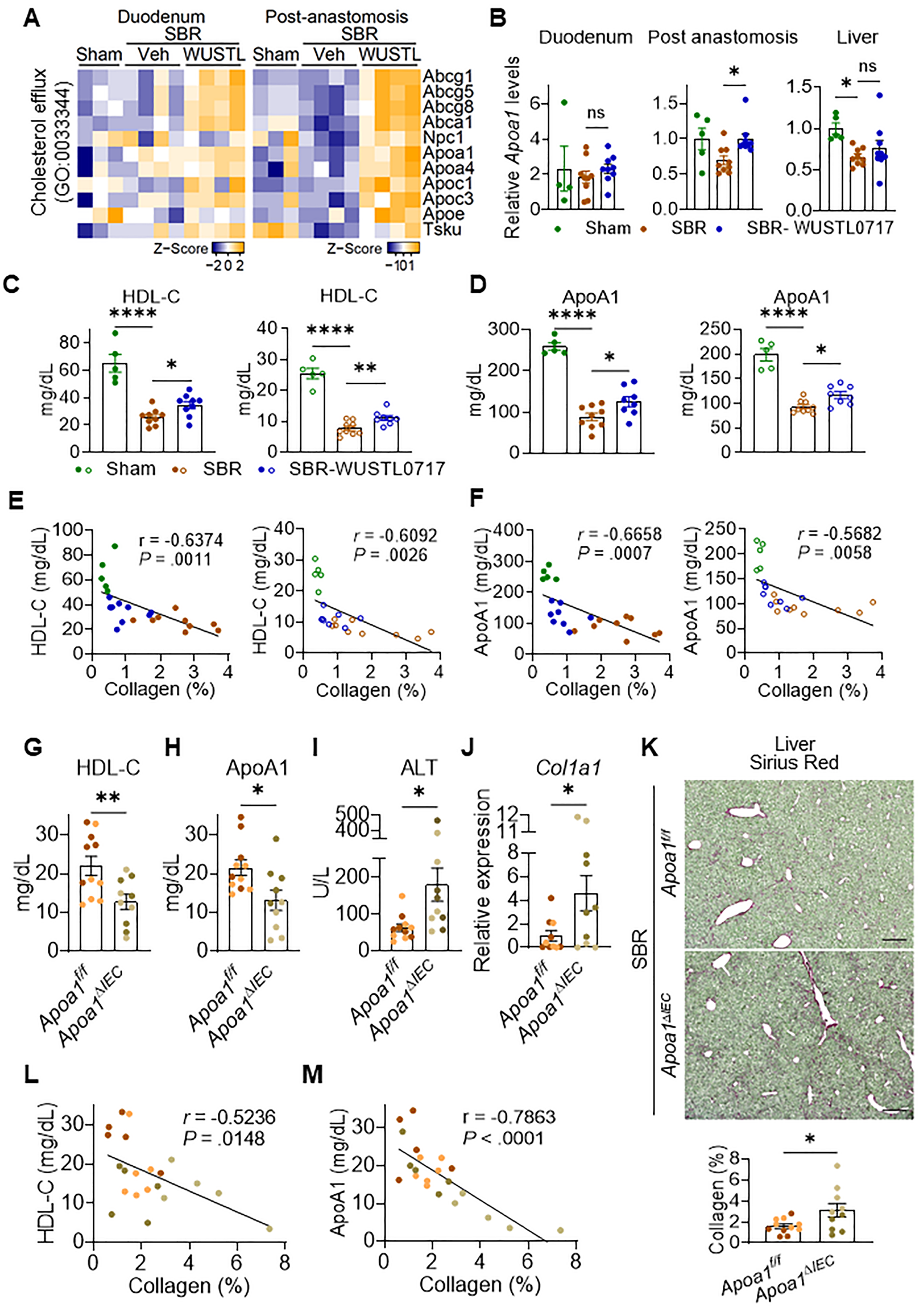

Figure 7. Aggravated IFALD following the deficiency of intestinal ApoA1 and HDL-C after SBR. (*A–D*) WT male (*filled circles*) or female (*open circles*) mice underwent sham or SBR. Starting 3 weeks later, mice received daily vehicle or WUSTL0717 (30 mg/kg, PO) for 7 weeks, followed by analysis (n = 5–9/group). (*A*) Heatmap of cholesterol efflux–related transcripts in the duodenum and post-anastomosis ileum from male mice (n = 3–4/group). (*B*) qRT-PCR analysis of *Apoa1* transcripts in the duodenum, post-anastomosis ileum, and liver (n = 4–9/group). (*C* and *D*) Portal venous HDL-C (*C*) and ApoA1 (*D*) levels from males (plasma) and females (serum) (n = 5–9/group). (*E* and *F*) Correlation of portal venous plasma HDL-C and ApoA1 levels with liver collagen area (Figure 5*C*, *upper*). Each *dot* represents a matched individual from (*C*) and (*D*). (*G–M*) *Apoa1*$^{\Delta IEC}$ mice underwent SBR and were euthanized 11 weeks later. *Dark dots* represent males and *light dots* represent females (males, n = 7/group; females: n = 6–7/group). (*G* and *H*) Portal venous serum HDL-C and ApoA1 levels. (*I*) Systemic (inferior vena cava) serum ALT levels. (*J*) Liver *Col1a1* transcript levels analyzed by qRT-PCR. (*K*) Sirius Red–stained liver sections (*scale bar,* 200 μm) with quantification from 5 to 6 areas per liver, 1 image per mouse (males, n = 7/group; females: n = 6–7/group). (*L* and *M*) Correlation of portal venous serum HDL-C and ApoA1 levels with liver collagen area. Each *dot* represents a matched individual from (*K*)–(*M*). Statistical evaluations were done using unpaired Student *t* test (*C*, *G–K*), 1-way analysis of variance with Tukey's honestly significant difference (*B*, *D*), or Pearson correlation (*E*, *F*, *L*, and *M*).

Discussion

Building on our previous work linking intestine-derived HDL to liver injury via the portal vein,[8,37] we explored the therapeutic potential of LXR agonists with activity confined to the intestine. Although systemic LXR agonists failed clinically due to hepatic lipogenesis,[29] the sole report of GW6340 as a putative gut-restricted LXR agonist[13] seemed a promising alternative, but the report lacked pharmacokinetic evaluation, preventing rigorous evaluation of its intestinal restriction and functional impact. To address the lack of information on gut-restricted LXR agonists, we synthesized GW6340, described in the patent PCT/US01/27622, and named it WUSTL0717. Pharmacokinetic and molecular analyses confirmed its intestinal restriction, showing LXR targets induction in the intestine but not in the liver. The amide structure of WUSTL0717 is central to its gut-restricted activity. Compared with the carboxylic acid of GW3965[9]—the sole structural difference between the 2 compounds—the amide remains largely nonionized at intestinal pH and is less polar and more metabolically stable, collectively limiting systemic uptake and promoting gut luminal retention. In addition, the high molecular weight (581.078 g/mol), 13 rotatable bonds, and low kinetic solubility (0.29 mM) of WUSTL0717 fall outside the optimal range associated with high oral bioavailability.[38] Because WUSTL0717 must enter intestinal epithelial cells to activate LXR, it could theoretically exit these cells to mobilize systemically via lymphatics or blood,[39] but our pharmacokinetic studies detected minimal trafficking from the intestine. It seems likely that, in addition to its nonpolar character, robust catabolism may also contribute to its confinement to the intestine.

WUSTL0717 enhances transcription of lipid metabolic genes and improves chylomicron secretion, conferring metabolic benefit by fostering weight regain after SBR, likely via proximal intestinal action. In unoperated mice, WUSTL0717 modestly increased chylomicron secretion, without obvious negative metabolic effects, suggesting neutrality in health but clear benefits after SBR. This adaptive effect may relate to a recently reported role of LXR signaling in promoting intestinal stem cell–driven regeneration and tissue adaptation after damage.[40] That LXR agonists can promote intestinal epithelial health without promoting tumorigenesis bodes well for translational potential of targeting LXR in a gut-restricted manner. Indeed, intestinal remodeling capacity is considered critical in the context of SBS, as impaired adaptation contributes to nutritional deficiency and hepatic complications.[1] IFALD from SBS can be severe, sometimes necessitating a liver transplant, yet no standard therapy exists. We propose that the application of gut-restricted LXR agonists may be particularly valuable following substantial resection, creating a window for preventive intervention. In our model, WUSTL0717 protected the liver by attenuating fibrosis, inflammation, and cholestasis while restoring bile secretion, antioxidant pathways, and *Thrb* expression. Notably, a thyroid hormone receptor beta agonist was recently approved by the Food and Drug Administration for liver fibrosis.[30] WUSTL0717 also modestly altered some host defense–related genes, with the functional significance of these changes requiring further study.

Our findings suggest that the phospholipids restored by WUSTL0717 likely originate from the intestine[41] and reflect elevated portal venous ApoA1 and HDL that, in turn, support hepatoprotection. Higher portal phospholipid levels correlated with reduced fibrosis, supported by studies of phosphatidylcholine supplementation reducing liver fibrosis.[42] However, whether these phospholipids themselves protect the liver via HDL delivery, by altering cargo-affiliated HDL, or directly affect hepatic function remains unknown. We show that expression of ApoA1 by the intestine is, like enterocyte-derived ABCA1,[8] important in quelling IFALD. Although ApoA1, the protein backbone of HDL, was elevated in ileal mucus[35] by WUSTL0717, its presence in mucus did not correlate with liver fibrosis. Similarly, the fecal microbiome, comparable across the mice before surgery, diverged markedly by 10 weeks. *Akkermansia muciniphila*, a microbe considered beneficial,[36] was reduced after SBR but restored with WUSTL0717 treatment. However, these changes did not correlate with liver fibrosis outcomes, suggesting a secondary rather than a primary protective mechanism. In contrast, portal ApoA1 and HDL strongly correlated inversely with fibrosis, and intestinal *Apoa1* deletion worsened liver outcomes, underscoring that gut-derived HDL protects the liver primarily through the portal circulation. In conclusion, our characterization of WUSTL0717 as a gut-restricted LXR agonist that modulates portal HDL biogenesis and lipid handling supports future evaluation of WUSTL0717 in large animal models to translationally assess whether gut-restricted LXR agonists may be therapeutically viable to reduce intestine-driven liver disease.

Supplementary Material

Note: To access the supplementary material accompanying this article, visit the online version of *Gastroenterology* at www.gastrojournal.org, and at https://doi.org/10.1053/j.gastro.20110.1053/j.gastro.2025.12.015.

References

1. Goulet O, Ruemmele F. Causes and management of intestinal failure in children. Gastroenterology 2006;130:S16–S28.
2. Abu-Wasel B, Molinari M. Liver disease secondary to intestinal failure. Biomed Res Int 2014;2014:968357.
3. Seiler KM, Waye SE, Kong W, et al. Single-cell analysis reveals regional reprogramming during adaptation to massive small bowel resection in mice. Cell Mol Gastroenterol Hepatol 2019;8:407–426.
4. Kelly DA. Intestinal failure-associated liver disease: what do we know today? Gastroenterology 2006;130:S70–S77.

5. Lam HS, Tam YH, Poon TC, et al. A double-blind randomised controlled trial of fish oil-based versus soy-based lipid preparations in the treatment of infants with parenteral nutrition-associated cholestasis. Neonatology 2014;105:290–296.
6. Fligor SC, Tsikis ST, Hirsch TI, et al. A medium-chain fatty acid analogue prevents intestinal failure-associated liver disease in preterm Yorkshire piglets. Gastroenterology 2023;165:733–745.e9.
7. Peet DJ, Janowski BA, Mangelsdorf DJ. The LXRs: a new class of oxysterol receptors. Curr Opin Genet Dev 1998;8:571–575.
8. Han Y-H, Onufer EJ, Huang L-H, et al. Enterically derived high-density lipoprotein restrains liver injury through the portal vein. Science 2021;373:eabe6729.
9. Leik CE, Carson NL, Hennan JK, et al. GW3965, a synthetic liver X receptor (LXR) agonist, reduces angiotensin II-mediated pressor responses in Sprague-Dawley rats. Br J Pharmacol 2007;151:450–456.
10. Schultz JR, Tu H, Luk A, et al. Role of LXRs in control of lipogenesis. Genes Dev 2000;14:2831–2838.
11. Chisholm JW, Hong J, Mills SA, et al. The LXR ligand T0901317 induces severe lipogenesis in the db/db diabetic mouse. J Lipid Res 2003;44:2039–2048.
12. Collins JL, Fivush AM, Watson MA, et al. Identification of a nonsteroidal liver X receptor agonist through parallel array synthesis of tertiary amines. J Med Chem 2002;45:1963–1966.
13. Yasuda T, Grillot D, Billheimer JT, et al. Tissue-specific liver X receptor activation promotes macrophage reverse cholesterol transport in vivo. Arterioscler Thromb Vasc Biol 2010;30:781–786.
14. Kontush A, Lhomme M, Chapman MJ. Unraveling the complexities of the HDL lipidome. J Lipid Res 2013;54:2950–2963.
15. Dashti M, Kulik W, Hoek F, et al. A phospholipidomic analysis of all defined human plasma lipoproteins. Sci Rep 2011;1:139.
16. Tsujita M, Vaisman B, Chengyu L, et al. Apolipoprotein A-I in mouse cerebrospinal fluid derives from the liver and intestine via plasma high-density lipoproteins assembled by ABCA1 and LCAT. FEBS Lett 2021;595:773–788.
17. Washburn DG, Hoang TH, Campobasso N, et al. Synthesis and SAR of potent LXR agonists containing an indole pharmacophore. Bioorg Med Chem Lett 2009;19:1097–1100.
18. Courtney CM, Warner BW. Pediatric intestinal failure-associated liver disease. Curr Opin Pediatr 2017;29:363–370.
19. Wang Y, Yu Y, Li L, et al. Bile acid-dependent transcription factors and chromatin accessibility determine regional heterogeneity of intestinal antimicrobial peptides. Nat Commun 2023;14:5093.
20. Thompson CA, Wojta K, Pulakanti K, et al. GATA4 is sufficient to establish jejunal versus ileal identity in the small intestine. Cell Mol Gastroenterol Hepatol 2017;3:422–446.
21. Lampropoulou V, Sergushichev A, Bambouskova M, et al. Itaconate links inhibition of succinate dehydrogenase with macrophage metabolic remodeling and regulation of inflammation. Cell Metab 2016;24:158–166.
22. Platell CF, Coster J, McCauley RD, et al. The management of patients with the short bowel syndrome. World J Gastroenterol 2002;8:13–20.
23. Bannert K, Karbe C, Förster RH, et al. Orally compensated short bowel patients are thin, potentially malnourished but rarely sarcopenic. Clin Nutr 2023;42:1480–1490.
24. Hickey JW, Becker WR, Nevins SA, et al. Organization of the human intestine at single-cell resolution. Nature 2023;619:572–584.
25. Onufer EJ, Han YH, Czepielewski RS, et al. Effects of high-fat diet on liver injury after small bowel resection. J Pediatr Surg 2020;55:1099–1106.
26. Wang B, Rong X, Duerr MA, et al. Intestinal phospholipid remodeling is required for dietary-lipid uptake and survival on a high-fat diet. Cell Metab 2016;23:492–504.
27. Onufer EJ, Czepielewski R, Seiler KM, et al. Lymphatic network remodeling after small bowel resection. J Pediatr Surg 2019;54:1239–1244.
28. Lin X, Yue P, Chen Z, et al. Hepatic triglyceride contents are genetically determined in mice: results of a strain survey. Am J Physiol Gastrointest Liver Physiol 2005;288:G1179–G1189.
29. Kirchgessner TG, Sleph P, Ostrowski J, et al. Beneficial and adverse effects of an LXR Agonist on human lipid and lipoprotein metabolism and circulating neutrophils. Cell Metab 2016;24:223–233.
30. Keam SJ. Resmetirom: first approval. Drugs 2024;84:729–735.
31. Sakellariou S, Michaelides C, Voulgaris T, et al. Keratin 7 expression in hepatic cholestatic diseases. Virchows Arch 2021;479:815–824.
32. Gao R, Wang H, Li T, et al. Secreted MUP1 that reduced under ER stress attenuates ER stress induced insulin resistance through suppressing protein synthesis in hepatocytes. Pharmacol Res 2023;187:106585.
33. Zhu C, Kim K, Wang X, et al. Hepatocyte Notch activation induces liver fibrosis in nonalcoholic steatohepatitis. Sci Transl Med 2018;10:eaat0344.
34. Brunham LR, Kruit JK, Iqbal J, et al. Intestinal ABCA1 directly contributes to HDL biogenesis in vivo. J Clin Invest 2006;116:1052–1062.
35. Mukherjee P, Chattopadhyay A, Grijalva V, et al. Oxidized phospholipids cause changes in jejunum mucus that induce dysbiosis and systemic inflammation. J Lipid Res 2022;63:100153.
36. Yan J, Sheng L, Li H. Akkermansia muciniphila: is it the Holy Grail for ameliorating metabolic diseases? Gut Microbes 2021;13:1984104.
37. Han YH, Onufer EJ, Huang LH, et al. Enterically derived high-density lipoprotein restrains liver injury through the portal vein. Science 2021;373:eabe6729.
38. Dahan A, Miller JM. The solubility-permeability interplay and its implications in formulation design and development for poorly soluble drugs. AAPS J 2012;14:244–251.
39. Randolph GJ, Miller NE. Lymphatic transport of high-density lipoproteins and chylomicrons. J Clin Invest 2014;124:929–935.

40. Das S, Parigi SM, Luo X, et al. Liver X receptor unlinks intestinal regeneration and tumorigenesis. Nature 2025;637:1198–1206.
41. Carneiro C, Brito J, Bilreiro C, et al. All about portal vein: a pictorial display to anatomy, variants and physiopathology. Insights Imaging 2019;10:38.
42. Aleynik SI, Leo MA, Ma X, et al. Polyenylphosphatidylcholine prevents carbon tetrachloride-induced lipid peroxidation while it attenuates liver fibrosis. J Hepatol 1997;27:554–561.

Received February 10, 2025. Accepted December 3, 2025.

Correspondence
Address correspondence to: Gwendalyn J. Randolph, PhD, Department of Pathology and Immunology, 425 S. Euclid Avenue, Campus Box 8118-86-10, Saint Louis, Missouri 63105. e-mail: gjrandolph@wustl.edu.

Acknowledgments
This study is dedicated to the memory of coauthor Brad W. Warner, who passed away during the final phases of its completion and whose career paved the way for modeling SBS experimentally. We are grateful to Washington University School of Medicine affiliates Mary Wohltmann and Chunxiao Shi for expert help in maintaining and genotyping mice for this study, Ji-Sun Kwon for her expert training and assistance in analyzing RNA-seq data, and Giljae Lee for his expert assistance with 16S analysis. We appreciate the advice and role of Danny Griffin (Biogenerator, St Louis) in the origins of this project. We thank Maki Tsujita (Nagoya City University) and Alan Remaley (National Institutes of Health) for *Apoa1*$^{f/f}$ mice. The article's graphical abstract was created using BioRender.com.

CRediT Authorship Contributions
Ayoung Kim, PhD (Conceptualization: Lead; Data curation: Lead; Formal analysis: Lead; Funding acquisition: Equal; Investigation: Lead; Visualization: Lead; Writing original draft: Lead; Writing – review & editing: Supporting; First Author: Lead)
Daniel M. Alligood, MD (Data curation: Supporting; Funding acquisition: Equal; Investigation: Equal; Methodology: Equal; Writing – review & editing: Supporting)
Lingaiah Maram, PhD (Investigation: Supporting; Resources: Equal)
Hannah M. Phelps, MD (Data curation: Supporting; Investigation: Equal; Methodology: Equal; Writing – review & editing: Supporting)
Michael Cameron, PhD (Data curation: Equal; Formal analysis: Equal; Funding acquisition: Equal; Investigation: Equal)
Jacob T. DeRousse, PhD (Data curation: Equal; Formal analysis: Equal; Investigation: Equal)
Jichang Han, PhD (Formal analysis: Equal; Visualization: Supporting; Writing – review & editing: Supporting)
Taylor J. Dunning, BA (Investigation: Supporting)
Rachel L. Mintz, BA (Investigation: Supporting; Writing – review & editing: Supporting)
Alex Park, MD (Investigation: Supporting; Methodology: Supporting)
Daniel D. Lee, PhD (Investigation: Supporting)
Christopher Huckstep, BS (Investigation: Supporting; Writing – review & editing: Supporting)
Deanna D. Davis, PhD (Investigation: Supporting; Writing – review & editing: Supporting)
Rachael L. Field, BA (Data curation: Equal; Formal analysis: Equal; Investigation: Equal)
Lamees Hegazy, PhD (Data curation: Supporting; Formal analysis: Supporting; Resources: Supporting)
Bernd H. Zinselmeyer, PhD (Data curation: Equal; Methodology: Equal; Resources: Equal)
Jonathan R. Brestoff, PhD (Data curation: Supporting; Formal analysis: Supporting; Funding acquisition: Equal; Investigation: Supporting)
Colin Martin, MD (Supervision: Supporting; Writing – review & editing: Supporting)
Brad W. Warner, MD[†] (Funding acquisition: Equal; Resources: Equal; Writing – review & editing: Equal)
Bahaa Elgendy, PhD (Conceptualization: Lead; Formal analysis: Equal; Funding acquisition: Equal; Resources: Lead; Supervision: Lead; Writing – original draft: Equal; Writing – review & editing: Equal)
Gwendalyn J. Randolph, PhD (Conceptualization: Lead; Formal analysis: Equal; Funding acquisition: Lead; Project administration: Lead; Resources: Lead; Supervision: Lead; Writing – original draft: Equal; Writing – review & editing: Lead)

Conflicts of interest
These authors disclose the following: Ayoung Kim, Brad W. Warner,[†] Bahaa Elgendy, and Gwendalyn J. Randolph are part of an intellectual property claim for the use of intestinal LXR agonists to treat SBS, US Patent Application No. 18/997, 728 entitled "Compositions for the Treatment of Intestinal Failure and Use Thereof." The remaining authors disclose no conflicts.

Funding
This research was primarily supported by National Institutes of Health (NIH) R01DK119147 to Gwendalyn J. Randolph, Brad W. Warner,[†] and Bahaa Elgendy, with additional support from NIH grants R01AI168044 and U01AI63064 to Gwendalyn J. Randolph. Ayoung Kim received support from T32AI007163 and T32AR007279, and Daniel M. Alligood received support from T32DK077653. Jonathan R. Brestoff received support from R01NS134932. Mass spectrometry was supported by NIH grant S10OD030332 to Michael Cameron. Additional support for this research included core facilities at Washington University School of Medicine: the Diabetes Research Core (NIH P30 DK020579), the Digestive Diseases Research Core Center (NIH P30 DK052574), the Center for Cellular Imaging supported by the Children's Discovery Institute (CDI-CORE-2015-505 and CDI-CORE-2019-813), and the Foundation for Barnes-Jewish Hospital (3770 and 4642), the Musculoskeletal Research Center (NIH P30 AR074992), and the Division of Comparative Medicine Research Animal Diagnostic Laboratory. Genomic and transcriptomic dataset generation was supported by the Genome Technology Access Center, funded by the National Cancer Institute (NCI) Cancer Center Support Grant (CCSG) (P30 CA91842) and the Institute of Clinical and Translational Sciences (ICTS)/National Center for Advancing Translational Sciences (NCATS) Clinical and Translational Science Awards (CTSA) Award (UL1 TR002345). Lipid and metabolite profiling was supported by the Mass Spectrometry Technology Access Center at the McDonnell Genome Institute, with additional support from the Diabetes Research Center (P30 DK020579), ICTS/NCATS CTSA Award (UL1 TR002345), and Siteman Cancer Center/NCI CCSG Grant (P30 CA091842).

Data Availability
The sequencing and expression data have been deposited in the Gene Expression Omnibus of the National Center for Biotechnology Information under accession code GSE287046. On publication, all data will be accessible through the Open Science Framework. The corresponding author also may be contacted as needed to clarify any concerns around data or resource access.

Gastroenterology, Volume *170*

Supplemental information

A Gut-Restricted Liver X Receptor Agonist Ameliorates Liver Injury in Experimental Short Bowel Syndrome

Ayoung Kim, Daniel M. Alligood, Lingaiah Maram, Hannah M. Phelps, Michael Cameron, Jacob T. DeRousse, Jichang Han, Taylor J. Dunning, Rachel L. Mintz, Alex Park, Daniel D. Lee, Deanna L. Davis, Christopher G. Huckstep, Rachael L. Field, Lamees Hegazy, Bernd H. Zinselmeyer, Jonathan R. Brestoff, Colin A. Martin, Brad W. Warner, Bahaa Elgendy, and Gwendalyn J. Randolph

Supplementary Materials and Methods

Molecular modeling

WUSTL0717 and GW3965 were modeled in LXRα protein from reference structure PDBID: 3IPU and based on the coordinates of GW3965 in Xray structure 1PQ6.[1, 2] WUSTL0717 was modeled in LXRβ based on the coordinates of GW3965 in Xray structure 1PQ6. Charges were modeled using AM1BCC, energy minimization using Amber ff14SB, Steepest descent,[3] and conjugate gradient algorithms[4] implemented in UCSF Chimera.[5]

Ligand binding in LanthaScreen TR-FRET LXRβ coactivator assays

Binding assays of graded concentrations of WUSTL0717 or GW3965 to LXRβ were performed using the LanthaScreen TR-FRET LXRβ Coactivator kit (Thermo Fisher Scientific) following the manufacturer's protocol. Briefly, after the addition of 5 nM LXRβ-LBD to the compounds, a mixture of peptide (100 nM, Fluorescein-D22 for LXRβ) and antibody (10 nM, Tb-anti-GST) was added to the reaction followed by incubation in room temperature for 4 hours. TR-FRET ratio was calculated by dividing the emission at 520 nm by the emission at 495 nm using either Synergy 2 (BioTek) or FlexStation 3 (Molecular Devices) plate reader. The TR-FRET ratio was then normalized by dividing the ratio of each concentration by the averaged ratio of the positive control, which was set equal to 100%. Concentration-response curves were fitted using 4-variable non-linear regression.

Activation of transcriptional functions of LXRα and LXRβ

Plasmids LXR-α (79514; Addgene), LXR-β (79513; Addgene), and LXRE_Luc (177622; Addgene) were transfected into HEK293T cells (ATCC CRL-3216) using the Lipofectamine™ 3000 kit (L3000001; Thermo Fisher Scientific). 24 hours post-transfection, the cells were plated in a 96-well plate at a density of 30,000 cells per well. After an additional 24 hours, the cells were treated with WUSTL0717 or GW3965 for another 24 hours. Luciferase activity was then measured using a Synergy HTX (BioTek) plate reader with the ONE-Glo™ luciferase assay kit (E8130; Promega) at room temperature. Concentration-response curves were fitted using 4-variable non-linear regression.

Kinetic Solubility

Compound from a 10 mM DMSO stock solution was introduced to pre-warmed pH 7.4 phosphate buffered saline in a 96-well plate with a final DMSO concentration of 1%. The plate was maintained at 37°C for 24 hours on an orbital shaker and centrifuged through a Millipore Multiscreen Solvinter 0.45 micron low binding PTFE hydrophilic filter plate and analyzed by HPLC. Peak area was compared to standards of known concentration.

Plasma protein binding

Plasma protein binding was determined using equilibrium dialysis. All samples were tested in triplicate using the RED Rapid Equilibrium Dialysis Device (Thermo Fisher Scientific). The initial drug concentration in the plasma chamber was 2 µM, and phosphate buffered saline was added to the receiver chamber. The plate was covered and allowed to shake in a 37ºC incubator for 6 hours. 25 µl was sampled from the plasma and PBS chambers, which were then diluted with either blank PBS or plasma to achieve a 1:1 ratio or plasma:PBS for all samples. The concentration of the drug in the plasma and PBS chambers was determined by LC-MS/MS. The fraction bound was calculated as ([plasma] – [PBS]) / [plasma].

Hepatic microsomal stability

Microsome stability was evaluated by incubating 1 µM test compound with 1 mg/mL hepatic microsomes in 100 mM KPi, pH 7.4. The reaction was initiated by adding NADPH (1 mM final concentration). Aliquots were removed at 0, 5, 10, 20, 40, and 60 minutes and added to acetonitrile (5X, v:v) to stop the reaction and precipitate the protein. NADPH dependence of the reaction was evaluated by setting up incubations without NADPH. At the end of the assay, the samples were centrifuged through a Millipore Multiscreen Solvinter 0.45 micron low binding PTFE hydrophilic filter plate and analyzed by LC-MS/MS. Data were log-transformed and represented as half-life. The microsomes were purchased from B1oIVT. Human were av200 donor pool/mixed sex.

Metabolic cages

Mice were weighed, and their core body temperature was measured using a rectal thermometer. Body composition was assessed using EchoMRI-100H 2n1 (EchoMRI). Afterwards, the mice were housed individually in Comprehensive Laboratory Animal Monitoring System (CLAMS) metabolic cages (Columbus Instruments) for one day of acclimation, followed by 24 hours for recording. The cages were contained within a light-sealed environmental enclosure with LED white light strips next to each cage, which were turned on at 6:00am (Zeitbeber Time 0) and turned off at 6:00pm (Zeitgeber Time 12) each day for a 12h:12h light:dark cycle that matches that of the standard animal facility. The enclosure temperature was set a 22.2 °C. The airflow rate was set at 0.90 L/min, with an 18-second bleed of the air sampling tube followed by a 2-second measurement period for each cage, with sampling of enclosure air in each interval. Energy expenditure (heat) and RER were calculated from measurements of the volume of oxygen consumption and carbon dioxide production using indirect calorimetry with a zirconia O_2 sensor and CO_2 sensors. Ambulatory activity was measured using infrared laser detector arrays positioned along the X- and Y-axes at animal height.

qRT-PCR assays

Total RNA from tissues was extracted using Direct-zol RNA Miniprep Kits (R2052; Zymo Research) and was reverse-transcribed into cDNA using the High-Capacity cDNA Reverse Transcription Kit (4368814; Thermo Fisher Scientific). cDNA was amplified either TaqMan Fast Advanced Master Mix (4444554; Thermo Fisher Scientific) with the following probes: *Abca1* (Mm00442646_m1), *Srebf1* (Mm00550338_m1), *Scd1* (Mm00772290_m1), *Apoa1* (Mm00437569_m1), *Actb* (Mm02619580_g1), or *18S* (Hs99999901_s1), or SYBR Green qRT-PCR Master Mix (A46110; Thermo Fisher) with primer sequences listed in Supplementary Table 4. qRT-PCR was conducted using QuantStudio 6 Pro (Applied Biosystems).

Preparation of ileal mucosal protein for ApoA1 ELISA

Mucus collection was performed as previously described[6] with minor modifications. After flushing the small intestine with PBS, a 2-cm segment of ileum was dissected, opened lengthwise and pinned flat. Any remaining luminal contents were gently flushed with additional PBS rinses. The mucosal surface was subsequently rinsed with 2 mL of 10 mM Tris-HCl (pH 7.4) containing 5 mM $CaCl_2$, followed by gentle scraping using a cell lifter (08-100-240; Fisher Scientific). The collected mucus was transferred into 500 μL of lysis buffer [50 mM Tris-HCl (pH 7.5), 100 mM NaCl, 5 mM EDTA, 1% Triton X-100, and protease inhibitor]. The lysate was centrifuged twice at 13,000 rpm for 10 min to remove debris, and the final supernatant was stored at −20°C. Total protein was quantified by the Bradford assay, and 1:15 diluted samples were analyzed for ApoA1 levels using an ELISA kit (3750-1HP; Mabtech).

Intestinal lipid absorption and chylomicron secretion assay

Mice that were 9 to 11 weeks post-SBR with 6 to 8 weeks of WUSTL0717 administration, as well as non-operated mice that had received 6 weeks of WUSTL0717 treatment, underwent an intestinal triglyceride absorption assay. Mice were fasted in the dark cycle for 8–11 hours, then injected *i.v.* with 500 mg/kg body weight of Tyloxapol (T0307; Sigma-Aldrich) and then 10 μl/g body weight of olive oil (O1514; Sigma-Aldrich) via oral gavage one hour later. Blood was collected from tail veins at baseline and at 1, 2, 4, and 6 hours. Plasma triglycerides were measured using the L-Type TG-H kit (Wako Chemicals) read using a Cytation 5 Cell Imaging Multi-Mode Reader (BioTek).

Glucose uptake assay

Mice were 13 weeks post-SBR and had received 10 weeks of WUSTL0717 treatment before being fasted overnight for 15 hours. They were then administered a glucose solution (G8270; Sigma-Aldrich) via oral gavage at a dose of 2 g/kg body weight. Blood glucose levels were determined from the tail veins at baseline and 15, 30, 60, and 120 minutes after glucose injection using GLUCOCARD Vital (760001; Arkray).

Flow cytometry

Cells from blood stained with acridine orange were counted using an automated cell counter (Cellometer Auto T4; Nexelcom Bioscience). Red blood cells were lysed using BD Pharm Lyse (555899; BD Biosciences) for 8 minutes at room temperature, followed by centrifugation at 500 × g for 5 minutes. The lysis step was repeated 2–3 times as needed. The remaining cells were resuspended in PBS for 30 minutes. The cells were then stained with Live Zombie NiR (423106; Biolegends) in PBS for an additional 30 minutes to exclude dead cells. Next, the cells were stained with antibodies against cell surface markers for 30 minutes in FACs buffer consists of 2% FBS, 2 mM EDTA, 0.02% sodium azide, and 20% Brilliant buffer (563794; BD Biosciences) in PBS. Conjugated antibodies against CD45 (568336) and CD11b (612801) were sourced from BD Biosciences, and Ly6G to identify neutrophils was sourced from BioLegend (127641). Cells were acquired using the 5-lasers, 64-channels Cytek Aurora spectral flow cytometer (Cytek Biosciences) equipped with SpectroFlo software (version 3.1.0) and analyzed with FlowJo v10. All steps involving Live Zombie NiR staining, antibody staining, and fixation were performed on ice and in the dark.

Histological analysis

Liver and small intestine tissues were fixed in 4% paraformaldehyde, embedded in paraffin, and sectioned at a thickness of 4 µm. The sections were mounted on slides and stained with Hematoxylin & Eosin (H&E) for morphology analysis or with Sirius Red to assess collagen accumulation. Alternatively, 8-µm thick cryosections of snap-frozen liver samples were mounted on slides and stained with Oil Red O through Washington University Musculoskeletal Research Center. Slides were imaged using Zeiss Axio Scan Z1 (Zeiss) and analyzed using Zen and Fiji-ImageJ software. For the analysis of SHG, deparaffinized liver tissue slides were subjected to 2-photon microscopy. Two-photon microscopy images were collected using a customized dual-laser system (InSight® & Mai Tai®, Spectra-Physics) on a Leica SP8 upright microscope equipped with a 25x, 0.95 numerical aperture water-immersion objective. The Mai Tai® laser was tuned to 920 nm, and signal separation was achieved using three long-pass dichroic beam splitters (FF640-FDi01, FF562-FDi03, and FF495-Di03, Semrock) to generate channels at approximately 390–495 nm (SHG), 495–562 nm, and 562–640 nm. Fluorescence emission was directed to external hybrid photodetectors (Leica). Analysis of the second harmonic signal was carried out using Imaris software in combination with Fiji-ImageJ software.

RNA sequencing

Total RNA integrity was determined using an Agilent Bioanalyzer or 4200 Tapestation. Library preparation was performed with 500 ng to 1 µg of total RNA. Ribosomal RNA was removed using an RNase H method with RiboErase kits (Kapa Biosystems). mRNA was then fragmented in reverse transcriptase buffer by heating to 94°C for 8 minutes. The fragmented mRNA was reverse transcribed to yield cDNA using SuperScript III RT enzyme (Life Technologies) and random hexamers. A second-strand reaction was performed to yield double-stranded cDNA. cDNA was blunt-ended, had an A base added to the 3' ends, and Illumina sequencing adapters were ligated to the ends. Ligated fragments were then amplified for 12–15 cycles using primers incorporating unique dual index tags. Fragments were sequenced on an Illumina NovaSeq X Plus using paired-end reads extending 150 bases. Base calling and demultiplexing were performed with Illumina's bcl2fastq software with a maximum of one mismatch in the indexing read. RNA-seq reads were then aligned to the Ensembl release 101 primary assembly with STAR version 2.7.9a1. Gene counts were derived from the number of uniquely aligned unambiguous reads by Subread:featureCounts (version 2.0.32). Sequencing performance was assessed for the total number of aligned reads, the total number of uniquely aligned reads, and features detected. The ribosomal fraction, known junction saturation, and read distribution over known gene models were quantified with RSeQC version 4.04, and RNA-seq was conducted by the Genome Technology Access Center. All gene counts were then imported into the R/Bioconductor package EdgeR5, and TMM normalization size factors were calculated to adjust for differences in library size across samples. For downstream analysis, the TPM files were directly loaded into R for further analysis. Gene names were standardized, and duplicate entries were removed to retain unique gene features. Genes with zero TPM values across all samples were excluded. To reduce the impact of extreme values and facilitate comparisons, the TPM data were log2-transformed. Batch effects between experimental groups were

corrected using the ComBat function from the sva R package. Normalized data were visualized with boxplots to confirm consistent sample distributions and ensure comparability across datasets. Filtered TPM matrices were then subjected to downstream analyses. DEGs were identified by comparing experimental groups. Log_2FC and p-values were calculated for each gene using unpaired two-sample t-tests and Wilcoxon rank-sum tests. Genes were ranked by log_2FC and p-values, and the Benjamini–Hochberg procedure was used to control the false discovery rate (FDR). Genes with an adjusted *P*-value < .05 and an absolute log_2FC above a set threshold were considered significantly differentially expressed. Preranked GSEA was performed using the GSEA_4.0.3 with MSigDB collections. Significant enrichment was determined by normalized enrichment scores (NES) and adjusted FDR q-values <0.05. GSVA scores were calculated across samples and standardized (z-score normalization) using custom gene sets, including those associated with LXR signaling, which were defined using the GSEABase package. PCA, heatmaps, enrichment plots, and volcano plots, were generated using R (ggplot2, gplots, dplyr, EnhancedVolcano).

16S rRNA sequencing

DNA was extracted using the ZymoBIOMICS®-96 MagBead DNA Kit (Zymo Research). Library preparation for targeted 16S sequencing was performed using the Quick-16S™ Plus NGS Library Prep Kit with V3–V4 primers. Amplification was performed using a qPCR–based method to minimize chimera formation. PCR products were quantified, pooled at equal molarity, and purified. Final libraries were quantified and sequenced on an Illumina® NextSeq 2000™ (600-cycle P1 kit). Positive controls (ZymoBIOMICS® Microbial Community Standard or DNA Standard) and negative controls (blank extraction and library controls) were included to monitor contamination. Sequencing reads were processed with the DADA2 pipeline for denoising and chimera removal. Taxonomy was assigned using UCLUST from QIIME v.1.9.1 with the Zymo Research 16S reference database. Diversity and composition analyses were conducted using QIIME v.1.9.1, and Linear Discriminant Analysis Effect Size (LEfSe) was used to identify taxa with significant group differences. Sequencing and analysis were performed by Zymo Research.

Lipid and metabolite extraction and LC-MS/MS Analysis

Portal venous serum from female mice was collected. Sample preparation and LC-MS/MS for lipid and metabolite profiling were subsequently performed by the Mass Spectrometry Technology Access Center at the McDonnell Genome Institute. Samples were subjected to biphasic extraction using methyl tert-butyl ether. Samples were vortexed 3 times and incubated at –80°C for 1 hour. After phase separation, the upper phase containing lipids was collected and dried using a SpeedVac without applying heat. The lower phase containing polar metabolites was centrifuged, and the resulting supernatant was similarly dried. Dried lipid extracts were reconstituted in 19 μL of methanol:acetonitrile:water (2:1:1, v/v/v), and dried metabolite extracts were reconstituted in 19 μL of 50% methanol. Reconstituted samples were analyzed by LC-MS/MS using a Vanquish Horizon UHPLC system (Thermo Fisher Scientific) equipped with C8 and C18 columns, and coupled to an Orbitrap Tribrid ID-X mass spectrometer (Thermo Fisher Scientific) operating in both positive and negative ion modes with the AcquireX DeepScan workflow. In total, 2,293 features were annotated as metabolites and lipid species. Quantification was performed based on MS1 peak areas (AUC). Imputed data were used for downstream analyses, and statistical analysis and plot generation were performed using R. For PCA, the imputed data were processed by log_{10} transformation, interquartile range filtering at 40%, sum normalization, and autoscaling. Metabolites with an FDR < 0.2 and an FC > 1.5 in SBR-WUSTL0717 compared to SBR were log_{10}-transformed and used for heatmap generation. For generating pie charts and for analyses of individual significant metabolites, we followed the workflow typically used for volcano plots: data were processed by log_2 transformation and sum normalization without autoscaling, and significant metabolites were defined as those with an FDR < 0.1 and an FC > 1.5, according to the standard MetaboAnalyst (https://metaboanalyst.ca) workflow. Finally, the mean log_{10} value of each group was used to analyze the Pearson correlation with liver collagen accumulation.

References

1. Färnegårdh M, Bonn T, Sun S, et al. The three-dimensional structure of the liver X receptor beta reveals a flexible ligand-binding pocket that can accommodate fundamentally different ligands. J Biol Chem 2003;278:38821-8.
2. Fradera X, Vu D, Nimz O, et al. X-ray structures of the LXRalpha LBD in its homodimeric form and implications for heterodimer signaling. J Mol Biol 2010;399:120-32.
3. Meza JC. Steepest descent. WIREs Computational Statistics 2010;2:719-722.
4. Nazareth JL. Conjugate gradient method. WIREs Computational Statistics 2009;1:348-353.
5. Pettersen EF, Goddard TD, Huang CC, et al. UCSF Chimera--a visualization system for exploratory research and analysis. J Comput Chem 2004;25:1605-12.
6. Mukherjee P, Chattopadhyay A, Grijalva V, et al. Oxidized phospholipids cause changes in jejunum mucus that induce dysbiosis and systemic inflammation. J Lipid Res 2022;63:100153.

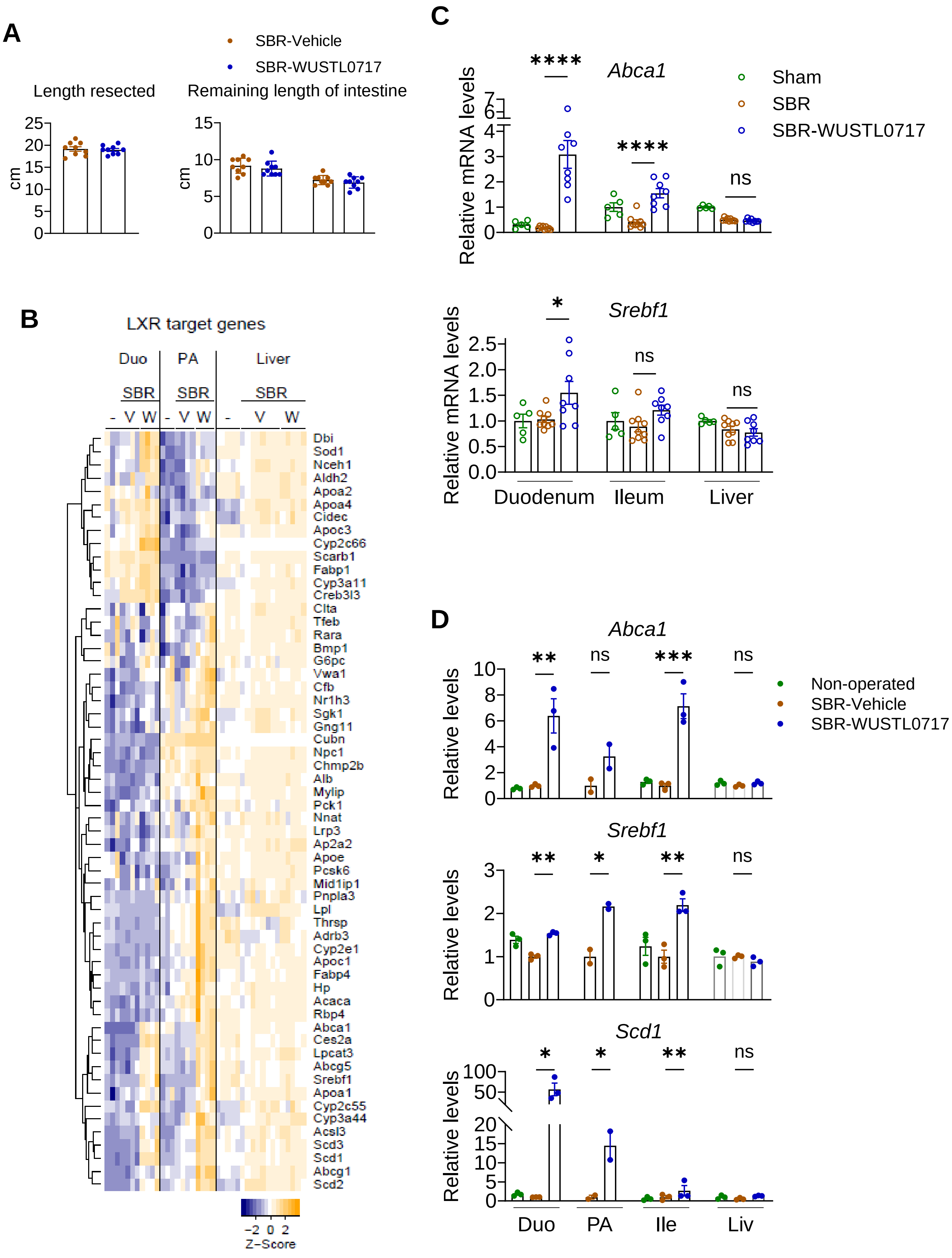

Supplementary Figure 1

Supplementary Figure 1. Intestine-restricted LXR agonist activity following treatment with WUSTL0717.

(A–C) WT male (filled circles) or female (open circles) mice underwent sham or SBR surgery. Starting 3 weeks post-surgery, the mice were treated daily with vehicle or WUSTL0717 (30 mg/kg, p.o.) for 7 weeks (n = 5–9/group).

(A) Length of the small intestine resected during SBR and remaining length at 10 weeks post-SBR.

(B) Heatmap showing LXR target gene expression in the duodenum (Duo), post-anastomosis ileum (PA), and liver (Liv), based on RNA-seq. V, vehicle; W, WUSTL0717.

(C) Transcript levels of LXR target genes in the duodenum, post-anastomosis ileum, or liver, measured by qRT-PCR.

(D) WT male mice (n = 2–3/group) underwent sham or SBR surgery and were treated with vehicle or WUSTL0717 starting 5 days post-surgery for 10 days before sacrifice.

Statistical analysis was performed using one-way ANOVA with Dunnett's (C) or Tukey's HSD (D) test for multiple comparisons. Data are presented as mean ± SEM; **P* < .05, ***P* < .01, ****P* < .001, *****P* < .0001; ns, not significant. Mean ± SEM and p-value thresholds are applied consistently across figures unless otherwise noted.

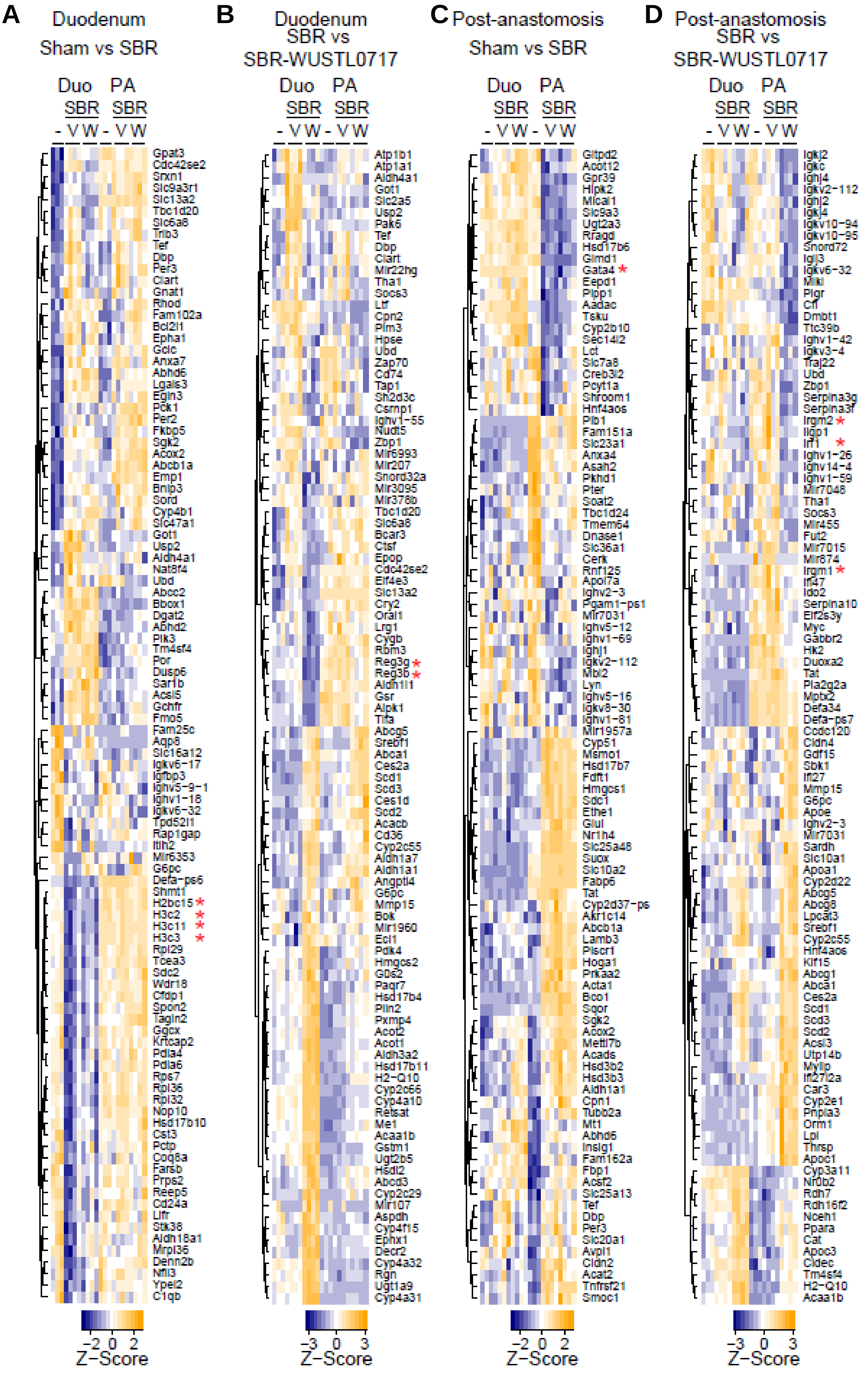

Supplementary Figure 2

Supplementary Figure 2. Intestinal adaptation following SBR and WUSTL0717 treatment.

(A–D) WT male mice underwent sham or SBR surgery. 3 weeks later, mice were treated daily with vehicle or WUSTL0717 (30 mg/kg, p.o.) for 7 weeks before euthanasia. Heatmaps show the top 50 DEGs ($p < 0.05$, FC ≥ 2) identified by RNA-seq in the duodenum (A, B) and post-anastomosis ileum (C, D). Comparisons are sham vs. SBR (A, C) and vehicle- (V) vs. WUSTL0717-treated (W) mice following SBR (B, D) (n = 3–4/group).

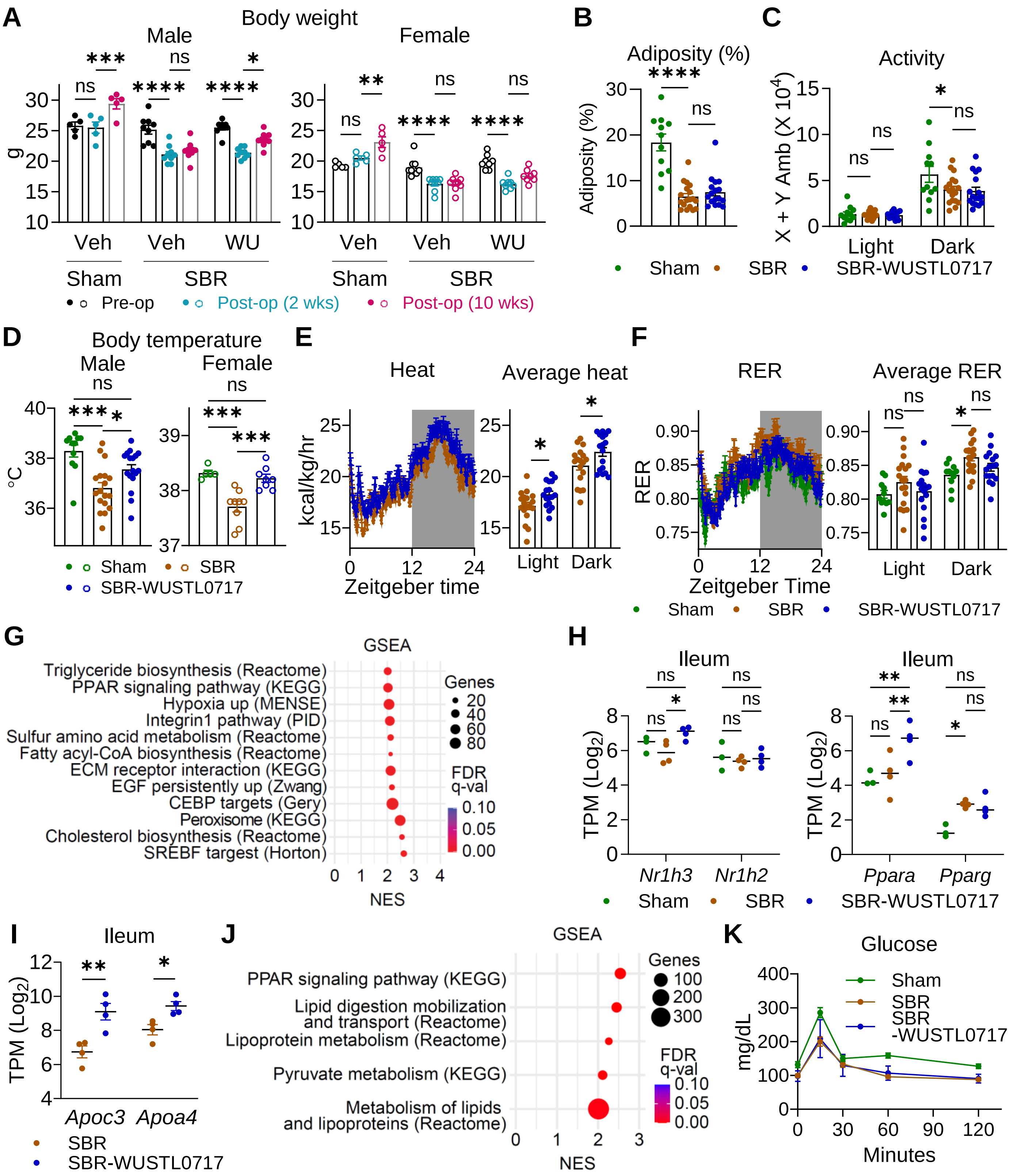

Supplementary Figure 3

Supplementary Figure 3. Metabolic phenotype of WUSTL0717 treatment in WT mice following SBR.

WT male (filled circles) or female (open circles) mice underwent sham or SBR surgery. Beginning 3 weeks post-surgery, mice received vehicle or WUSTL0717 (30 mg/kg, p.o.) daily for up to 7 weeks before euthanasia, unless otherwise specified (I). For the glucose tolerance test (I), treatment continued for 10 weeks, and measurements were performed at 13 weeks post-surgery.

(A) Body weight of mice measured at the indicated weeks (n = 5–9/group). Veh, vehicle; WU, WUSTL0717.

(B–F) At 8–9 weeks post-operation, mice treated with vehicle or WUSTL0717 for 5–6 weeks were subjected to adiposity measurement (B) and core body temperature assessment (D). Mice were individually housed in metabolic cages for a 24-hour evaluation of locomotor activity (C), heat generation (E) and RER (F). The time traces in panels E and F represent group mean values over time, while the adjacent bar plots show the corresponding light- and dark-phase averages for each individual mouse, with each dot representing one mouse. Data from male mice were combined from two independent experiments (male: n = 11–18 /group; female: n = 5–9/group).

(G, J) GSEA of RNA-seq data from post-anastomosis ileum, with enrichment plots illustrating normalized enrichment scores (NES) and associated gene signatures from GSEA of RNA-seq data. (G) Signatures upregulated in SBR relative to sham; (J) signatures upregulated in WUSTL0717-treated group compared to vehicle following SBR (n = 3–4/group).

(H, I) Transcript levels linked to proximal intestinal identity in the post-anastomosis ileum, analyzed by RNA-seq (n = 3–4/group).

(K) Blood glucose levels were measured in overnight-fasted mice 13 weeks post-surgery at 0, 15, 30, 60, and 120 minutes after a 2 g/kg glucose gavage (n = 8–11/group).

Unpaired Student’s t-test (E, I), one-way ANOVA (B, D), or two-way ANOVA with Tukey’s HSD (A, C, F, H, K) was used for statistical analysis.

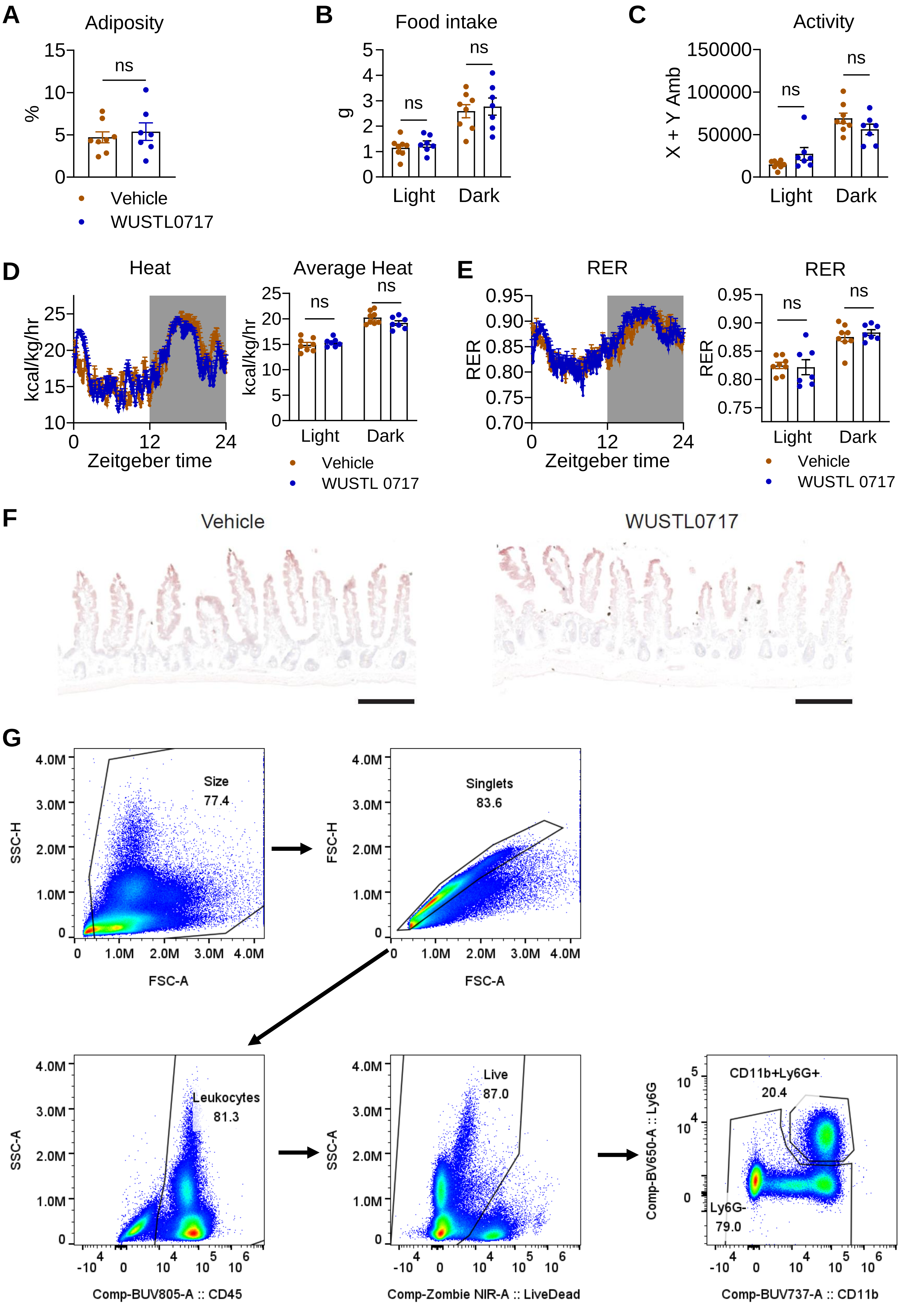

Supplementary Figure 4

Supplementary Figure 4. Metabolic phenotype of WUSTL0717 treatment in WT mice.

(A–G) WT male mice treated daily with either vehicle (n = 8) or WUSTL0717 (30 mg/kg, p.o.) (n = 7) for up to 7 weeks before euthanasia.

(A–E) After 5 weeks of treatment, mice underwent adiposity measurement (A) and were then individually housed in metabolic cages for 24-hour assessment of food intake (B), locomotor activity (C), heat generation (D), and RER (E) under a 12-hour light-dark cycle.

(F) Representative Oil Red O staining of the jejunum from each group after euthanasia (scale bar: 200 μm). Images shown are representative of evaluations from 8 vehicle- and 7 WUSTL0717-treated mice.

(G) Representative flow cytometry plots showing the gating strategy for blood cell suspensions collected after euthanasia, referenced to Figure 4H.

Unpaired Student's t-test was used for statistical evaluation.

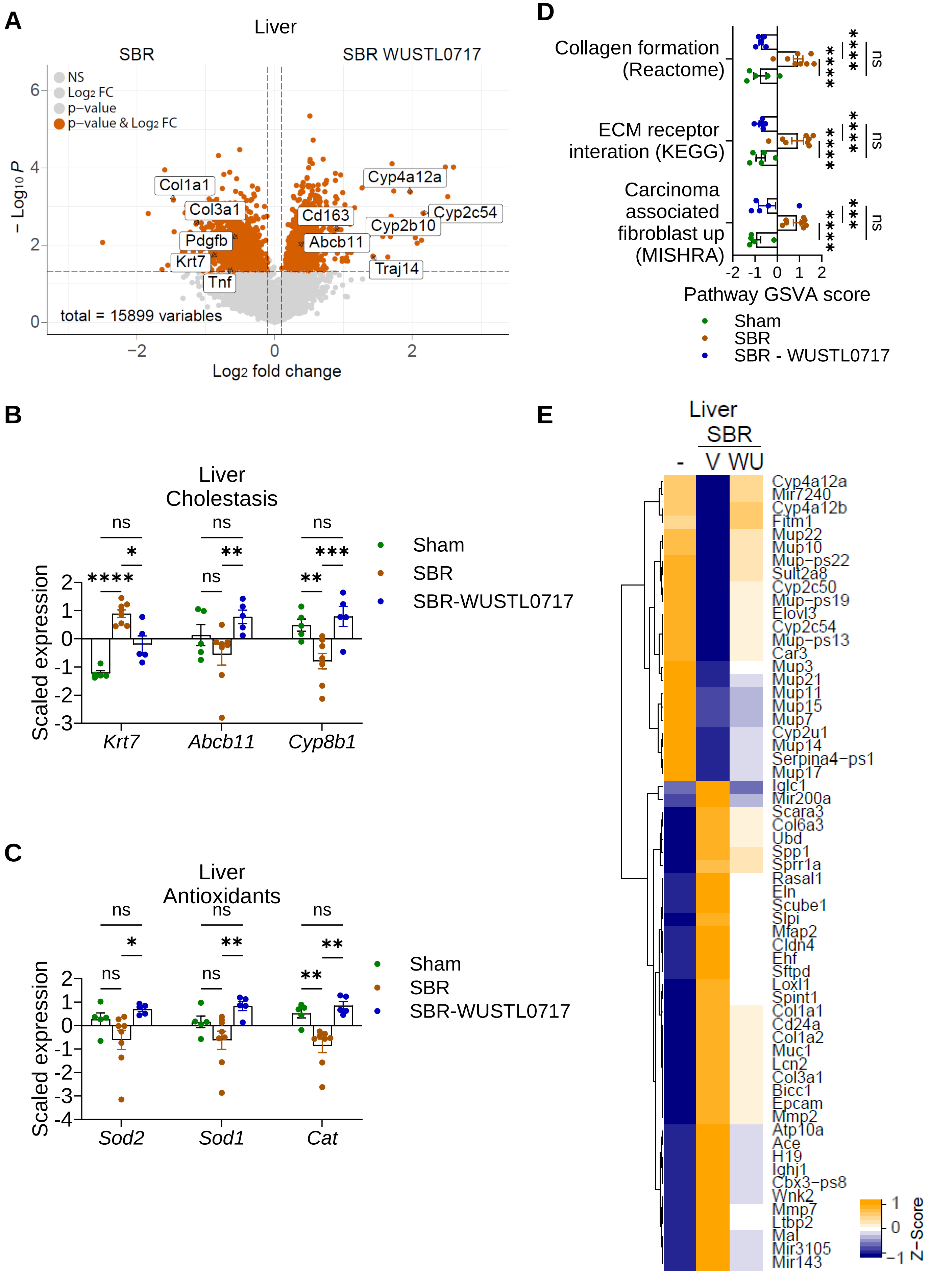

Supplementary Figure 5

Supplementary Figure 5. Analysis of DEGs associated with liver fibrosis in sham or SBR, WUSTL0717-treated groups.

(A, B, D–F) RNA-seq was performed on liver tissue from WT male mice that underwent sham or SBR surgery and were treated daily with vehicle or WUSTL0717 (30 mg/kg, p.o.) for 7 weeks (n = 5–8/group).

(A) Volcano plot showing DEGs in the liver between vehicle- and WUSTL0717-treated groups following SBR. DEGs were identified based on an adjusted p-value < 0.05 and $\log_2$FC thresholds, with significant genes highlighted in orange.

(B) Transcript levels of *Krt7, Abcb11*, and *Cyp8b1* in the liver.

(C) Transcript levels of *Sod2*, *Sod1*, and *Cat* in the liver.

(D) GSVA scores for liver fibrosis-related pathways.

(E) Heatmap of DEGs in the liver, showing the up- or downregulated genes with fold change (*P*-value < .05 and FC ≥ 2) when comparing the vehicle and WUSTL0717-treated groups following SBR. The heatmap also highlights genes with significant differences (*P*-value < .05) between sham and SBR groups. For visualization, a single group mean was normalized to a z-score and shown in the heatmap.

Statistical evaluations used two-way ANOVA (B–D) with Tukey's HSD.

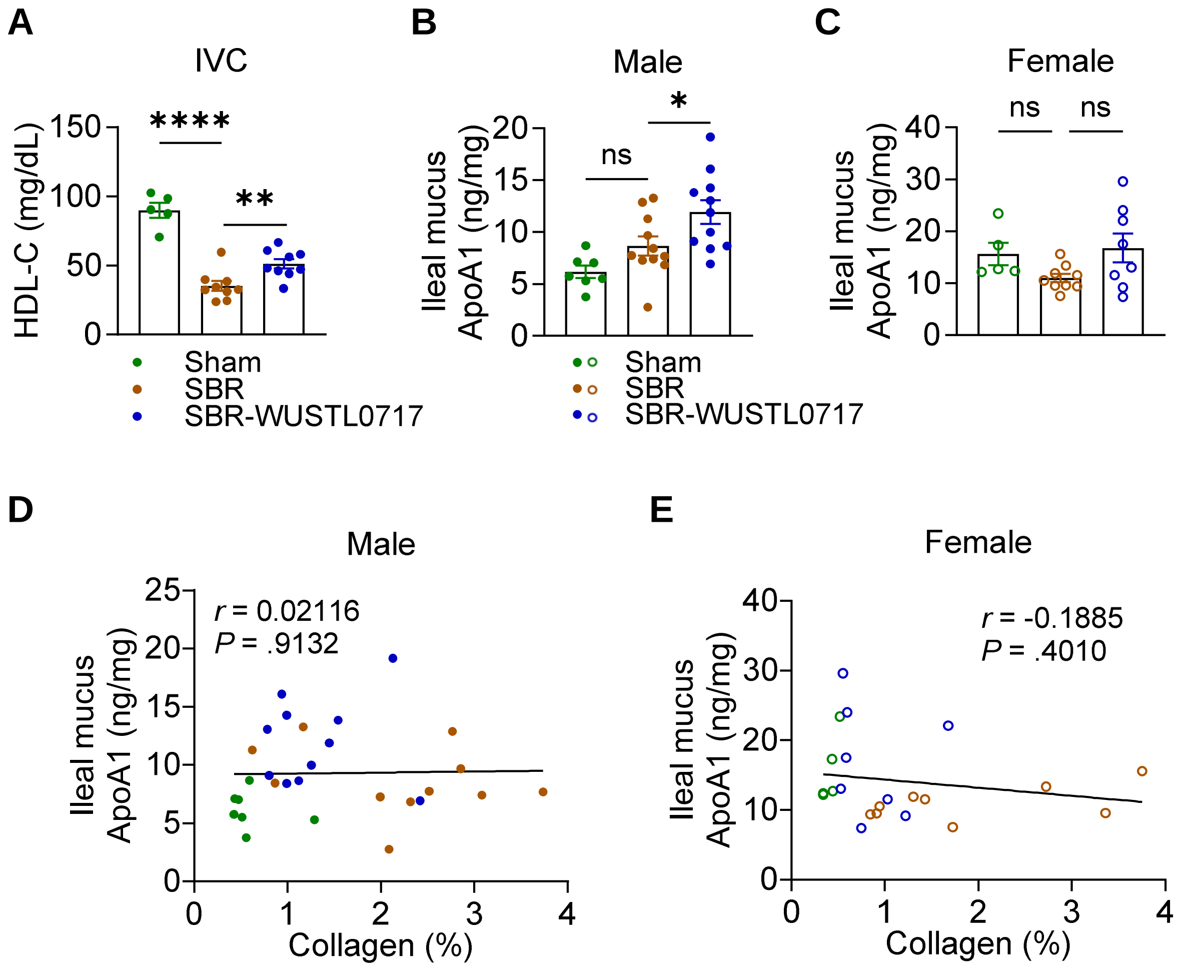

Supplementary Figure 6

Supplementary Figure 6. HDL-C and correlation of ileal mucus ApoA1 with liver fibrosis after WUSTL0717 treatment following SBR.

(A–E) WT male (filled circles) and female (open circles) mice underwent sham or SBR surgery. 3 weeks later, the mice received vehicle or WUSTL0717 (30 mg/kg, p.o.) daily for 7 (A, C, E) or 12 (B, D) weeks, after which they were euthanized for analysis (n = 5–11/group).

(A) Systemic plasma HDL-C levels (IVC) in each group.

(B, C) ApoA1 levels in the ileal mucus layer measured by ELISA and normalized to total protein content.

(D, E) Correlation between ileal mucosal ApoA1 levels (B, C) and liver collagen area (Figure 5C). Each dot represents a matched individual from panels B and C.

Statistical evaluations were performed using unpaired Student’s t-test (A), one-way ANOVA with Tukey’s HSD (B, C), or Pearson correlation (D-E).

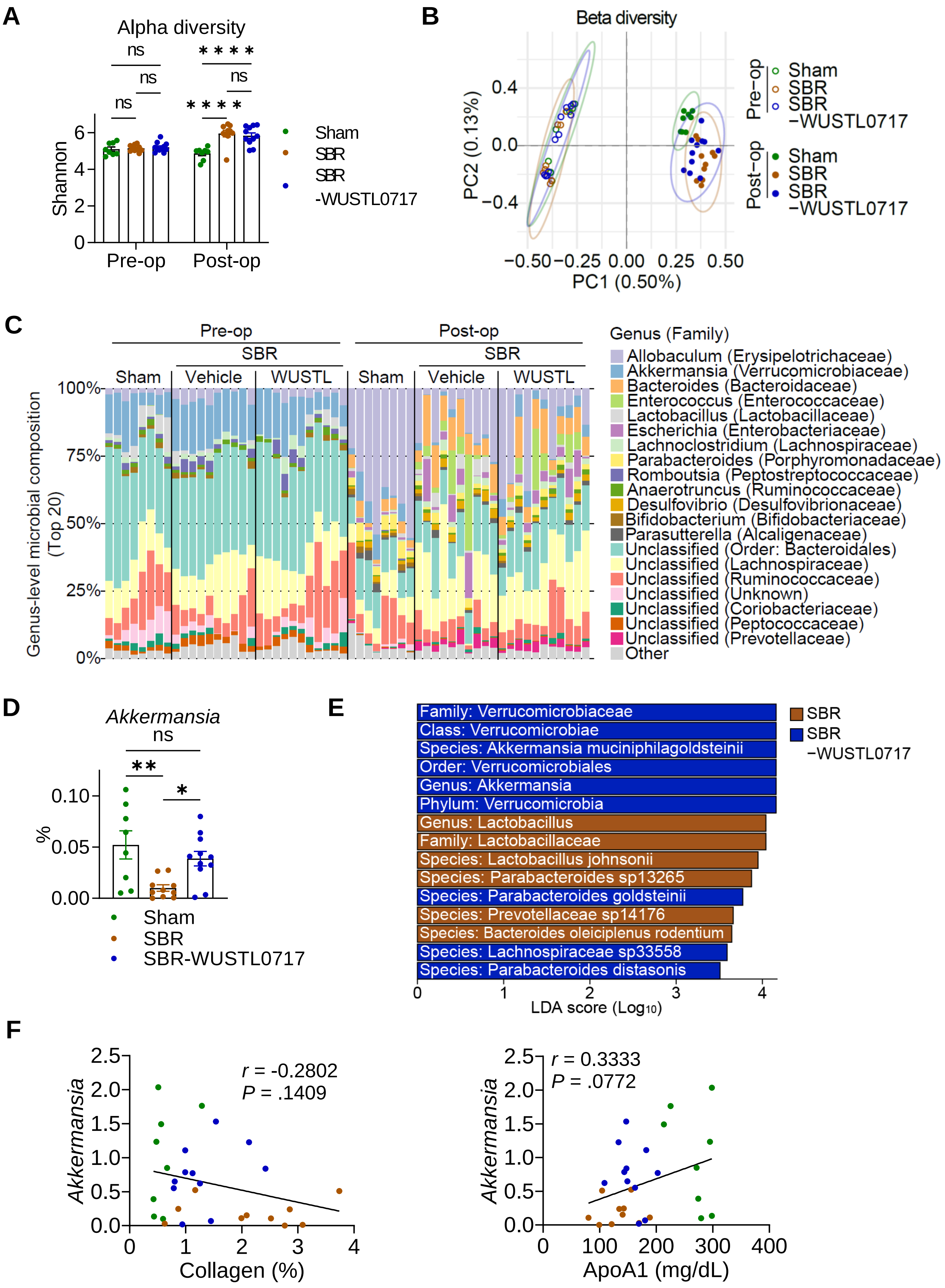

Supplementary Figure 7

Supplementary Figure 7. Fecal microbiome analysis of WUSTL0717-treated mice after SBR.

16S rRNA sequencing was performed on fecal samples from WT male mice collected before surgery and 10 weeks after sham or SBR surgery, following daily treatment with vehicle or WUSTL0717 (30 mg/kg, p.o.) for 7 weeks (n = 8–11/group).

(A) Shannon alpha diversity index.

(B) Bray–Curtis beta diversity index.

(C) Top 20 genera in the microbial composition. Genus and family information are shown in the figure for each group.

(D–F) Analysis of post-operative groups at 10 weeks after surgery.

(D) Percentage of *Akkermansia* in the total microbial composition.

(E) Taxa with LEfSe LDA scores > 3.5 are displayed on a log scale.

(F) Correlation between *Akkermansia* abundance (D) and either liver collagen area or portal venous plasma ApoA1 levels. Each dot represents an individual sample matched to panel D.

One-way ANOVA (D), two-way ANOVA (A) with Tukey's HSD, and Pearson correlation (F) were used for statistical analyses.

Supplementary Table 1. Genotyping primers (mouse)

Primer (mouse)	Forward	Reverse
Apoa1 flox	CGAAGTTATGAATTCTATCTCGCACCT	TGACCAGGATCCATAACTTCGTATAATGT
Apoa1 flox-WT	ACCGTGGATATCTCGCACCTT	TCTGACCAGTACTGGGGGTTA
Villin-Cre	GCCTTCTCCTCTAGGCTCGT	AGGCAAATTTTGGTGTACGG
		TATAGGGCAGAGCTGGAGGA (Internal primer)

Supplementary Table 2. WUSTL0717 instrument settings

LC (Shimadzu UFLC XR) conditions

Compound	WUSTL0717	I.S. (Carbamazepine)
Column	Thermo Betasil C18 5μ, 50x2.1mm	
Mobile phase	A: Water with 0.1% Formic Acid<br>B: Acetonitrile with 0.1% Formic Acid	
Flow rate (mL/minutes)	0.35	
Temperature (°C)	35	
Injection volume(μL)	10	

Gradient elution conditions:

Time (minutes)	Mobile phase A (%)	Mobile phase B (%)
0.2	90	10
0.5	90	10
2.0	5	95
3.0	5	95
4.0	90	10
5.9	90	10

MS (API6500+) conditions

Compound	WUSTL0717	I.S. (Carbamazepine)
MRM(+)	582/181	237.2/194.1
Collision Gas	7	
Curtain GAS	35	
Ion Source Gas1	55	
Ion Source Gas2	50	
Ion Spray Voltage	5500	
Temperature (°C)	550	
Collision Energy	41	26
Declustering Potential	25	136
Entrance Potential	10	
Collision Cell Exit Potential	14	

Supplementary Table 3. *In-Vitro* ADME parameters WUSTL0717.HCL

Compound	Kinetic Solubility (µM)	Mouse PPB (%bound)	HLM $t_{1/2}$ (minutes) / Cl_{int} (µl/minutes/mg)	MLM $t_{1/2}$ (minutes) / Cl_{int} (µl/minutes/mg)
WUSTL0717.HCl	0.29	99.81	12.4 / 112	49 / 18

PPB, plasma protein binding; HLM, human liver microsomes; MLM, mouse liver microsomes; $t_{1/2}$, half-life; Clint, intrinsic clearance.

Supplementary Table 4. qRT-PCR primers (mouse)

Primer (mouse)	Forward	Reverse
Col1a1	GACATCCCTGAAGTCAGCTGC	TCCCTTGGGTCCCTCGAC
bactin	GATCATTGCCTCCTGAGC	GTCATAGTCCGCCTAGAAGCAT
18S	GTAACCCGTTGAACCCCATT	CCATCCAATCGGTAGTAGCG

Supplementary Materials and Methods

Molecular modeling

WUSTL0717 and GW3965 were modeled in LXRα protein from reference structure PDBID: 3IPU and based on the coordinates of GW3965 in Xray structure 1PQ6.[1, 2] WUSTL0717 was modeled in LXRβ based on the coordinates of GW3965 in Xray structure 1PQ6. Charges were modeled using AM1BCC, energy minimization using Amber ff14SB, Steepest descent,[3] and conjugate gradient algorithms[4] implemented in UCSF Chimera.[5]

Ligand binding in LanthaScreen TR-FRET LXRβ coactivator assays

Binding assays of graded concentrations of WUSTL0717 or GW3965 to LXRβ were performed using the LanthaScreen TR-FRET LXRβ Coactivator kit (Thermo Fisher Scientific) following the manufacturer's protocol. Briefly, after the addition of 5 nM LXRβ-LBD to the compounds, a mixture of peptide (100 nM, Fluorescein-D22 for LXRβ) and antibody (10 nM, Tb-anti-GST) was added to the reaction followed by incubation in room temperature for 4 hours. TR-FRET ratio was calculated by dividing the emission at 520 nm by the emission at 495 nm using either Synergy 2 (BioTek) or FlexStation 3 (Molecular Devices) plate reader. The TR-FRET ratio was then normalized by dividing the ratio of each concentration by the averaged ratio of the positive control, which was set equal to 100%. Concentration-response curves were fitted using 4-variable non-linear regression.

Activation of transcriptional functions of LXRα and LXRβ

Plasmids LXR-α (79514; Addgene), LXR-β (79513; Addgene), and LXRE_Luc (177622; Addgene) were transfected into HEK293T cells (ATCC CRL-3216) using the Lipofectamine™ 3000 kit (L3000001; Thermo Fisher Scientific). 24 hours post-transfection, the cells were plated in a 96-well plate at a density of 30,000 cells per well. After an additional 24 hours, the cells were treated with WUSTL0717 or GW3965 for another 24 hours. Luciferase activity was then measured using a Synergy HTX (BioTek) plate reader with the ONE-Glo™ luciferase assay kit (E8130; Promega) at room temperature. Concentration-response curves were fitted using 4-variable non-linear regression.

Kinetic Solubility

Compound from a 10 mM DMSO stock solution was introduced to pre-warmed pH 7.4 phosphate buffered saline in a 96-well plate with a final DMSO concentration of 1%. The plate was maintained at 37°C for 24 hours on an orbital shaker and centrifuged through a Millipore Multiscreen Solvinter 0.45 micron low binding PTFE hydrophilic filter plate and analyzed by HPLC. Peak area was compared to standards of known concentration.

Plasma protein binding

Plasma protein binding was determined using equilibrium dialysis. All samples were tested in triplicate using the RED Rapid Equilibrium Dialysis Device (Thermo Fisher Scientific). The initial drug concentration in the plasma chamber was 2 µM, and phosphate buffered saline was added to the receiver chamber. The plate was covered and allowed to shake in a 37ºC incubator for 6 hours. 25 µl was sampled from the plasma and PBS chambers, which were then diluted with either blank PBS or plasma to achieve a 1:1 ratio or plasma:PBS for all samples. The concentration of the drug in the plasma and PBS chambers was determined by LC-MS/MS. The fraction bound was calculated as ([plasma] – [PBS]) / [plasma].

Hepatic microsomal stability

Microsome stability was evaluated by incubating 1 µM test compound with 1 mg/mL hepatic microsomes in 100 mM KPi, pH 7.4. The reaction was initiated by adding NADPH (1 mM final concentration). Aliquots were removed at 0, 5, 10, 20, 40, and 60 minutes and added to acetonitrile (5X, v:v) to stop the reaction and precipitate the protein. NADPH dependence of the reaction was evaluated by setting up incubations without NADPH. At the end of the assay, the samples were centrifuged through a Millipore Multiscreen Solvinter 0.45 micron low binding PTFE hydrophilic filter plate and analyzed by LC-MS/MS. Data were log-transformed and represented as half-life. The microsomes were purchased from B1oIVT. Human were av200 donor pool/mixed sex.

Metabolic cages

Mice were weighed, and their core body temperature was measured using a rectal thermometer. Body composition was assessed using EchoMRI-100H 2n1 (EchoMRI). Afterwards, the mice were housed individually in Comprehensive Laboratory Animal Monitoring System (CLAMS) metabolic cages (Columbus Instruments) for one day of acclimation, followed by 24 hours for recording. The cages were contained within a light-sealed environmental enclosure with LED white light strips next to each cage, which were turned on at 6:00am (Zeitbeber Time 0) and turned off at 6:00pm (Zeitgeber Time 12) each day for a 12h:12h light:dark cycle that matches that of the standard animal facility. The enclosure temperature was set a 22.2 °C. The airflow rate was set at 0.90 L/min, with an 18-second bleed of the air sampling tube followed by a 2-second measurement period for each cage, with sampling of enclosure air in each interval. Energy expenditure (heat) and RER were calculated from measurements of the volume of oxygen consumption and carbon dioxide production using indirect calorimetry with a zirconia O_2 sensor and CO_2 sensors. Ambulatory activity was measured using infrared laser detector arrays positioned along the X- and Y-axes at animal height.

qRT-PCR assays

Total RNA from tissues was extracted using Direct-zol RNA Miniprep Kits (R2052; Zymo Research) and was reverse-transcribed into cDNA using the High-Capacity cDNA Reverse Transcription Kit (4368814; Thermo Fisher Scientific). cDNA was amplified either TaqMan Fast Advanced Master Mix (4444554; Thermo Fisher Scientific) with the following probes: *Abca1* (Mm00442646_m1), *Srebf1* (Mm00550338_m1), *Scd1* (Mm00772290_m1), *Apoa1* (Mm00437569_m1), *Actb* (Mm02619580_g1), or *18S* (Hs99999901_s1), or SYBR Green qRT-PCR Master Mix (A46110; Thermo Fisher) with primer sequences listed in Supplementary Table 4. qRT-PCR was conducted using QuantStudio 6 Pro (Applied Biosystems).

Preparation of ileal mucosal protein for ApoA1 ELISA

Mucus collection was performed as previously described[6] with minor modifications. After flushing the small intestine with PBS, a 2-cm segment of ileum was dissected, opened lengthwise and pinned flat. Any remaining luminal contents were gently flushed with additional PBS rinses. The mucosal surface was subsequently rinsed with 2 mL of 10 mM Tris-HCl (pH 7.4) containing 5 mM $CaCl_2$, followed by gentle scraping using a cell lifter (08-100-240; Fisher Scientific). The collected mucus was transferred into 500 µL of lysis buffer [50 mM Tris-HCl (pH 7.5), 100 mM NaCl, 5 mM EDTA, 1% Triton X-100, and protease inhibitor]. The lysate was centrifuged twice at 13,000 rpm for 10 min to remove debris, and the final supernatant was stored at −20°C. Total protein was quantified by the Bradford assay, and 1:15 diluted samples were analyzed for ApoA1 levels using an ELISA kit (3750-1HP; Mabtech).

Intestinal lipid absorption and chylomicron secretion assay

Mice that were 9 to 11 weeks post-SBR with 6 to 8 weeks of WUSTL0717 administration, as well as non-operated mice that had received 6 weeks of WUSTL0717 treatment, underwent an intestinal triglyceride absorption assay. Mice were fasted in the dark cycle for 8–11 hours, then injected *i.v.* with 500 mg/kg body weight of Tyloxapol (T0307; Sigma-Aldrich) and then 10 µl/g body weight of olive oil (O1514; Sigma-Aldrich) via oral gavage one hour later. Blood was collected from tail veins at baseline and at 1, 2, 4, and 6 hours. Plasma triglycerides were measured using the L-Type TG-H kit (Wako Chemicals) read using a Cytation 5 Cell Imaging Multi-Mode Reader (BioTek).

Glucose uptake assay

Mice were 13 weeks post-SBR and had received 10 weeks of WUSTL0717 treatment before being fasted overnight for 15 hours. They were then administered a glucose solution (G8270; Sigma-Aldrich) via oral gavage at a dose of 2 g/kg body weight. Blood glucose levels were determined from the tail veins at baseline and 15, 30, 60, and 120 minutes after glucose injection using GLUCOCARD Vital (760001; Arkray).

Flow cytometry

Cells from blood stained with acridine orange were counted using an automated cell counter (Cellometer Auto T4; Nexelcom Bioscience). Red blood cells were lysed using BD Pharm Lyse (555899; BD Biosciences) for 8 minutes at room temperature, followed by centrifugation at 500 × g for 5 minutes. The lysis step was repeated 2–3 times as needed. The remaining cells were resuspended in PBS for 30 minutes. The cells were then stained with Live Zombie NiR (423106; Biolegends) in PBS for an additional 30 minutes to exclude dead cells. Next, the cells were stained with antibodies against cell surface markers for 30 minutes in FACs buffer consists of 2% FBS, 2 mM EDTA, 0.02% sodium azide, and 20% Brilliant buffer (563794; BD Biosciences) in PBS. Conjugated antibodies against CD45 (568336) and CD11b (612801) were sourced from BD Biosciences, and Ly6G to identify neutrophils was sourced from BioLegend (127641). Cells were acquired using the 5-lasers, 64-channels Cytek Aurora spectral flow cytometer (Cytek Biosciences) equipped with SpectroFlo software (version 3.1.0) and analyzed with FlowJo v10. All steps involving Live Zombie NiR staining, antibody staining, and fixation were performed on ice and in the dark.

Histological analysis

Liver and small intestine tissues were fixed in 4% paraformaldehyde, embedded in paraffin, and sectioned at a thickness of 4 µm. The sections were mounted on slides and stained with Hematoxylin & Eosin (H&E) for morphology analysis or with Sirius Red to assess collagen accumulation. Alternatively, 8-µm thick cryosections of snap-frozen liver samples were mounted on slides and stained with Oil Red O through Washington University Musculoskeletal Research Center. Slides were imaged using Zeiss Axio Scan Z1 (Zeiss) and analyzed using Zen and Fiji-ImageJ software. For the analysis of SHG, deparaffinized liver tissue slides were subjected to 2-photon microscopy. Two-photon microscopy images were collected using a customized dual-laser system (InSight® & Mai Tai®, Spectra-Physics) on a Leica SP8 upright microscope equipped with a 25x, 0.95 numerical aperture water-immersion objective. The Mai Tai® laser was tuned to 920 nm, and signal separation was achieved using three long-pass dichroic beam splitters (FF640-FDi01, FF562-FDi03, and FF495-Di03, Semrock) to generate channels at approximately 390–495 nm (SHG), 495–562 nm, and 562–640 nm. Fluorescence emission was directed to external hybrid photodetectors (Leica). Analysis of the second harmonic signal was carried out using Imaris software in combination with Fiji-ImageJ software.

RNA sequencing

Total RNA integrity was determined using an Agilent Bioanalyzer or 4200 Tapestation. Library preparation was performed with 500 ng to 1 µg of total RNA. Ribosomal RNA was removed using an RNase H method with RiboErase kits (Kapa Biosystems). mRNA was then fragmented in reverse transcriptase buffer by heating to 94°C for 8 minutes. The fragmented mRNA was reverse transcribed to yield cDNA using SuperScript III RT enzyme (Life Technologies) and random hexamers. A second-strand reaction was performed to yield double-stranded cDNA. cDNA was blunt-ended, had an A base added to the 3' ends, and Illumina sequencing adapters were ligated to the ends. Ligated fragments were then amplified for 12–15 cycles using primers incorporating unique dual index tags. Fragments were sequenced on an Illumina NovaSeq X Plus using paired-end reads extending 150 bases. Base calling and demultiplexing were performed with Illumina's bcl2fastq software with a maximum of one mismatch in the indexing read. RNA-seq reads were then aligned to the Ensembl release 101 primary assembly with STAR version 2.7.9a1. Gene counts were derived from the number of uniquely aligned unambiguous reads by Subread:featureCounts (version 2.0.32). Sequencing performance was assessed for the total number of aligned reads, the total number of uniquely aligned reads, and features detected. The ribosomal fraction, known junction saturation, and read distribution over known gene models were quantified with RSeQC version 4.04, and RNA-seq was conducted by the Genome Technology Access Center. All gene counts were then imported into the R/Bioconductor package EdgeR5, and TMM normalization size factors were calculated to adjust for differences in library size across samples. For downstream analysis, the TPM files were directly loaded into R for further analysis. Gene names were standardized, and duplicate entries were removed to retain unique gene features. Genes with zero TPM values across all samples were excluded. To reduce the impact of extreme values and facilitate comparisons, the TPM data were log2-transformed. Batch effects between experimental groups were

corrected using the ComBat function from the sva R package. Normalized data were visualized with boxplots to confirm consistent sample distributions and ensure comparability across datasets. Filtered TPM matrices were then subjected to downstream analyses. DEGs were identified by comparing experimental groups. Log_2FC and p-values were calculated for each gene using unpaired two-sample t-tests and Wilcoxon rank-sum tests. Genes were ranked by log_2FC and p-values, and the Benjamini–Hochberg procedure was used to control the false discovery rate (FDR). Genes with an adjusted *P*-value < .05 and an absolute log_2FC above a set threshold were considered significantly differentially expressed. Preranked GSEA was performed using the GSEA_4.0.3 with MSigDB collections. Significant enrichment was determined by normalized enrichment scores (NES) and adjusted FDR q-values <0.05. GSVA scores were calculated across samples and standardized (z-score normalization) using custom gene sets, including those associated with LXR signaling, which were defined using the GSEABase package. PCA, heatmaps, enrichment plots, and volcano plots, were generated using R (ggplot2, gplots, dplyr, EnhancedVolcano).

16S rRNA sequencing

DNA was extracted using the ZymoBIOMICS®-96 MagBead DNA Kit (Zymo Research). Library preparation for targeted 16S sequencing was performed using the Quick-16S™ Plus NGS Library Prep Kit with V3–V4 primers. Amplification was performed using a qPCR–based method to minimize chimera formation. PCR products were quantified, pooled at equal molarity, and purified. Final libraries were quantified and sequenced on an Illumina® NextSeq 2000™ (600-cycle P1 kit). Positive controls (ZymoBIOMICS® Microbial Community Standard or DNA Standard) and negative controls (blank extraction and library controls) were included to monitor contamination. Sequencing reads were processed with the DADA2 pipeline for denoising and chimera removal. Taxonomy was assigned using UCLUST from QIIME v.1.9.1 with the Zymo Research 16S reference database. Diversity and composition analyses were conducted using QIIME v.1.9.1, and Linear Discriminant Analysis Effect Size (LEfSe) was used to identify taxa with significant group differences. Sequencing and analysis were performed by Zymo Research.

Lipid and metabolite extraction and LC-MS/MS Analysis

Portal venous serum from female mice was collected. Sample preparation and LC-MS/MS for lipid and metabolite profiling were subsequently performed by the Mass Spectrometry Technology Access Center at the McDonnell Genome Institute. Samples were subjected to biphasic extraction using methyl tert-butyl ether. Samples were vortexed 3 times and incubated at –80°C for 1 hour. After phase separation, the upper phase containing lipids was collected and dried using a SpeedVac without applying heat. The lower phase containing polar metabolites was centrifuged, and the resulting supernatant was similarly dried. Dried lipid extracts were reconstituted in 19 µL of methanol:acetonitrile:water (2:1:1, v/v/v), and dried metabolite extracts were reconstituted in 19 µL of 50% methanol. Reconstituted samples were analyzed by LC-MS/MS using a Vanquish Horizon UHPLC system (Thermo Fisher Scientific) equipped with C8 and C18 columns, and coupled to an Orbitrap Tribrid ID-X mass spectrometer (Thermo Fisher Scientific) operating in both positive and negative ion modes with the AcquireX DeepScan workflow. In total, 2,293 features were annotated as metabolites and lipid species. Quantification was performed based on MS1 peak areas (AUC). Imputed data were used for downstream analyses, and statistical analysis and plot generation were performed using R. For PCA, the imputed data were processed by log_{10} transformation, interquartile range filtering at 40%, sum normalization, and autoscaling. Metabolites with an FDR < 0.2 and an FC > 1.5 in SBR-WUSTL0717 compared to SBR were log_{10}-transformed and used for heatmap generation. For generating pie charts and for analyses of individual significant metabolites, we followed the workflow typically used for volcano plots: data were processed by log_2 transformation and sum normalization without autoscaling, and significant metabolites were defined as those with an FDR < 0.1 and an FC > 1.5, according to the standard MetaboAnalyst (https://metaboanalyst.ca) workflow. Finally, the mean log_{10} value of each group was used to analyze the Pearson correlation with liver collagen accumulation.

References

1. Färnegårdh M, Bonn T, Sun S, et al. The three-dimensional structure of the liver X receptor beta reveals a flexible ligand-binding pocket that can accommodate fundamentally different ligands. J Biol Chem 2003;278:38821-8.
2. Fradera X, Vu D, Nimz O, et al. X-ray structures of the LXRalpha LBD in its homodimeric form and implications for heterodimer signaling. J Mol Biol 2010;399:120-32.
3. Meza JC. Steepest descent. WIREs Computational Statistics 2010;2:719-722.
4. Nazareth JL. Conjugate gradient method. WIREs Computational Statistics 2009;1:348-353.
5. Pettersen EF, Goddard TD, Huang CC, et al. UCSF Chimera--a visualization system for exploratory research and analysis. J Comput Chem 2004;25:1605-12.
6. Mukherjee P, Chattopadhyay A, Grijalva V, et al. Oxidized phospholipids cause changes in jejunum mucus that induce dysbiosis and systemic inflammation. J Lipid Res 2022;63:100153.

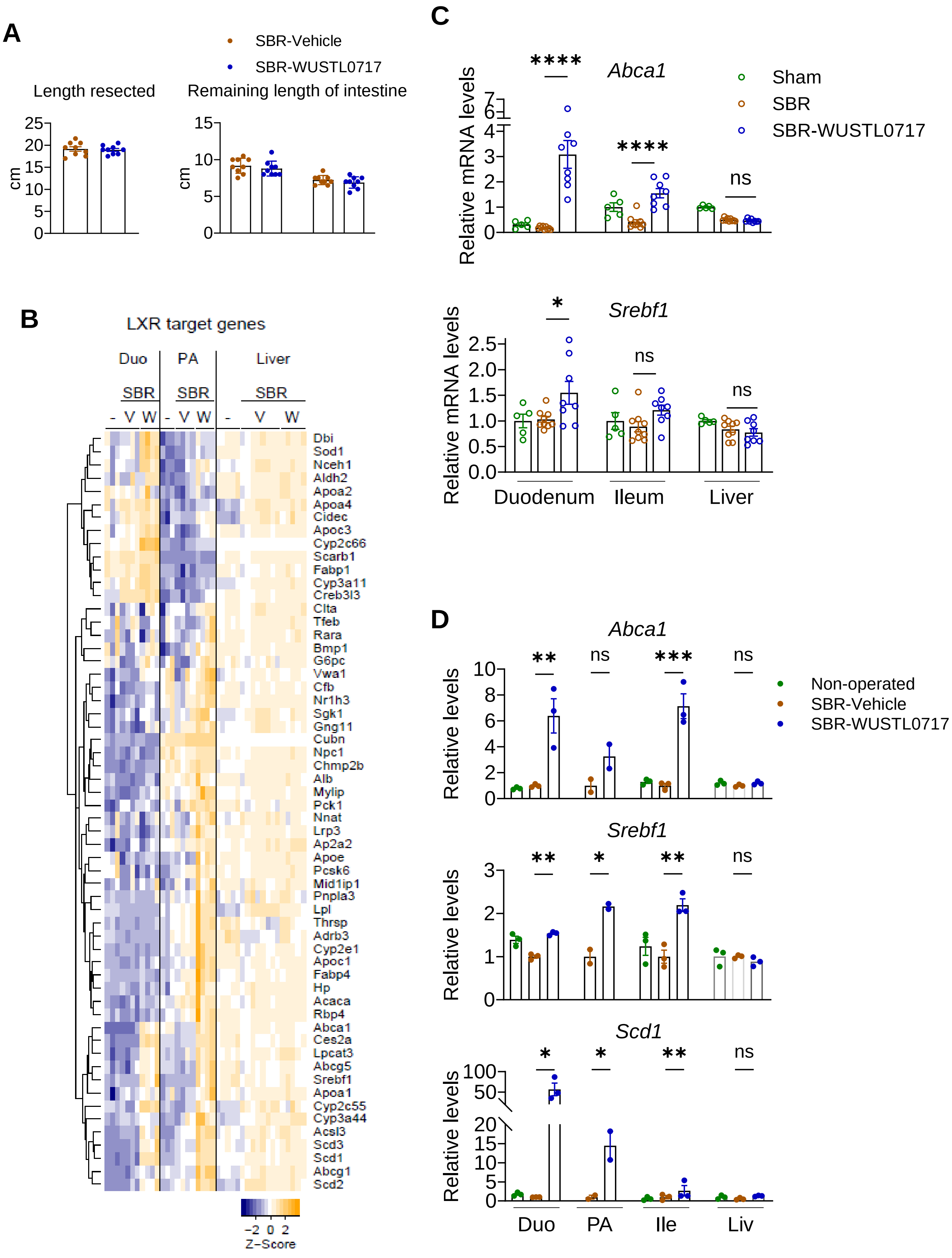

Supplementary Figure 1

Supplementary Figure 1. Intestine-restricted LXR agonist activity following treatment with WUSTL0717.

(A–C) WT male (filled circles) or female (open circles) mice underwent sham or SBR surgery. Starting 3 weeks post-surgery, the mice were treated daily with vehicle or WUSTL0717 (30 mg/kg, p.o.) for 7 weeks (n = 5–9/group).

(A) Length of the small intestine resected during SBR and remaining length at 10 weeks post-SBR.

(B) Heatmap showing LXR target gene expression in the duodenum (Duo), post-anastomosis ileum (PA), and liver (Liv), based on RNA-seq. V, vehicle; W, WUSTL0717.

(C) Transcript levels of LXR target genes in the duodenum, post-anastomosis ileum, or liver, measured by qRT-PCR.

(D) WT male mice (n = 2–3/group) underwent sham or SBR surgery and were treated with vehicle or WUSTL0717 starting 5 days post-surgery for 10 days before sacrifice.

Statistical analysis was performed using one-way ANOVA with Dunnett's (C) or Tukey's HSD (D) test for multiple comparisons. Data are presented as mean ± SEM; **P* < .05, ***P* < .01, ****P* < .001, *****P* < .0001; ns, not significant. Mean ± SEM and p-value thresholds are applied consistently across figures unless otherwise noted.

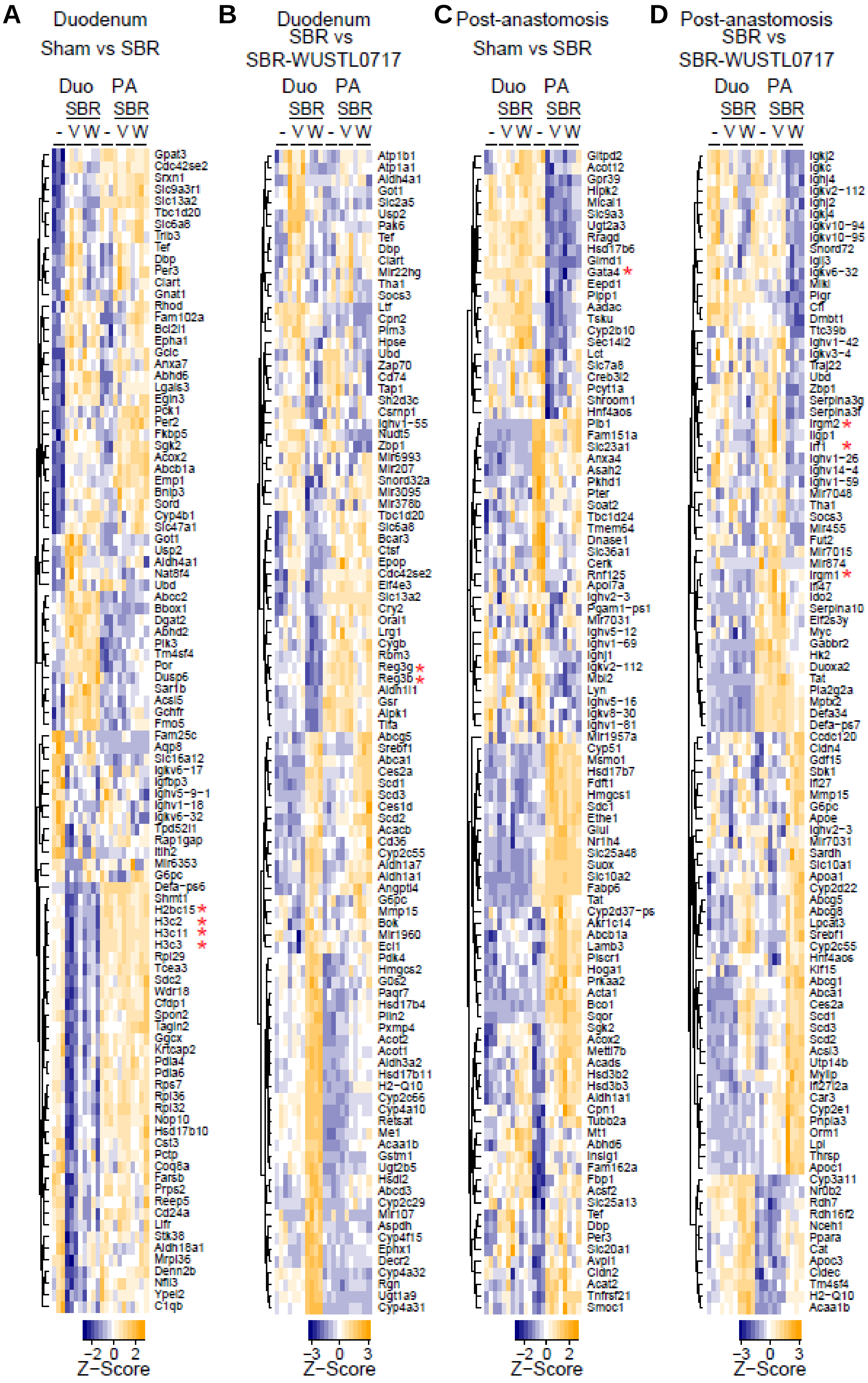

Supplementary Figure 2

Supplementary Figure 2. Intestinal adaptation following SBR and WUSTL0717 treatment.

(A–D) WT male mice underwent sham or SBR surgery. 3 weeks later, mice were treated daily with vehicle or WUSTL0717 (30 mg/kg, p.o.) for 7 weeks before euthanasia. Heatmaps show the top 50 DEGs ($p < 0.05$, FC ≥ 2) identified by RNA-seq in the duodenum (A, B) and post-anastomosis ileum (C, D). Comparisons are sham vs. SBR (A, C) and vehicle- (V) vs. WUSTL0717-treated (W) mice following SBR (B, D) (n = 3–4/group).

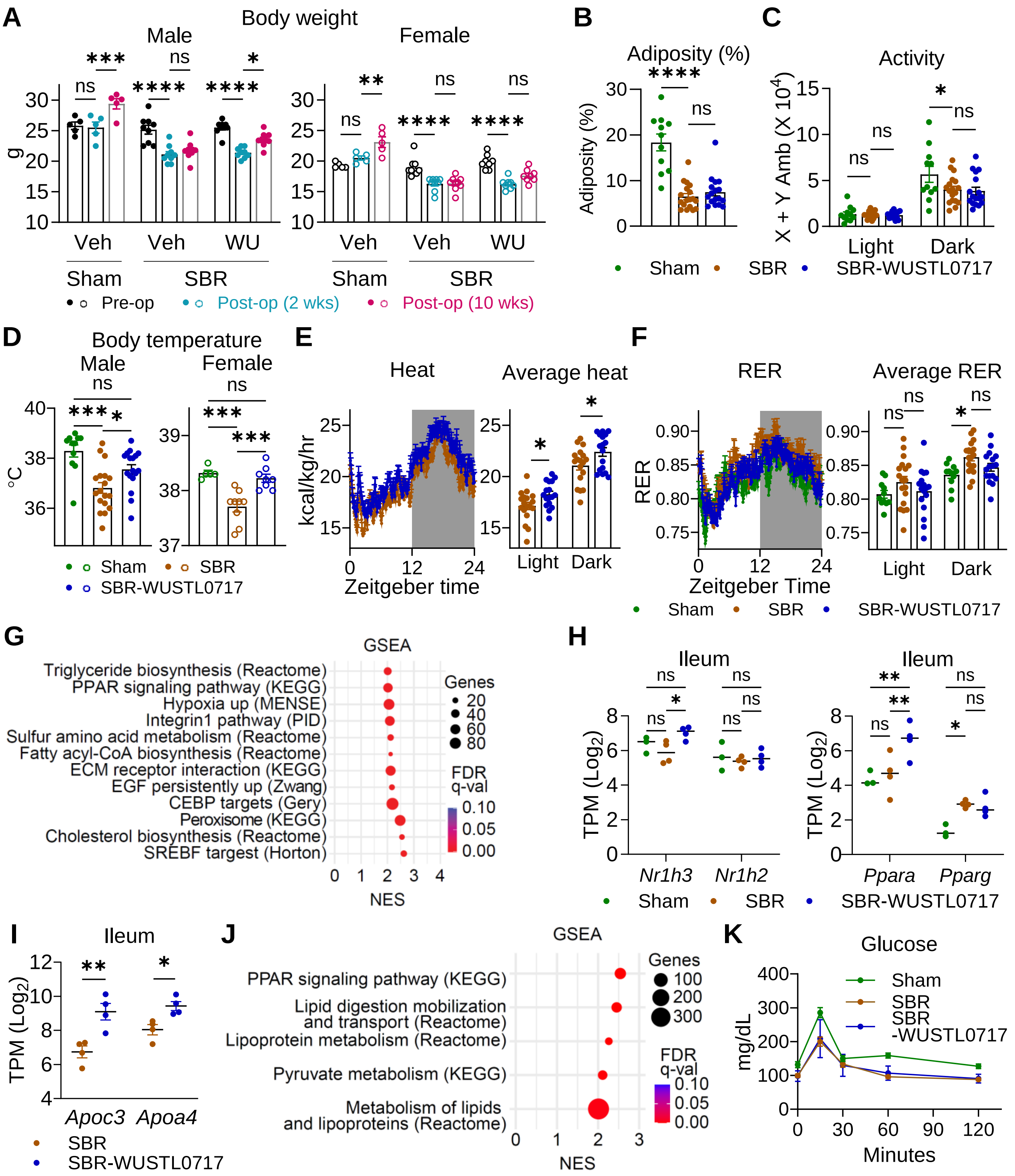

Supplementary Figure 3

Supplementary Figure 3. Metabolic phenotype of WUSTL0717 treatment in WT mice following SBR.

WT male (filled circles) or female (open circles) mice underwent sham or SBR surgery. Beginning 3 weeks post-surgery, mice received vehicle or WUSTL0717 (30 mg/kg, p.o.) daily for up to 7 weeks before euthanasia, unless otherwise specified (I). For the glucose tolerance test (I), treatment continued for 10 weeks, and measurements were performed at 13 weeks post-surgery.

(A) Body weight of mice measured at the indicated weeks (n = 5–9/group). Veh, vehicle; WU, WUSTL0717.

(B–F) At 8–9 weeks post-operation, mice treated with vehicle or WUSTL0717 for 5–6 weeks were subjected to adiposity measurement (B) and core body temperature assessment (D). Mice were individually housed in metabolic cages for a 24-hour evaluation of locomotor activity (C), heat generation (E) and RER (F). The time traces in panels E and F represent group mean values over time, while the adjacent bar plots show the corresponding light- and dark-phase averages for each individual mouse, with each dot representing one mouse. Data from male mice were combined from two independent experiments (male: n = 11–18 /group; female: n = 5–9/group).

(G, J) GSEA of RNA-seq data from post-anastomosis ileum, with enrichment plots illustrating normalized enrichment scores (NES) and associated gene signatures from GSEA of RNA-seq data. (G) Signatures upregulated in SBR relative to sham; (J) signatures upregulated in WUSTL0717-treated group compared to vehicle following SBR (n = 3–4/group).

(H, I) Transcript levels linked to proximal intestinal identity in the post-anastomosis ileum, analyzed by RNA-seq (n = 3–4/group).

(K) Blood glucose levels were measured in overnight-fasted mice 13 weeks post-surgery at 0, 15, 30, 60, and 120 minutes after a 2 g/kg glucose gavage (n = 8–11/group).

Unpaired Student's t-test (E, I), one-way ANOVA (B, D), or two-way ANOVA with Tukey's HSD (A, C, F, H, K) was used for statistical analysis.

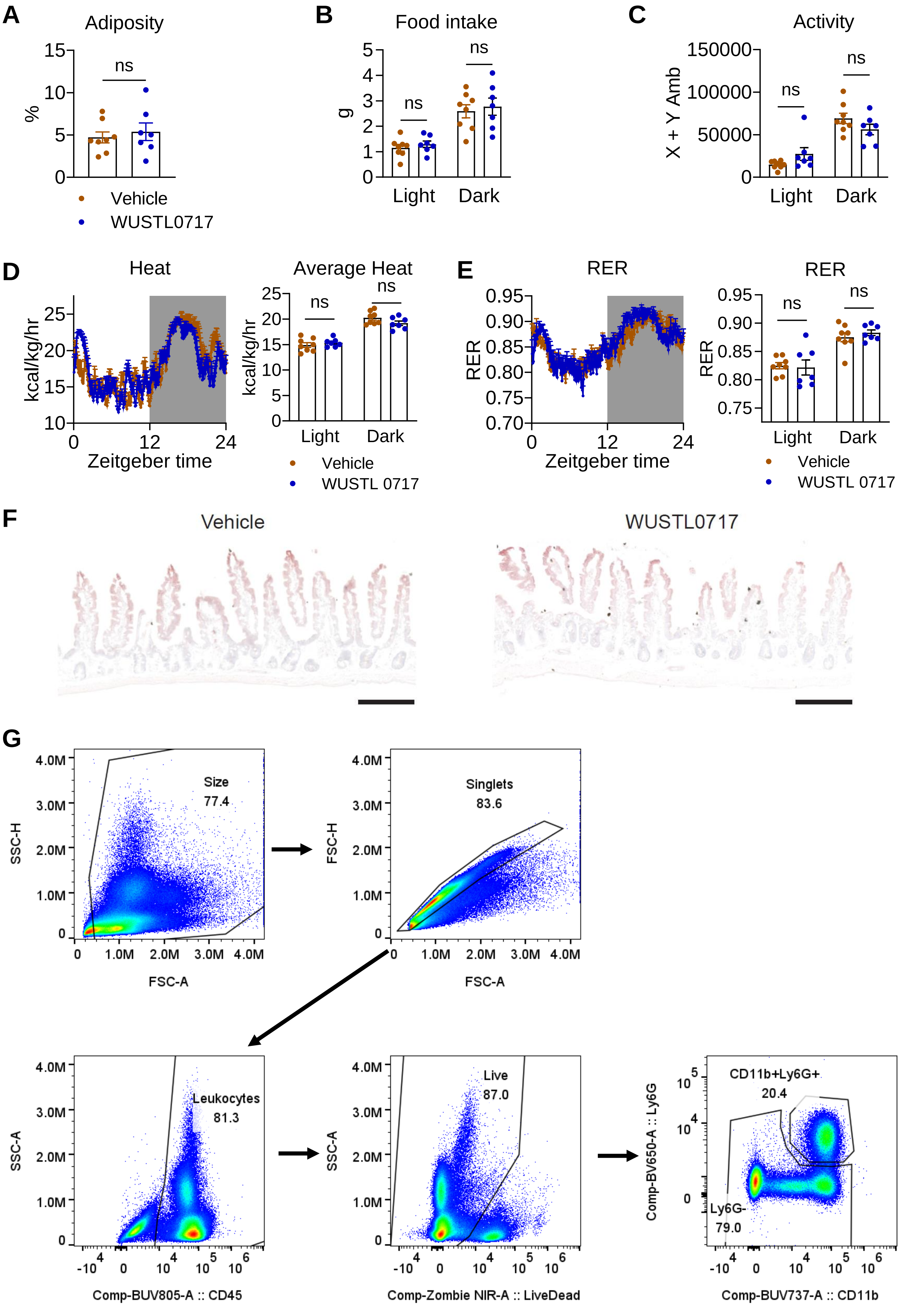

Supplementary Figure 4

Supplementary Figure 4. Metabolic phenotype of WUSTL0717 treatment in WT mice.

(A–G) WT male mice treated daily with either vehicle (n = 8) or WUSTL0717 (30 mg/kg, p.o.) (n = 7) for up to 7 weeks before euthanasia.

(A–E) After 5 weeks of treatment, mice underwent adiposity measurement (A) and were then individually housed in metabolic cages for 24-hour assessment of food intake (B), locomotor activity (C), heat generation (D), and RER (E) under a 12-hour light-dark cycle.

(F) Representative Oil Red O staining of the jejunum from each group after euthanasia (scale bar: 200 μm). Images shown are representative of evaluations from 8 vehicle- and 7 WUSTL0717-treated mice.

(G) Representative flow cytometry plots showing the gating strategy for blood cell suspensions collected after euthanasia, referenced to Figure 4H.

Unpaired Student's t-test was used for statistical evaluation.

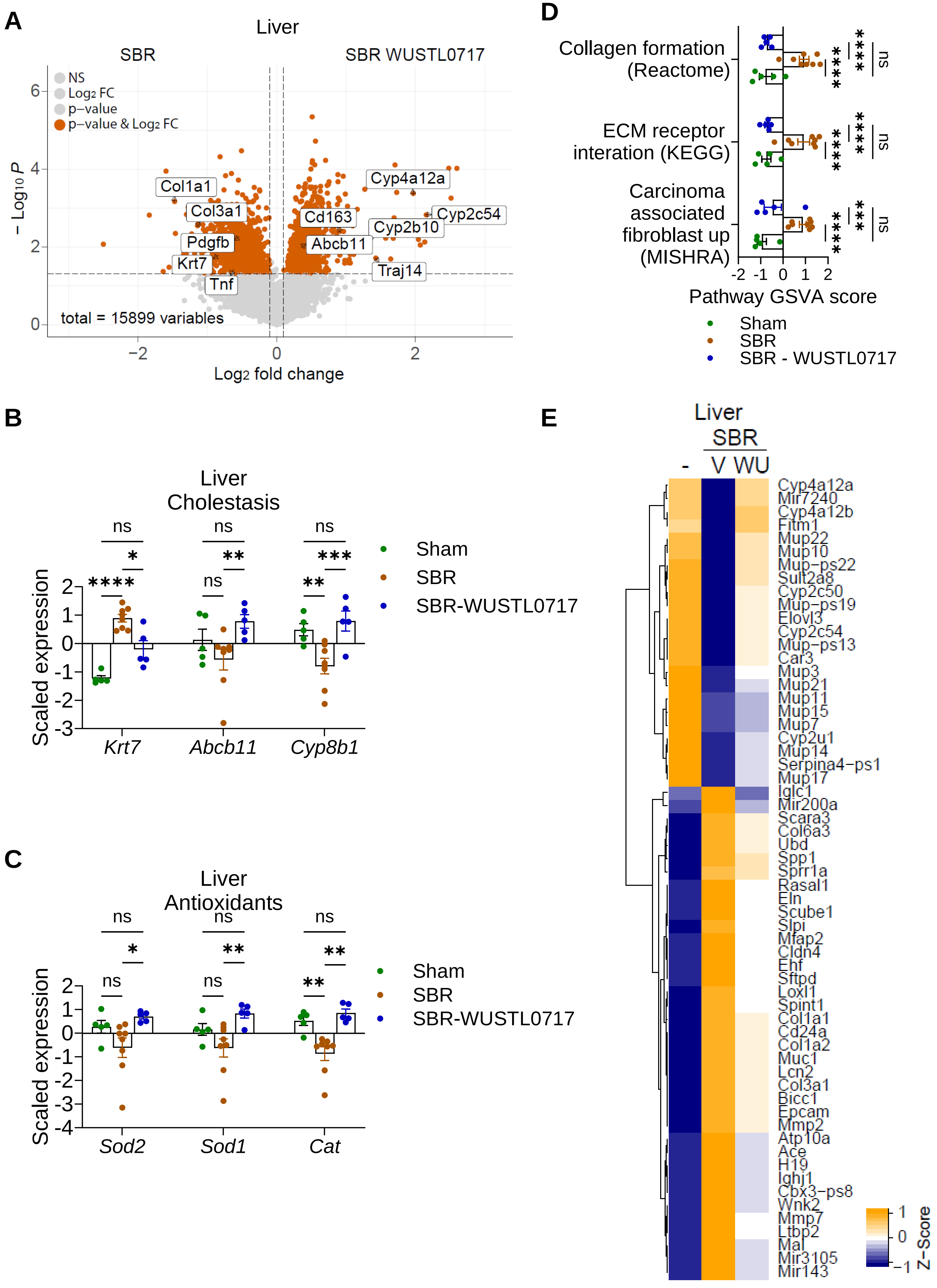

Supplementary Figure 5

Supplementary Figure 5. Analysis of DEGs associated with liver fibrosis in sham or SBR, WUSTL0717-treated groups.

(A, B, D–F) RNA-seq was performed on liver tissue from WT male mice that underwent sham or SBR surgery and were treated daily with vehicle or WUSTL0717 (30 mg/kg, p.o.) for 7 weeks (n = 5–8/group).

(A) Volcano plot showing DEGs in the liver between vehicle- and WUSTL0717-treated groups following SBR. DEGs were identified based on an adjusted p-value < 0.05 and $\log_2$FC thresholds, with significant genes highlighted in orange.

(B) Transcript levels of *Krt7, Abcb11*, and *Cyp8b1* in the liver.

(C) Transcript levels of *Sod2*, *Sod1*, and *Cat* in the liver.

(D) GSVA scores for liver fibrosis-related pathways.

(E) Heatmap of DEGs in the liver, showing the up- or downregulated genes with fold change (*P*-value < .05 and FC ≥ 2) when comparing the vehicle and WUSTL0717-treated groups following SBR. The heatmap also highlights genes with significant differences (*P*-value < .05) between sham and SBR groups. For visualization, a single group mean was normalized to a z-score and shown in the heatmap.

Statistical evaluations used two-way ANOVA (B–D) with Tukey's HSD.

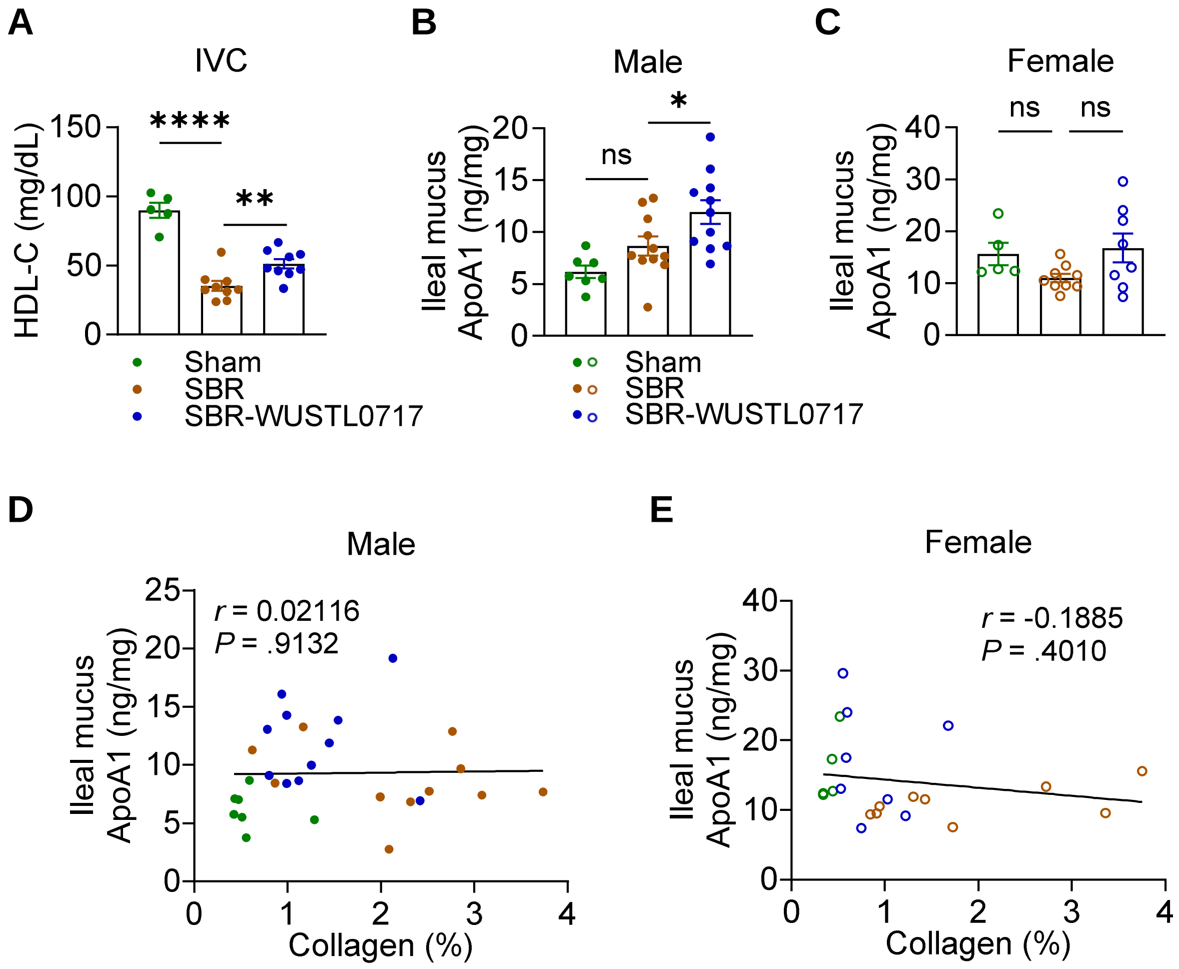

Supplementary Figure 6

Supplementary Figure 6. HDL-C and correlation of ileal mucus ApoA1 with liver fibrosis after WUSTL0717 treatment following SBR.

(A–E) WT male (filled circles) and female (open circles) mice underwent sham or SBR surgery. 3 weeks later, the mice received vehicle or WUSTL0717 (30 mg/kg, p.o.) daily for 7 (A, C, E) or 12 (B, D) weeks, after which they were euthanized for analysis (n = 5–11/group).

(A) Systemic plasma HDL-C levels (IVC) in each group.

(B, C) ApoA1 levels in the ileal mucus layer measured by ELISA and normalized to total protein content.

(D, E) Correlation between ileal mucosal ApoA1 levels (B, C) and liver collagen area (Figure 5C). Each dot represents a matched individual from panels B and C.

Statistical evaluations were performed using unpaired Student's t-test (A), one-way ANOVA with Tukey's HSD (B, C), or Pearson correlation (D-E).

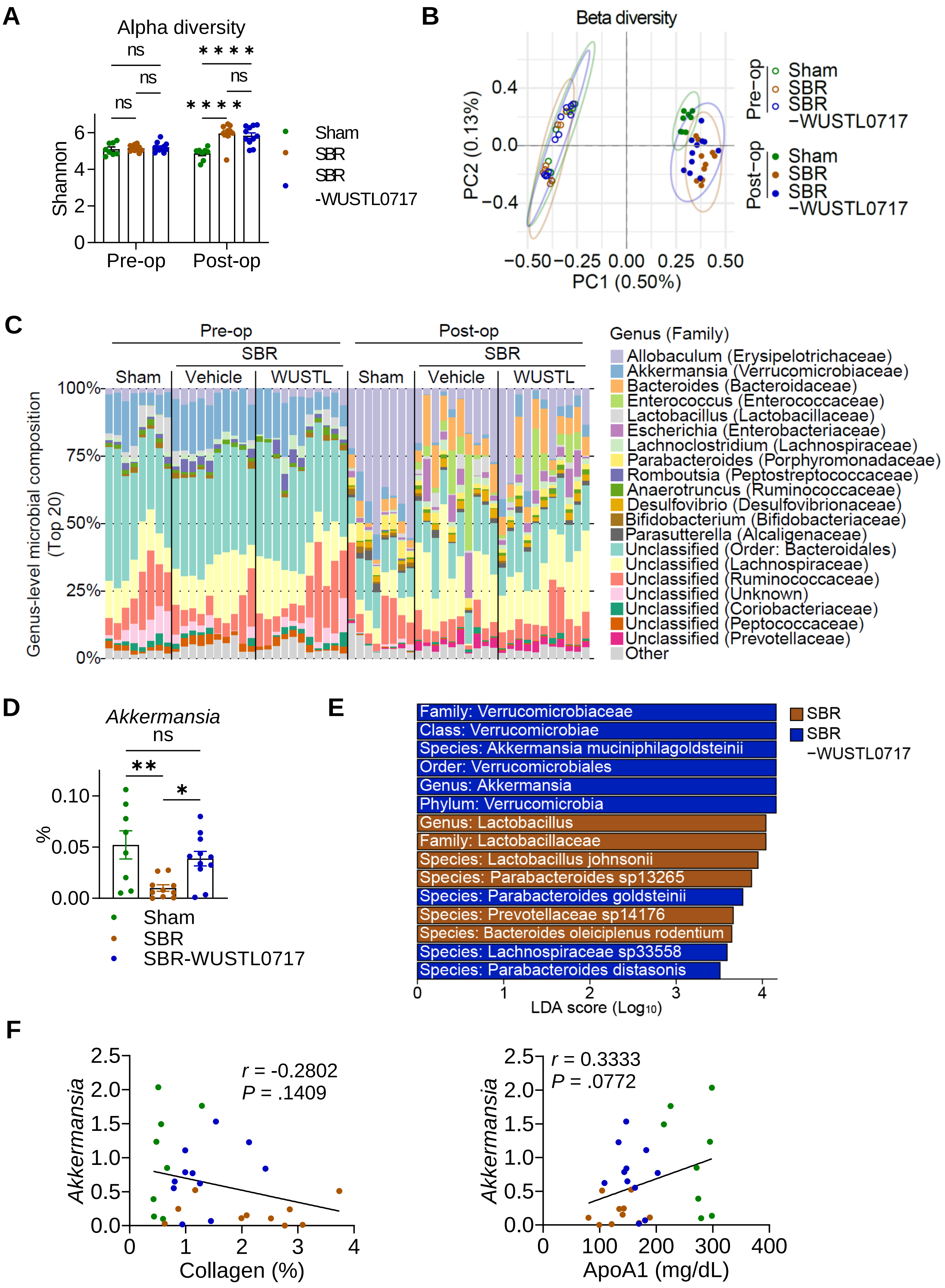

Supplementary Figure 7

Supplementary Figure 7. Fecal microbiome analysis of WUSTL0717-treated mice after SBR.

16S rRNA sequencing was performed on fecal samples from WT male mice collected before surgery and 10 weeks after sham or SBR surgery, following daily treatment with vehicle or WUSTL0717 (30 mg/kg, p.o.) for 7 weeks (n = 8–11/group).

(A) Shannon alpha diversity index.

(B) Bray–Curtis beta diversity index.

(C) Top 20 genera in the microbial composition. Genus and family information are shown in the figure for each group.

(D–F) Analysis of post-operative groups at 10 weeks after surgery.

(D) Percentage of *Akkermansia* in the total microbial composition.

(E) Taxa with LEfSe LDA scores > 3.5 are displayed on a log scale.

(F) Correlation between *Akkermansia* abundance (D) and either liver collagen area or portal venous plasma ApoA1 levels. Each dot represents an individual sample matched to panel D.

One-way ANOVA (D), two-way ANOVA (A) with Tukey's HSD, and Pearson correlation (F) were used for statistical analyses.

Supplementary Table 1. Genotyping primers (mouse)

Primer (mouse)	Forward	Reverse
Apoa1 flox	CGAAGTTATGAATTCTATCTCGCACCT	TGACCAGGATCCATAACTTCGTATAATGT
Apoa1 flox-WT	ACCGTGGATATCTCGCACCTT	TCTGACCAGTACTGGGGGTTA
Villin-Cre	GCCTTCTCCTCTAGGCTCGT	AGGCAAATTTTGGTGTACGG
		TATAGGGCAGAGCTGGAGGA (Internal primer)

Supplementary Table 2. WUSTL0717 instrument settings

LC (Shimadzu UFLC XR) conditions

Compound	WUSTL0717	I.S. (Carbamazepine)
Column	Thermo Betasil C18 5μ, 50x2.1mm	
Mobile phase	A: Water with 0.1% Formic Acid<br>B: Acetonitrile with 0.1% Formic Acid	
Flow rate (mL/minutes)	0.35	
Temperature (°C)	35	
Injection volume(μL)	10	

Gradient elution conditions:

Time (minutes)	Mobile phase A (%)	Mobile phase B (%)
0.2	90	10
0.5	90	10
2.0	5	95
3.0	5	95
4.0	90	10
5.9	90	10

MS (API6500+) conditions

Compound	WUSTL0717	I.S. (Carbamazepine)
MRM(+)	582/181	237.2/194.1
Collision Gas	7	
Curtain GAS	35	
Ion Source Gas1	55	
Ion Source Gas2	50	
Ion Spray Voltage	5500	
Temperature (°C)	550	
Collision Energy	41	26
Declustering Potential	25	136
Entrance Potential	10	
Collision Cell Exit Potential	14	

Supplementary Table 3. *In-Vitro* ADME parameters WUSTL0717.HCL

Compound	Kinetic Solubility (µM)	Mouse PPB (%bound)	HLM $t_{1/2}$ (minutes) / Cl_{int} (µl/minutes/mg)	MLM $t_{1/2}$ (minutes) / Cl_{int} (µl/minutes/mg)
WUSTL0717.HCl	0.29	99.81	12.4 / 112	49 / 18

PPB, plasma protein binding; HLM, human liver microsomes; MLM, mouse liver microsomes; $t_{1/2}$, half-life; Clint, intrinsic clearance.

Supplementary Table 4. qRT-PCR primers (mouse)

**Primer (mouse)**	**Forward**	**Reverse**
Col1a1	GACATCCCTGAAGTCAGCTGC	TCCCTTGGGTCCCTCGAC
bactin	GATCATTGCCTCCTGAGC	GTCATAGTCCGCCTAGAAGCAT
18S	GTAACCCGTTGAACCCCATT	CCATCCAATCGGTAGTAGCG
